# Supplementary material for: Teleultrasound in obstetrics: A systematic review and meta-analysis
Source: PLoS Med. 2026 Feb 6;23(2):e1004922. doi: 10.1371/journal.pmed.1004922 (PMC12900445; doi:10.1371/journal.pmed.1004922)
Supplement: S2 Table — (DOCX) [file pmed.1004922.s002.docx]

**S2 Table:** Search Strategy.

Database: MEDLINE and Embase

December 2025

| Set | Search Term |
| --- | --- |
| S1 | ultrasound* OR sonograph* OR sonography OR telesonography* OR ultrasonography OR Doppler ultrasonography OR ultraso* |
| S2 | remote consultation OR remote consult* OR remote OR remotely supported OR remotely guided OR tele* OR Telecommunications OR telemedicine OR teleultrasound OR ambulatory OR home or community OR cell phone OR mobile phone OR mobile OR Internet OR telecommunications |
| S3 | obstetrics OR prenatal care OR pregnancy OR matern* OR prenatal* OR antenatal* OR antepartum* OR pregnan* OR obstetric* |
| S4 | fetus or fetal |
| S5 | S1 and S2 and S3 and S4 and S4 |
| Total results | 2354 |
