# Supplementary material for: Teleultrasound in obstetrics: A systematic review and meta-analysis
Source: PLoS Med. 2026 Feb 6;23(2):e1004922. doi: 10.1371/journal.pmed.1004922 (PMC12900445; doi:10.1371/journal.pmed.1004922)
Supplement: S4 Table — (DOCX) [file pmed.1004922.s004.docx]

**S4 Table:** Excluded studies and associated reasons for exclusion.

| Author | Year | Title | Reason for the exclusion |
| --- | --- | --- | --- |
| Aeberli | 2018 | Real-time tele-training for obstetrical ultrasonography in Congo Brazzaville | Unable to translate |
| Arbeille | 2008 | Realtime tele-operated abdominal and fetal echography in 4 medical centres, from one expert center, using a robotic Arm & ISDN or satellite link | Duplicate paper |
| Arroyo | 2022 | No sonographer, no radiologist: New system for automatic prenatal detection of fetal biometry, fetal presentation, and placental location | Does not involve teleultrasound |
| Axelrod | 2023 | Putting the "f"etus back in maternal-fetal telemedicine: a prospective pilot study | Wrong publication type |
| Axelrod | 2024 | 384 Digital intervention and hybrid care improves adherence to gestational diabetes surveillance - prospective pilot study | Wrong publication type |
| Bachnas | 2025 | Fetal cardiac diagnostics in Indonesia: a study of screening and echocardiography | Wrong publication type |
| Balfour | 1978 | Use of portable real-time ultrasound scanner in district antenatal clinics | Does not involve teleultrasound |
| Bidmead | 2017 | Fetal telemedicine pilot-a study of clinical stakeholder acceptance of a tele-ultrasound innovation linking a rural district general hospital with a fetal medicine unit at a major teaching hospital in northern England | Wrong publication type |
| Bolin | 2016 | Reaching high-risk and underserved mothers: fetal cardiac evaluation by telemedicine is an effective screening tool | Wrong publication type |
| Bottalico | 2009 | Second-trimester genetic sonogram for detection of fetal chromosomal abnormalities in a community-based antenatal testing unit | Does not involve teleultrasound |
| Bravo-Valenzuela | 2024 | Three-Dimensional Ultrasound for Physical and Virtual Fetal Heart Models: Current Status and Future Perspectives | Wrong publication type |
| ButlerTobah | 2019 | Randomized comparison of a reduced-visit prenatal care model enhanced with remote monitoring | Does not involve teleultrasound |
| Buyon | 2024 | Prospective evaluation of high titer autoantibodies and fetal home monitoring in the detection of atrioventricular block among anti-SSA/Ro pregnancies | Does not involve teleultrasound |
| Chourasia | 2012 | Implementation of foetal e-health monitoring system through biotelemetry | Does not involve teleultrasound |
| CrispinMilart | 2016 | Use of a portable system with ultrasound and blood tests to improve prenatal controls in rural Guatemala | Does not involve teleultrasound |
| Cuneo | 2017 | Heart sounds at home: feasibility of an ambulatory fetal heart rhythm surveillance program for anti-SSA-positive pregnancies | Does not involve teleultrasound |
| Dalmacion | 2018 | Handheld ultrasound to avert maternal and neonatal deaths in 2 regions of the Philippines: An iBuntis intervention study | Does not involve teleultrasound |
| Fisk | 1993 | Intercontinental fetal surgical consultation with image transmission via Internet | Wrong study design |
| Freire | 2018 | Home fetal heart rate monitoring for surveillance of fetal arrhythmias: A cohort analysis | Wrong publication type |
| Freire | 2017 | Evaluation of home fetal heart rate doppler monitoring for surveillance of fetal arrhythmias | Wrong publication type |
| Fuentes | 2003 | Remote interpretation of ultrasound images | Wrong publication type |
| Geerts | 2004 | A community-based obstetric ultrasound service | Does not involve teleultrasound |
| Greenwold | 2014 | Implementing an obstetric ultrasound training program in rural Africa | Wrong study design |
| Guendelman | 2023 | Use of telehealth during the COVID-19 pandemic among practicing maternal-fetal medicine clinicians | Wrong study design |
| Gyselaers | 2006 | Community-based screening for Down syndrome in the first trimester using ultrasound and maternal serum biochemistry | Wrong publication type |
| Hadlow | 2006 | Community-based screening for Down syndrome in the first trimester using ultrasound and maternal serum biochemistry | Wrong publication type |
| Haeri | 2022 | Maternal-fetal telemedicine: the impact of a national hub and spoke model on critical access communities | Wrong publication type |
| IEEE | 2014 | An mHealth Approach to remote fetal monitoring | Wrong intervention |
| IEEE | 2016 | Automatic measurement of the fetal abdominal section on a portable ultrasound machine for use in low and middle income countries | Does not involve teleultrasound |
| IEEE | 2020 | Fast ellipse fitting implementation on USG mobile telehealth application | Does not involve teleultrasound |
| Joshi | 2023 | Effectiveness of focused obstetric ultrasound training to nurses from remote health posts to improve pregnancy outcome and reduce morbidity | Does not involve teleultrasound |
| Kanno | 2017 | Build-out remotely connected support system of fetal diagnosis | Wrong publication type |
| Katebi | 2023 | Hierarchical attentive network for gestational age estimation in low-resource settings | Does not involve teleultrasound |
| Kyle | 2014 | Validation of a tele-cardiology network for congenital heart disease | Wrong publication type |
| LaRosa | 2022 | Did the sonographer catch it? Performance impact of maternal-fetal telemedicine on ultrasounds within OBGYN practices | Wrong publication type |
| Lie | 2017 | Patients' views of a fetal ultrasound telemedicine service: A mixed methods evaluation study | Wrong publication type |
| Li | 2025 | Artificial intelligence-driven framework for improving prenatal screening for congenital heart disease in rural Nebraska | Wrong study design |
| Mary | 2023 | Perinatal telemedicine at lower-level birthing hospitals in Maryland: lessons learned from a landscape analysis | Does not involve teleultrasound |
| McClure | 2014 | First look: A cluster-randomized trial of ultrasound to improve pregnancy outcomes in low income country settings | Does not involve teleultrasound |
| Meizner | 2025 | Transforming obstetric care: The impact of patient-operated home ultrasound and remote diagnosis in hybrid prenatal care - Clinical insights from three years of research and real-world application | Wrong publication type |
| Morris | 2000 | Enhancement of an antenatal diagnosis and counselling service (ADACS) through the ready availability of telemedicine services | Wrong publication type |
| Mufenda | 2015 | Introducing a mobile-connected umbilical Doppler device (UmbiFlow™) into a primary care maternity setting: Does this reduce unnecessary referrals to specialised care? Results of a pilot study in kraaifontein, South Africa | Does not involve teleultrasound |
| Mwilike | 2024 | Midwife-Led Mobile Antenatal Clinic: An Innovative Approach to Improve Utilization of Services in Pwani, Tanzania | Does not involve teleultrasound |
| Naruse | 2020 | High-quality transmission of vardiotocogram and fetal information using a 5G system: pilot experiment | Wrong study design |
| Niyogi | 2025 | Role of artificial intelligence in congenital heart disease | Wrong study design |
| Olsen | 2025 | Introduction of robot-assisted obstetric ultrasound in rural Northern Norway | Duplicate |
| Oluyomi | 2023 | Introduction of a maternal-fetal medicine tele-ultrasound program in rural Alberta | Wrong publication type |
| Pardo | 2024 | 1102 Prenatal mobile self-operated home ultrasound service impact on birth outcomes | Wrong publication type |
| Pricilla | 2014 | Introduction of routine obstetric ultrasound in an urban health center: Results and benefits | Does not involve teleultrasound |
| Rabie | 2017 | Teleultrasound: A validation study | Wrong publication type |
| Rabie | 2018 | Teleultrasound: A validation study | Wrong publication type |
| Ranger | 2024 | Portable ultrasound devices for obstetric care in resource-constrained environments: mapping the landscape | Wrong publication type |
| Reddy | 2020 | Telemedicine and fetal ultrasonography in a remote Newfoundland community | Duplicate paper |
| Rizzo | 2023 | Referral ultrasound in fetal medicine: May telemedicine play a pivotal role? | Wrong study design |
| Sepulveda | 1995 | Remote fetal medicine consultation using stored ultrasound video images | Wrong study design |
| Sesay | 2025 | Assessing the Implementation and Performance of Smartphone-based Fetal Ultrasound in Sierra Leone: A Mixed-Methods Approach | Does not involve teleultrasound |
| Sharma | 2025 | Impact of a Community-Oriented Comprehensive Clinical and Ultrasound Care Model Integrating Risk Stratification, Fetal Doppler, Health Education and Low-Dose Aspirin on Preeclampsia Rates in Central India | Does not involve teleultrasound |
| Singh | 2025 | Telemedicine: The New Vanguard in Gynecological Care | Does not involve teleultrasound |
| Slimani | 2023 | Fetal biometry and amniotic fluid volume assessment end-to-end automation using deep learning | Does not involve teleultrasound |
| Smith | 2002 | Tele-ultrasound for remote areas | Wrong patient population |
| Snaith | 2017 | The introduction of a fetal ultrasound tele medicine service: Quality outcomes and family costs | Wrong publication type |
| Soffer | 2022 | Impact of a hybrid model of prenatal care on the diagnosis of fetal growth restriction | Does not involve teleultrasound |
| Spinnato | 1989 | Birth weight prediction from remote ultrasonographic examination | Duplicate paper |
| Spinnato | 1988 | Birth-weight prediction from remote ultrasound examination | Wrong indication |
| Stolz | 2025 | Effectiveness of a Web-Based Training Intervention in Teaching Emergency Physicians First-Trimester Point-of-Care Ultrasound Image Interpretation | Does not involve teleultrasound |
| Stringer | 1996 | Use of ultrasonography in the home care of high-risk childbearing women | Wrong publication type |
| Symons | 2014 | The accuracy of birth weight prediction using remote ultrasound examination | Wrong publication type |
| Thirugnanasundralingam | 2023 | Effect of telehealth-integrated antenatal care on pregnancy outcomes in Australia: an interrupted time-series analysis | Does not involve teleultrasound |
| Tonks | 2013 | An enhanced, midwifery-led ultrasound service to monitor fetal growth | Wrong publication type |
| Tromp | 2010 | Assessing inter-examiner agreement in diagnosing cardiac defects by telemedicine using four-dimensional ultrasound | Wrong publication type |
| Valderrama | 2020 | A proxy for detecting IUGR based on gestational age estimation in a Guatemalan rural population | Does not involve teleultrasound |
| Varner | 2022 | Point-of-care ultrasound for evaluation of vaginal bleeding or abdominal pain in early pregnancy Use by family physicians following focused training and certification | Does not involve teleultrasound |
| VladimirSamohvalov | 2012 | Features of the organization of first trimester prenatal screening for sparsely populated and remote areas | Wrong publication type |
| Waldum | 2025 | The HOME (home monitoring of high-risk pregnancies) study: a study protocol for an observational study of a telemedicine-assisted follow-up at home vs. hospitalization. | Does not involve teleultrasound |
| Waterstone | 1992 | Sensitivity of pocket Doppler fetal heart detectors in early pregnancy: a comparative study | Wrong publication type |
| Weiner | 2023 | Virtual prenatal care: The effect of telemedicine and home-ultrasound on maternal anxiety and attachment during pregnancy in women with a history of pregnancy loss | Wrong publication type |
| Yang | 2024 | Combined fetal echocardiographic views improved prenatal differential diagnosis between right aortic arch and double aortic arch: a multicenter research | Does not involve teleultrasound |
| Yu | 2024 | LPC-SonoNet: A Lightweight Network Based on SonoNet and Light PyramidConvolution for Fetal Ultrasound Standard Plane Detection | Does not involve teleultrasound |

ISDN, Integrated Services Digital Network; IUGR, intrauterine growth restriction; OBGYN, obstetrics and gynaecology; USG, ultrasonography.
