# Supplementary material for: Teleultrasound in obstetrics: A systematic review and meta-analysis
Source: PLoS Med. 2026 Feb 6;23(2):e1004922. doi: 10.1371/journal.pmed.1004922 (PMC12900445; doi:10.1371/journal.pmed.1004922)
Supplement: S5 Table — (DOCX) [file pmed.1004922.s005.docx]

**S5 Table:** Critical appraisal results for all studies.

| **Observational studies** | | | | | | | | | | | | | | | | | | | | | | | | | | | | | | | | | | | | | | | | | | | | | | | | | | | | | | | | | | | | | | | | | | | | | | | | | | |
| --- | --- | --- | --- | --- | --- | --- | --- | --- | --- | --- | --- | --- | --- | --- | --- | --- | --- | --- | --- | --- | --- | --- | --- | --- | --- | --- | --- | --- | --- | --- | --- | --- | --- | --- | --- | --- | --- | --- | --- | --- | --- | --- | --- | --- | --- | --- | --- | --- | --- | --- | --- | --- | --- | --- | --- | --- | --- | --- | --- | --- | --- | --- | --- | --- | --- | --- | --- | --- | --- | --- | --- | --- | --- | --- |
| **Domain 1: Generic quality standards** | | | | | | | | | | | | | | | | | | | | | | | | | | | | | | | | | | | | | | | | | | | | | | | | | | | | | | | | | | | | | | | | | | | | | | | | | | |
|  | | Adams 2018 | Adams 2020 | Adriaanse 2012 | Ahmed 2024 | Arbeille 2005 | Arbeille 2014 | Arbeille 2016 | Axelrod 2025 | | Beldjerd 2022 | | Bolin 2020 | Brown 2017 | Chan 2000 | Chan 2001 | | Cuneo 2019 | Day 2025 | Dougherty 2021 | ElGuindi 2013 | | Ferlin 2012 | Ferreira 2014 | Ferrer-Roca 2006 | | Fisk 1995 | Hadar 2022 | Hishitani 2014 | Inamura 2020 | | Inamura 2021 | Ishikawa 2023 | Jemal 2024 | Kern-Goldberger 2021 | Kozuki 2016 | Landwehr 1997 | Leighton 2019 | | Le Vance 2025 | Mabuchi 2020 | Magann 2016 | Malone 1997 | Manley 2024 | McCrossan 2011 | Meiman 2022 | | Michailidis 2001 | Neito-Calvache 2024 | Nelson 2001 | | Nir 2024 | Nores 1997 | Oelmeier 2023 | Olsen 2025 | Pardo 2024 | Pardo 2025 | | Pontones 2023 | | Rabie 2019 | Reddy 2000 | Schwartz 2021 | | Sharma 2003 | Shields 2022 | Soong 2002 | Toscano 2021 | TroyanoLuque 2013 | | Vinals 2005 | Vinals 2008 | Vinayak 2018 | Wootton 1997 |
| 1. Focused question | | 1 | 1 | 1 | 1 | 1 | 2 | 1 | 1 | | 1 | | 1 | 1 | 1 | 1 | | 1 | 1 | 1 | 1 | | 1 | 1 | 1 | | 1 | 1 | 1 | 1 | | 1 | 1 | 1 | 1 | 1 | 1 | 1 | | 1 | 1 | 1 | 1 | 1 | 1 | 1 | | 1 | 1 | 1 | | 1 | 1 | 1 | 1 | 1 | 1 | | 1 | | 1 | 1 | 1 | | 1 | 1 | 1 | 1 | 1 | | 1 | 1 | 1 | 1 |
| 1. Study design appropriate | | 1 | 1 | 2 | 1 | 1 | 1 | 1 | 1 | | 1 | | 1 | 1 | 1 | 1 | | 1 | 1 | 1 | 1 | | 1 | 1 | 1 | | 1 | 1 | 1 | 1 | | 2 | 1 | 1 | 1 | 1 | 1 | 1 | | 1 | 1 | 1 | 1 | 1 | 1 | 1 | | 1 | 1 | 1 | | 1 | 1 | 1 | 1 | 1 | 1 | | 1 | | 1 | 1 | 1 | | 1 | 1 | 1 | 1 | 1 | | 1 | 1 | 1 | 1 |
| 1. Size appropriate? | | 2 | 2 | 2 | 2 | 2 | 2 | 2 | 2 | | 2 | | 1 | 3 | 2 | 2 | | 1 | 2 | 2 | 2 | | 2 | 1 | 2 | | 3 | 1 | 2 | 2 | | 3 | 2 | 1 | 1 | 1 | 2 | 1 | | 1 | 2 | 2 | 2 | 2 | 2 | 2 | | 2 | 2 | 2 | | 2 | 2 | 2 | 2 | 2 | 1 | | 2 | | 2 | 2 | 2 | | 2 | 2 | 2 | 2 | 2 | | 2 | 2 | 2 | 2 |
| 1. Conflict of interest | | 3 | 1 | 2 | 1 | 2 | 3 | 1 | 2 | | 3 | | 1 | 1 | 3 | 3 | | 1 | 1 | 3 | 1 | | 3 | 1 | 3 | | 3 | 1 | 3 | 1 | | 1 | 1 | 1 | 1 | 1 | 3 | 1 | | 1 | 1 | 3 | 3 | 1 | 1 | 1 | | 3 | 1 | 3 | | 1 | 3 | 2 | 1 | 1 | 1 | | 1 | | 1 | 1 | 1 | | 3 | 1 | 3 | 1 | 3 | | 3 | 3 | 1 | 3 |
| 1. Ethical approval | | 1 | 1 | 1 | 1 | 1 | 1 | 2 | 1 | | 1 | | 1 | 3 | 3 | 3 | | 1 | 1 | 1 | 1 | | 1 | 1 | 3 | | 3 | 1 | 1 | 1 | | 3 | 1 | 1 | 1 | 2 | 1 | 1 | | 1 | 1 | 1 | 1 | 1 | 1 | 1 | | 3 | 1 | 1 | | 1 | 1 | 1 | 1 | 1 | 1 | | 1 | | 1 | 3 | 4 | | 1 | 1 | 3 | 1 | 1 | | 3 | 3 | 1 | 3 |
| 1. Informed consent | | 1 | 1 | 1 | 1 | 1 | 1 | 2 | 1 | | 1 | | 1 | 3 | 3 | 3 | | 2 | 1 | 1 | 2 | | 3 | 1 | 3 | | 2 | 1 | 1 | 1 | | 2 | 1 | 1 | 2 | 1 | 2 | 2 | | 1 | 1 | 3 | 1 | 1 | 1 | 2 | | 3 | 1 | 3 | | 1 | 1 | 1 | 1 | 1 | 1 | | 1 | | 2 | 3 | 4 | | 1 | 1 | 3 | 1 | 1 | | 3 | 1 | 1 | 3 |
| 1. Replicable methods | | 1 | 1 | 3 | 1 | 1 | 3 | 3 | 1 | | 1 | | 2 | 1 | 1 | 1 | | 1 | 1 | 1 | 3 | | 1 | 1 | 1 | | 1 | 1 | 2 | 2 | | 3 | 2 | 2 | 3 | 1 | 1 | 2 | | 1 | 1 | 2 | 1 | 1 | 3 | 1 | | 3 | 1 | 1 | | 3 | 1 | 2 | 1 | 1 | 1 | | 1 | | 3 | 3 | 1 | | 1 | 1 | 1 | 1 | 1 | | 1 | 1 | 1 | 1 |
| 1. Selective reporting | | 1 | 1 | 1 | 1 | 1 | 2 | 1 | 1 | | 1 | | 1 | 1 | 1 | 1 | | 1 | 1 | 1 | 2 | | 1 | 1 | 1 | | 1 | 1 | 1 | 1 | | 1 | 1 | 1 | 1 | 1 | 1 | 1 | | 1 | 1 | 1 | 1 | 1 | 1 | 1 | | 1 | 1 | 1 | | 1 | 1 | 1 | 1 | 1 | 1 | | 1 | | 1 | 2 | 1 | | 1 | 1 | 1 | 1 | 1 | | 1 | 1 | 2 | 1 |
| 1. Study limitations | | 1 | 1 | 1 | 1 | 3 | 1 | 1 | 1 | | 1 | | 1 | 1 | 3 | 1 | | 3 | 1 | 1 | 3 | | 1 | 1 | 1 | | 2 | 1 | 1 | 1 | | 3 | 1 | 1 | 1 | 1 | 1 | 1 | | 1 | 1 | 1 | 1 | 1 | 1 | 1 | | 1 | 1 | 1 | | 1 | 1 | 1 | 1 | 1 | 1 | | 1 | | 1 | 3 | 2 | | 1 | 1 | 1 | 1 | 2 | | 1 | 1 | 3 | 1 |
| 1. Generalisability reported | | 2 | 1 | 3 | 1 | 1 | 3 | 2 | 1 | | 1 | | 1 | 2 | 2 | 1 | | 2 | 1 | 1 | 3 | | 2 | 1 | 1 | | 1 | 2 | 2 | 2 | | 1 | 2 | 1 | 1 | 1 | 1 | 1 | | 1 | 3 | 1 | 1 | 1 | 1 | 1 | | 2 | 1 | 1 | | 1 | 2 | 1 | 1 | 1 | 1 | | 1 | | 1 | 1 | 1 | | 1 | 1 | 1 | 1 | 2 | | 2 | 1 | 1 | 2 |
| **Domain 2: Patient/Participant selection** | | | | | | | | | | | | | | | | | | | | | | | | | | | | | | | | | | | | | | | | | | | | | | | | | | | | | | | | | | | | | | | | | | | | | | | | | | |
|  | | Adams 2018 | Adams 2020 | Adriaanse 2012 | Ahmed 2024 | Arbeille 2005 | Arbeille 2014 | Arbeille 2016 | Axelrod 2025 | | Beldjerd 2022 | | Bolin 2020 | Brown 2017 | Chan 2000 | Chan 2001 | | Cuneo 2019 | Day 2025 | Dougherty 2021 | ElGuindi 2013 | | Ferlin 2012 | Ferreira 2014 | Ferrer-Roca 2006 | | Fisk 1995 | Hadar 2022 | Hishitani 2014 | Inamura 2020 | | Inamura 2021 | Ishikawa 2023 | Jemal 2024 | Kern-Goldberger 2021 | Kozuki 2016 | Landwehr 1997 | Leighton 2019 | | Le Vance 2025 | Mabuchi 2020 | Magann 2016 | Malone 1997 | Manley 2024 | McCrossan 2011 | Meiman 2022 | | Michailidis 2001 | Neito-Calvache 2024 | Nelson 2001 | | Nir 2024 | Nores 1997 | Oelmeier 2023 | Olsen 2025 | Pardo 2024 | Pardo 2025 | | Pontones 2023 | | Rabie 2019 | Reddy 2000 | Schwartz 2021 | | Sharma 2003 | Shields 2022 | Soong 2002 | Toscano 2021 | TroyanoLuque 2013 | | Vinals 2005 | Vinals 2008 | Vinayak 2018 | Wootton 1997 |
| 1. Simulation representative | | 4 | 4 | 4 | 4 | 4 | 4 | 4 | 4 | | 4 | | 4 | 4 | 4 | 4 | | 4 | 4 | 4 | 4 | | 4 | 4 | 4 | | 4 | 4 | 4 | 4 | | 4 | 4 | 4 | 4 | 4 | 4 | 4 | | 4 | 4 | 4 | 4 | 4 | 4 | 4 | | 4 | 4 | 4 | | 4 | 4 | 4 | 4 | 4 | 4 | | 4 | | 4 | 4 | 4 | | 4 | 4 | 4 | 4 | 4 | | 4 | 4 | 4 | 4 |
| 1. Level of expertise described | | 1 | 1 | 1 | 1 | 3 | 3 | 1 | 3 | | 1 | | 1 | 1 | 3 | 3 | | 1 | 1 | 1 | 3 | | 3 | 1 | 3 | | 3 | 3 | 3 | 3 | | 3 | 3 | 2 | 2 | 1 | 3 | 1 | | 3 | 3 | 1 | 1 | 1 | 3 | 1 | | 3 | 1 | 1 | | 3 | 3 | 1 | 3 | 1 | 1 | | 3 | | 3 | 3 | 2 | | 2 | 2 | 3 | 3 | 2 | | 1 | 1 | 1 | 1 |
| 1. Methods of participation | | 2 | 2 | 2 | 2 | 2 | 2 | 2 | 1 | | 1 | | 1 | 2 | 2 | 2 | | 2 | 1 | 2 | 1 | | 1 | 1 | 3 | | 3 | 1 | 3 | 2 | | 4 | 1 | 1 | 1 | 1 | 2 | 1 | | 1 | 1 | 1 | 1 | 2 | 1 | 1 | | 3 | 1 | 3 | | 1 | 1 | 1 | 2 | 1 | 1 | | 1 | | 1 | 3 | 1 | | 1 | 2 | 2 | 1 | 1 | | 3 | 1 | 1 | 3 |
| 1. Inappropriate exclusions | | 2 | 2 | 2 | 2 | 2 | 3 | 2 | 1 | | 4 | | 1 | 2 | 2 | 3 | | 3 | 1 | 3 | 3 | | 1 | 1 | 3 | | 3 | 1 | 3 | 3 | | 4 | 1 | 1 | 1 | 1 | 3 | 1 | | 1 | 1 | 1 | 2 | 3 | 2 | 3 | | 3 | 1 | 3 | | 1 | 1 | 1 | 2 | 1 | 1 | | 1 | | 1 | 3 | 3 | | 3 | 2 | 3 | 1 | 2 | | 3 | 3 | 2 | 3 |
| 1. Participant selection | | 2 | 3 | 3 | 2 | 2 | 3 | 2 | 1 | | 1 | | 1 | 2 | 2 | 3 | | 2 | 1 | 2 | 2 | | 1 | 1 | 3 | | 3 | 1 | 2 | 2 | | 2 | 2 | 1 | 1 | 1 | 2 | 1 | | 1 | 1 | 1 | 1 | 2 | 1 | 1 | | 3 | 1 | 2 | | 1 | 1 | 1 | 3 | 1 | 1 | | 1 | | 1 | 2 | 2 | | 2 | 1 | 2 | 1 | 2 | | 2 | 2 | 1 | 2 |
| **Domain 3: Index test(s)** | | | | | | | | | | | | | | | | | | | | | | | | | | | | | | | | | | | | | | | | | | | | | | | | | | | | | | | | | | | | | | | | | | | | | | | | | | |
|  | | Adams 2018 | Adams 2020 | Adriaanse 2012 | Ahmed 2024 | Arbeille 2005 | Arbeille 2014 | Arbeille 2016 | Axelrod 2025 | | Beldjerd 2022 | | Bolin 2020 | Brown 2017 | Chan 2000 | Chan 2001 | | Cuneo 2019 | Day 2025 | Dougherty 2021 | ElGuindi 2013 | | Ferlin 2012 | Ferreira 2014 | Ferrer-Roca 2006 | | Fisk 1995 | Hadar 2022 | Hishitani 2014 | Inamura 2020 | | Inamura 2021 | Ishikawa 2023 | Jemal 2024 | Kern-Goldberger 2021 | Kozuki 2016 | Landwehr 1997 | Leighton 2019 | | Le Vance 2025 | Mabuchi 2020 | Magann 2016 | Malone 1997 | Manley 2024 | McCrossan 2011 | Meiman 2022 | | Michailidis 2001 | Neito-Calvache 2024 | Nelson 2001 | | Nir 2024 | Nores 1997 | Oelmeier 2023 | Olsen 2025 | Pardo 2024 | Pardo 2025 | | Pontones 2023 | | Rabie 2019 | Reddy 2000 | Schwartz 2021 | | Sharma 2003 | Shields 2022 | Soong 2002 | Toscano 2021 | TroyanoLuque 2013 | | Vinals 2005 | Vinals 2008 | Vinayak 2018 | Wootton 1997 |
| 1. Reviewers blinded | | 1 | 3 | 1 | 3 | 2 | 2 | 3 | 3 | | 3 | | 2 | 3 | 3 | 2 | | 4 | 3 | 1 | 3 | | 3 | 1 | 3 | | 1 | 2 | 2 | 3 | | 3 | 2 | 3 | 3 | 2 | 1 | 2 | | 3 | 3 | 3 | 1 | 3 | 3 | 3 | | 3 | 1 | 1 | | 3 | 1 | 3 | 3 | 1 | 3 | | 3 | | 4 | 1 | 3 | | 3 | 3 | 1 | 1 | 3 | | 3 | 3 | 3 | 1 |
| 1. Outcome measures | | 1 | 1 | 1 | 1 | 1 | 3 | 2 | 1 | | 1 | | 1 | 1 | 1 | 1 | | 1 | 1 | 1 | 1 | | 1 | 1 | 1 | | 1 | 1 | 1 | 1 | | 4 | 1 | 1 | 1 | 1 | 1 | 1 | | 1 | 1 | 1 | 1 | 1 | 1 | 1 | | 1 | 1 | 1 | | 1 | 1 | 1 | 1 | 1 | 1 | | 1 | | 1 | 1 | 1 | | 1 | 1 | 1 | 1 | 1 | | 1 | 1 | 1 | 1 |
| 1. Subjective outcome | | 1 | 3 | 4 | 1 | 4 | 4 | 4 | 1 | | 4 | | 4 | 4 | 1 | 1 | | 3 | 4 | 4 | 4 | | 1 | 1 | 4 | | 4 | 1 | 4 | 4 | | 4 | 2 | 1 | 1 | 4 | 4 | 4 | | 1 | 4 | 4 | 4 | 4 | 1 | 4 | | 4 | 3 | 1 | | 1 | 4 | 1 | 1 | 3 | 3 | | 1 | | 4 | 4 | 4 | | 1 | 1 | 1 | 1 | 4 | | 4 | 4 | 4 | 1 |
| 1. Statistical tests | | 1 | 1 | 1 | 4 | 1 | 3 | 3 | 1 | | 1 | | 1 | 1 | 1 | 1 | | 1 | 1 | 1 | 1 | | 1 | 1 | 1 | | 4 | 1 | 1 | 1 | | 4 | 4 | 1 | 1 | 1 | 1 | 1 | | 1 | 1 | 1 | 1 | 1 | 1 | 1 | | 1 | 1 | 3 | | 1 | 1 | 1 | 1 | 1 | 1 | | 1 | | 1 | 1 | 1 | | 1 | 1 | 4 | 1 | 1 | | 1 | 1 | 2 | 1 |
| 1. Confidence intervals | | 1 | 3 | 4 | 4 | 3 | 4 | 4 | 3 | | 4 | | 3 | 3 | 4 | 3 | | 4 | 1 | 1 | 4 | | 4 | 1 | 4 | | 4 | 1 | 4 | 3 | | 4 | 4 | 4 | 4 | 1 | 3 | 1 | | 3 | 4 | 1 | 3 | 4 | 3 | 4 | | 1 | 3 | 4 | | 4 | 1 | 4 | 1 | 3 | 1 | | 4 | | 1 | 4 | 4 | | 4 | 1 | 4 | 1 | 4 | | 4 | 4 | 4 | 4 |
| 1. Introduction of bias | | 1 | 3 | 1 | 1 | 3 | 3 | 3 | 2 | | 2 | | 1 | 1 | 1 | 2 | | 1 | 1 | 1 | 3 | | 3 | 1 | 2 | | 1 | 1 | 2 | 3 | | 2 | 2 | 2 | 2 | 2 | 1 | 2 | | 1 | 1 | 2 | 1 | 2 | 2 | 2 | | 3 | 1 | 2 | | 1 | 1 | 2 | 2 | 1 | 1 | | 3 | | 1 | 1 | 2 | | 2 | 1 | 1 | 1 | 3 | | 2 | 2 | 2 | 1 |
| **Domain 4: Reference standard** | | | | | | | | | | | | | | | | | | | | | | | | | | | | | | | | | | | | | | | | | | | | | | | | | | | | | | | | | | | | | | | | | | | | | | | | | | |
|  | | Adams 2018 | Adams 2020 | Adriaanse 2012 | Ahmed 2024 | Arbeille 2005 | Arbeille 2014 | Arbeille 2016 | Axelrod 2025 | | Beldjerd 2022 | | Bolin 2020 | Brown 2017 | Chan 2000 | Chan 2001 | | Cuneo 2019 | Day 2025 | Dougherty 2021 | ElGuindi 2013 | | Ferlin 2012 | Ferreira 2014 | Ferrer-Roca 2006 | | Fisk 1995 | Hadar 2022 | Hishitani 2014 | Inamura 2020 | | Inamura 2021 | Ishikawa 2023 | Jemal 2024 | Kern-Goldberger 2021 | Kozuki 2016 | Landwehr 1997 | Leighton 2019 | | Le Vance 2025 | Mabuchi 2020 | Magann 2016 | Malone 1997 | Manley 2024 | McCrossan 2011 | Meiman 2022 | | Michailidis 2001 | Neito-Calvache 2024 | Nelson 2001 | | Nir 2024 | Nores 1997 | Oelmeier 2023 | Olsen 2025 | Pardo 2024 | Pardo 2025 | | Pontones 2023 | | Rabie 2019 | Reddy 2000 | Schwartz 2021 | | Sharma 2003 | Shields 2022 | Soong 2002 | Toscano 2021 | TroyanoLuque 2013 | | Vinals 2005 | Vinals 2008 | Vinayak 2018 | Wootton 1997 |
| 1. Reference standard | | 1 | 4 | 1 | 3 | 1 | 3 | 3 | 3 | | 3 | | 1 | 1 | 3 | 3 | | 1 | 1 | 1 | 1 | | 1 | 1 | 1 | | 4 | 3 | 3 | 1 | | 3 | 3 | 1 | 1 | 3 | 1 | 1 | | 3 | 1 | 1 | 1 | 1 | 1 | 3 | | 3 | 1 | 1 | | 3 | 1 | 4 | 1 | 1 | 3 | | 4 | | 1 | 1 | 1 | | 1 | 1 | 3 | 1 | 1 | | 3 | 3 | 3 | 3 |
| 1. Reference standard appropriate | | 1 | 4 | 1 | 4 | 1 | 4 | 4 | 4 | | 4 | | 1 | 1 | 4 | 4 | | 1 | 1 | 1 | 1 | | 1 | 1 | 1 | | 4 | 4 | 4 | 1 | | 4 | 4 | 1 | 1 | 4 | 1 | 1 | | 4 | 1 | 1 | 3 | 2 | 1 | 4 | | 4 | 1 | 1 | | 4 | 2 | 4 | 1 | 1 | 4 | | 4 | | 1 | 1 | 1 | | 1 | 1 | 4 | 1 | 1 | | 4 | 4 | 4 | 4 |
| 1. Blind interpretation | | 1 | 4 | 1 | 4 | 2 | 4 | 4 | 4 | | 4 | | 2 | 2 | 4 | 4 | | 2 | 2 | 1 | 3 | | 3 | 1 | 2 | | 4 | 4 | 4 | 2 | | 4 | 4 | 2 | 2 | 4 | 1 | 4 | | 1 | 3 | 2 | 1 | 2 | 3 | 4 | | 4 | 2 | 1 | | 4 | 1 | 4 | 1 | 1 | 4 | | 4 | | 2 | 1 | 3 | | 4 | 2 | 4 | 1 | 3 | | 4 | 4 | 4 | 4 |
| 1. Introduction of bias | | 1 | 4 | 1 | 4 | 2 | 2 | 2 | 4 | | 4 | | 1 | 1 | 4 | 4 | | 1 | 2 | 1 | 3 | | 1 | 1 | 2 | | 1 | 1 | 1 | 2 | | 4 | 1 | 2 | 2 | 4 | 1 | 1 | | 1 | 1 | 1 | 1 | 2 | 2 | 1 | | 1 | 1 | 1 | | 1 | 1 | 4 | 1 | 1 | 4 | | 4 | | 2 | 1 | 1 | | 1 | 1 | 4 | 1 | 2 | | 4 | 1 | 4 | 4 |
| **Domain 5: Flow and timing** | | | | | | | | | | | | | | | | | | | | | | | | | | | | | | | | | | | | | | | | | | | | | | | | | | | | | | | | | | | | | | | | | | | | | | | | | | |
|  | | Adams 2018 | Adams 2020 | Adriaanse 2012 | Ahmed 2024 | Arbeille 2005 | Arbeille 2014 | Arbeille 2016 | Axelrod 2025 | | Beldjerd 2022 | | Bolin 2020 | Brown 2017 | Chan 2000 | Chan 2001 | | Cuneo 2019 | Day 2025 | Dougherty 2021 | ElGuindi 2013 | | Ferlin 2012 | Ferreira 2014 | Ferrer-Roca 2006 | | Fisk 1995 | Hadar 2022 | Hishitani 2014 | Inamura 2020 | | Inamura 2021 | Ishikawa 2023 | Jemal 2024 | Kern-Goldberger 2021 | Kozuki 2016 | Landwehr 1997 | Leighton 2019 | | Le Vance 2025 | Mabuchi 2020 | Magann 2016 | Malone 1997 | Manley 2024 | McCrossan 2011 | Meiman 2022 | | Michailidis 2001 | Neito-Calvache 2024 | Nelson 2001 | | Nir 2024 | Nores 1997 | Oelmeier 2023 | Olsen 2025 | Pardo 2024 | Pardo 2025 | | Pontones 2023 | | Rabie 2019 | Reddy 2000 | Schwartz 2021 | | Sharma 2003 | Shields 2022 | Soong 2002 | Toscano 2021 | TroyanoLuque 2013 | | Vinals 2005 | Vinals 2008 | Vinayak 2018 | Wootton 1997 |
| 1. Appropriate interval | | 1 | 4 | 2 | 4 | 2 | 4 | 4 | 4 | | 4 | | 2 | 2 | 4 | 4 | | 1 | 2 | 1 | 2 | | 2 | 2 | 2 | | 4 | 4 | 4 | 1 | | 4 | 4 | 2 | 2 | 4 | 1 | 4 | | 4 | 1 | 1 | 1 | 2 | 2 | 4 | | 4 | 1 | 1 | | 4 | 1 | 4 | 1 | 1 | 4 | | 4 | | 1 | 3 | 1 | | 4 | 4 | 4 | 1 | 2 | | 4 | 4 | 4 | 4 |
| 1. All receive a reference standard | | 1 | 4 | 1 | 4 | 1 | 4 | 4 | 4 | | 4 | | 1 | 3 | 4 | 4 | | 3 | 1 | 1 | 1 | | 1 | 1 | 1 | | 4 | 4 | 4 | 1 | | 4 | 4 | 1 | 1 | 4 | 1 | 3 | | 4 | 3 | 1 | 1 | 2 | 1 | 4 | | 4 | 1 | 1 | | 4 | 1 | 4 | 1 | 1 | 4 | | 4 | | 1 | 1 | 1 | | 3 | 4 | 4 | 1 | 1 | | 4 | 4 | 4 | 4 |
| 1. Same reference standard | | 1 | 4 | 1 | 4 | 1 | 4 | 4 | 4 | | 4 | | 1 | 3 | 4 | 4 | | 1 | 1 | 1 | 1 | | 1 | 1 | 1 | | 4 | 4 | 4 | 1 | | 4 | 4 | 1 | 1 | 4 | 1 | 3 | | 4 | 1 | 2 | 1 | 2 | 1 | 4 | | 4 | 1 | 1 | | 4 | 1 | 4 | 1 | 1 | 4 | | 4 | | 2 | 1 | 2 | | 3 | 4 | 4 | 1 | 1 | | 4 | 4 | 4 | 4 |
| 1. Complete inclusion | | 1 | 2 | 1 | 1 | 1 | 1 | 2 | 4 | | 1 | | 1 | 1 | 1 | 2 | | 1 | 1 | 3 | 1 | | 2 | 1 | 2 | | 2 | 1 | 2 | 1 | | 4 | 3 | 1 | 1 | 1 | 2 | 1 | | 1 | 1 | 2 | 1 | 2 | 1 | 1 | | 1 | 1 | 1 | | 1 | 1 | 1 | 1 | 1 | 4 | | 4 | | 1 | 1 | 1 | | 1 | 1 | 1 | 1 | 1 | | 1 | 1 | 1 | 1 |
| 1. Introduction of bias | | 1 | 2 | 2 | 1 | 2 | 2 | 2 | 1 | | 1 | | 1 | 3 | 1 | 2 | | 1 | 1 | 2 | 1 | | 2 | 1 | 1 | | 1 | 1 | 1 | 1 | | 1 | 2 | 1 | 1 | 1 | 1 | 1 | | 1 | 1 | 1 | 1 | 2 | 1 | 1 | | 1 | 1 | 1 | | 1 | 1 | 1 | 1 | 1 | 1 | | 1 | | 2 | 1 | 1 | | 1 | 1 | 1 | 1 | 1 | | 1 | 1 | 1 | 1 |
| **Domain 6: Telemedicine/ Feasibility specific concerns** | | | | | | | | | | | | | | | | | | | | | | | | | | | | | | | | | | | | | | | | | | | | | | | | | | | | | | | | | | | | | | | | | | | | | | | | | | |
|  | | Adams 2018 | Adams 2020 | Adriaanse 2012 | Ahmed 2024 | Arbeille 2005 | Arbeille 2014 | Arbeille 2016 | Axelrod 2025 | | Beldjerd 2022 | | Bolin 2020 | Brown 2017 | Chan 2000 | Chan 2001 | | Cuneo 2019 | Day 2025 | Dougherty 2021 | ElGuindi 2013 | | Ferlin 2012 | Ferreira 2014 | Ferrer-Roca 2006 | | Fisk 1995 | Hadar 2022 | Hishitani 2014 | Inamura 2020 | | Inamura 2021 | Ishikawa 2023 | Jemal 2024 | Kern-Goldberger 2021 | Kozuki 2016 | Landwehr 1997 | Leighton 2019 | | Le Vance 2025 | Mabuchi 2020 | Magann 2016 | Malone 1997 | Manley 2024 | McCrossan 2011 | Meiman 2022 | | Michailidis 2001 | Neito-Calvache 2024 | Nelson 2001 | | Nir 2024 | Nores 1997 | Oelmeier 2023 | Olsen 2025 | Pardo 2024 | Pardo 2025 | | Pontones 2023 | | Rabie 2019 | Reddy 2000 | Schwartz 2021 | | Sharma 2003 | Shields 2022 | Soong 2002 | Toscano 2021 | TroyanoLuque 2013 | | Vinals 2005 | Vinals 2008 | Vinayak 2018 | Wootton 1997 |
| 1. Security measures used | | 3 | 3 | 3 | 3 | 3 | 3 | 3 | 3 | | 1 | | 3 | 1 | 3 | 3 | | 3 | 3 | 3 | 3 | | 3 | 1 | 3 | | 3 | 1 | 1 | 1 | | 1 | 1 | 3 | 3 | 3 | 3 | 1 | | 1 | 1 | 3 | 3 | 4 | 3 | 1 | | 3 | 1 | 3 | | 3 | 3 | 3 | 2 | 3 | 3 | | 3 | | 3 | 3 | 3 | | 3 | 3 | 1 | 1 | 3 | | 2 | 3 | 3 | 3 |
| 1. Receiving or sending environments typical | | 1 | 1 | 2 | 1 | 1 | 1 | 1 | 1 | | 1 | | 1 | 1 | 1 | 1 | | 1 | 1 | 1 | 1 | | 1 | 1 | 1 | | 1 | 1 | 1 | 2 | | 1 | 1 | 1 | 2 | 1 | 1 | 2 | | 1 | 1 | 1 | 1 | 1 | 1 | 1 | | 1 | 1 | 1 | | 1 | 1 | 1 | 1 | 1 | 1 | | 1 | | 2 | 1 | 1 | | 1 | 1 | 1 | 1 | 1 | | 1 | 1 | 1 | 1 |
| 1. Technical standards | | 3 | 1 | 2 | 3 | 1 | 1 | 1 | 3 | | 3 | | 1 | 1 | 1 | 1 | | 1 | 3 | 3 | 3 | | 1 | 1 | 1 | | 1 | 2 | 1 | 3 | | 3 | 3 | 1 | 3 | 3 | 1 | 3 | | 2 | 1 | 3 | 1 | 3 | 3 | 3 | | 3 | 3 | 1 | | 3 | 1 | 3 | 3 | 1 | 1 | | 1 | | 3 | 3 | 3 | | 1 | 3 | 1 | 1 | 1 | | 1 | 2 | 3 | 1 |
| 1. Level of cost | | 3 | 3 | 3 | 1 | 3 | 3 | 3 | 3 | | 1 | | 3 | 3 | 3 | 1 | | 1 | 3 | 3 | 3 | | 1 | 3 | 3 | | 3 | 3 | 1 | 3 | | 3 | 3 | 3 | 3 | 1 | 3 | 1 | | 3 | 3 | 3 | 3 | 3 | 1 | 3 | | 3 | 3 | 3 | | 3 | 3 | 3 | 1 | 3 | 3 | | 3 | | 3 | 3 | 3 | | 3 | 1 | 1 | 3 | 3 | | 3 | 3 | 3 | 3 |
| 1. Technical barriers | | 2 | 2 | 3 | 1 | 1 | 1 | 1 | 2 | | 3 | | 3 | 1 | 1 | 1 | | 2 | 3 | 3 | 3 | | 2 | 4 | 1 | | 1 | 3 | 3 | 2 | | 3 | 1 | 1 | 2 | 2 | 3 | 3 | | 3 | 3 | 3 | 1 | 3 | 3 | 2 | | 2 | 2 | 2 | | 3 | 2 | 1 | 3 | 2 | 3 | | 3 | | 3 | 3 | 1 | | 1 | 1 | 1 | 1 | 2 | | 1 | 3 | 2 | 3 |
| **Domain 7: Concerns regarding applicability** | | | | | | | | | | | | | | | | | | | | | | | | | | | | | | | | | | | | | | | | | | | | | | | | | | | | | | | | | | | | | | | | | | | | | | | | | | |
|  | | Adams 2018 | Adams 2020 | Adriaanse 2012 | Ahmed 2024 | Arbeille 2005 | Arbeille 2014 | Arbeille 2016 | Axelrod 2025 | | Beldjerd 2022 | | Bolin 2020 | Brown 2017 | Chan 2000 | Chan 2001 | | Cuneo 2019 | Day 2025 | Dougherty 2021 | ElGuindi 2013 | | Ferlin 2012 | Ferreira 2014 | Ferrer-Roca 2006 | | Fisk 1995 | Hadar 2022 | Hishitani 2014 | Inamura 2020 | | Inamura 2021 | Ishikawa 2023 | Jemal 2024 | Kern-Goldberger 2021 | Kozuki 2016 | Landwehr 1997 | Leighton 2019 | | Le Vance 2025 | Mabuchi 2020 | Magann 2016 | Malone 1997 | Manley 2024 | McCrossan 2011 | Meiman 2022 | | Michailidis 2001 | Neito-Calvache 2024 | Nelson 2001 | | Nir 2024 | Nores 1997 | Oelmeier 2023 | Olsen 2025 | Pardo 2024 | Pardo 2025 | | Pontones 2023 | | Rabie 2019 | Reddy 2000 | Schwartz 2021 | | Sharma 2003 | Shields 2022 | Soong 2002 | Toscano 2021 | TroyanoLuque 2013 | | Vinals 2005 | Vinals 2008 | Vinayak 2018 | Wootton 1997 |
| 1. Reference standard applicable | | 1 | 4 | 1 | 4 | 1 | 4 | 4 | 4 | | 4 | | 1 | 2 | 4 | 4 | | 1 | 1 | 1 | 1 | | 1 | 1 | 1 | | 4 | 4 | 4 | 1 | | 4 | 4 | 1 | 1 | 4 | 1 | 1 | | 4 | 1 | 1 | 1 | 1 | 1 | 4 | | 4 | 1 | 1 | | 4 | 1 | 4 | 1 | 1 | 4 | | 4 | | 4 | 1 | 1 | | 4 | 1 | 4 | 1 | 1 | | 4 | 1 | 4 | 4 |
| 1. Included patients applicable | | 1 | 1 | 1 | 1 | 1 | 1 | 2 | 1 | | 1 | | 1 | 1 | 1 | 2 | | 1 | 1 | 1 | 1 | | 1 | 1 | 2 | | 2 | 1 | 1 | 1 | | 1 | 1 | 1 | 1 | 1 | 1 | 1 | | 1 | 1 | 1 | 1 | 1 | 1 | 1 | | 1 | 1 | 2 | | 1 | 1 | 1 | 1 | 1 | 1 | | 1 | | 1 | 1 | 1 | | 1 | 1 | 1 | 1 | 1 | | 1 | 1 | 1 | 1 |
| 1. Conduct of ultrasound applicable | | 1 | 1 | 1 | 1 | 1 | 1 | 1 | 1 | | 1 | | 1 | 1 | 1 | 2 | | 1 | 1 | 1 | 2 | | 1 | 1 | 1 | | 1 | 1 | 1 | 1 | | 1 | 1 | 1 | 1 | 1 | 1 | 1 | | 1 | 1 | 1 | 1 | 1 | 1 | 1 | | 1 | 1 | 1 | | 1 | 1 | 1 | 1 | 1 | 1 | | 1 | | 1 | 1 | 1 | | 1 | 1 | 1 | 1 | 1 | | 1 | 1 | 1 | 1 |
| 1. Mode of telecommunications applicable | | 1 | 1 | 1 | 1 | 1 | 1 | 1 | 1 | | 1 | | 1 | 1 | 1 | 1 | | 1 | 1 | 1 | 2 | | 1 | 1 | 1 | | 1 | 1 | 1 | 1 | | 1 | 1 | 1 | 1 | 1 | 1 | 1 | | 1 | 1 | 1 | 1 | 1 | 1 | 1 | | 1 | 1 | 1 | | 1 | 1 | 1 | 1 | 1 | 1 | | 1 | | 1 | 1 | 1 | | 1 | 1 | 1 | 1 | 1 | | 1 | 1 | 1 | 1 |
|  | | | | | | | | | | | | | | | | | | | | | | | | | | | | | | | | | | | | | | | | | | | | | | | | | | | | | | | | | | | | | | | | | | | | | | | | | | |
| **Qualitative studies** | | | | | | | | | | | | | | | | | | | | | | | | | | | | | | | | | | | | | | **Economic evaluation studies** | | | | | | | | | | | | | | | | | | | | | | | | | | | | | | | | | | | | |
|  |  | | | | | | | | | Bidmead 2020 | | | | | | | Hishitani 2014 | | | | | Kumar 2023 | | | | McCrossan 2012 | | | | | Smith 2021 | | | | | | | |  | | | | | | | | | | | | Beldjerd 2023 | | | | | | | Dowie 2008 | | | | | | Malone 1998 | | | | | | Mistry 2013 | | | | |
|  | Philosophical congruity | | | | | | | | | 1 | | | | | | | 2 | | | | | 1 | | | | 2 | | | | | 1 | | | | | | | | 1. Well-defined question | | | | | | | | | | | | 1 | | | | | | | 1 | | | | | | 1 | | | | | | 1 | | | | |
|  | Methodology and research question congruity | | | | | | | | | 1 | | | | | | | 1 | | | | | 1 | | | | 1 | | | | | 1 | | | | | | | | 1. Comprehensive description | | | | | | | | | | | | 1 | | | | | | | 1 | | | | | | 1 | | | | | | 2 | | | | |
|  | Methodology and data collection congruity | | | | | | | | | 1 | | | | | | | 1 | | | | | 1 | | | | 3 | | | | | 1 | | | | | | | | 1. Costs and outcomes for alternatives | | | | | | | | | | | | 1 | | | | | | | 1 | | | | | | 1 | | | | | | 1 | | | | |
|  | Methodology and data analysis congruity | | | | | | | | | 1 | | | | | | | 1 | | | | | 1 | | | | 1 | | | | | 1 | | | | | | | | 1. Clinical effectiveness | | | | | | | | | | | | 2 | | | | | | | 2 | | | | | | 2 | | | | | | 2 | | | | |
|  | Methodology and result interpretation congruity | | | | | | | | | 1 | | | | | | | 1 | | | | | 1 | | | | 1 | | | | | 1 | | | | | | | | 1. Costs measured accurately | | | | | | | | | | | | 1 | | | | | | | 1 | | | | | | 1 | | | | | | 1 | | | | |
|  | Research culture statement | | | | | | | | | 3 | | | | | | | 3 | | | | | 3 | | | | 3 | | | | | 3 | | | | | | | | 1. Costs valued credibly | | | | | | | | | | | | 1 | | | | | | | 1 | | | | | | 1 | | | | | | 1 | | | | |
|  | Research influence | | | | | | | | | 2 | | | | | | | 2 | | | | | 1 | | | | 3 | | | | | 1 | | | | | | | | 1. Adjusted for differential timing | | | | | | | | | | | | 3 | | | | | | | 1 | | | | | | 2 | | | | | | 3 | | | | |
|  | Participant voices heard | | | | | | | | | 1 | | | | | | | 2 | | | | | 1 | | | | 1 | | | | | 2 | | | | | | | | 1. Incremental analysis of costs | | | | | | | | | | | | 1 | | | | | | | 1 | | | | | | 3 | | | | | | 1 | | | | |
|  | Research ethics | | | | | | | | | 1 | | | | | | | 3 | | | | | 1 | | | | 1 | | | | | 1 | | | | | | | | 1. Sensitivity analysis | | | | | | | | | | | | 1 | | | | | | | 1 | | | | | | 3 | | | | | | 1 | | | | |
|  | Flow of research | | | | | | | | | 1 | | | | | | | 1 | | | | | 1 | | | | 1 | | | | | 1 | | | | | | | | 1. All issues of concern to users | | | | | | | | | | | | 1 | | | | | | | 1 | | | | | | 2 | | | | | | 1 | | | | |
|  | | | | | | | | | | | | | | | | | | | | | | | | | | | | | | | | | | | | | | | 1. Generalisable results | | | | | | | | | | | | 1 | | | | | | | 2 | | | | | | 1 | | | | | | 1 | | | | |
| **Randomised controlled studies** | | | | | | | | | | | | | | | | | | | | | | | | | | | | | | | | | | | | | | | | | | | | | | | | | | | | | | | | | | | | | | | | | | | | | | | | | | |
|  | | | | | | | | | | | | Domain 1: Randomisation process | | | | | | | | | | | | | | Domain 2: Deviations from the intended interventions | | | | | | | | | Domain 3: Missing outcome data | | | | | | | | | | | | Domain 4: Measurement of the outcome | | | | | | | | | | | | | Domain 5: Selection of the reported  result | | | | | | | | | | | | | | |
| Mor 2024 | | | | | | | | | | | | Low risk | | | | | | | | | | | | | | Some concerns | | | | | | | | | Low risk | | | | | | | | | | | | Some concerns | | | | | | | | | | | | | Low risk | | | | | | | | | | | | | | |
| Whittington 2022 | | | | | | | | | | | | Low risk | | | | | | | | | | | | | | Low risk | | | | | | | | | Low risk | | | | | | | | | | | | Low risk | | | | | | | | | | | | | Low risk | | | | | | | | | | | | | | |
