# Supplementary material for: Teleultrasound in obstetrics: A systematic review and meta-analysis
Source: PLoS Med. 2026 Feb 6;23(2):e1004922. doi: 10.1371/journal.pmed.1004922 (PMC12900445; doi:10.1371/journal.pmed.1004922)
Supplement: S6 Table — (DOCX) [file pmed.1004922.s006.docx]

**S6 Table:** Description of study methodology.

| **Feasibility studies** | | | | | | |
| --- | --- | --- | --- | --- | --- | --- |
| Title/year | Description of methodology | Study details | Operating environment | Receiving environment | Technical details | Summary of findings |
| Adams 2020 (24) | A telerobotic ultrasound system was used to perform obstetrical ultrasound exams remotely in a remote community without regular access to obstetrical ultrasound. Using a telerobotic ultrasound system, a sonographer 605km away remotely controlled an ultrasound probe and ultrasound settings. | **Sample size:** 18  **Gestation:** 1^st^, 2^nd^ & 3^rd^ trimester  **Distance between sites:** 605km  **Recruitment location:** Canada  **High/Low risk patients:** NR | **Environment:** Community centre  **Operator**: Sonographer  **Training:** 1 hour  **Technology:** Telerobotic system (Melody) and videoconferencing equipment. | **Environment:** Hospital  **Receiver:** Radiologist  **Training:** NR  **Technology:** View images on Melody system or PACS. Additional videoconferencing equipment. | **Communication:** Internet  **Transmission:** Synchronous & asynchronous  **Frame rate:** NR  **Resolution:** NR  **Bandwidth:** 5Mb/s  **Transmission delay:** Up to 5-10 seconds in some cases. | Of 11 limited obstetrical exams, radiologists indicated images were adequate in nine (81%) cases, adequate with some reservations in one (9%) case and inadequate in one (9%) case.  Of 10 second-trimester complete obstetrical exams, radiologists indicated images were adequate in two (20%) cases, adequate with some reservations in three (30%) cases and inadequate in five (50%) cases. |
| Ahmed 2024  (86) | Two centres based in separate countries provided a joint diagnostic and counselling service over a period of 14 months. The primary centre performed the fetal echocardiography with a Voluson E10 machine, and images were transmitted live using Zoom OPS system with video-consultation and counselling. | **Sample size:** 513  **Gestation:** 2^nd^  **Distance between sites:** NR  **Recruitment location:** 2 sites (one reported as Egypt)  **High/Low risk patients:** high | **Environment:** Hospital  **Operator**: One of two fetal medicine physicians  **Training:** NR  **Technology:** OBS Studio was downloaded onto a laptop, which was connected to the GE machine by and HDMI cable | **Environment:** Hospital  **Receiver:** Paediatric cardiologist  **Training:** NR  **Technology:** The ZOOM platform was used to allow the paediatric consultant to link into the consultation and view the scanning images live. | **Communication:** Internet  **Transmission:** Synchronous  **Frame rate:** NR  **Resolution:** NR  **Bandwidth:** NR  **Transmission delay:** NR | Over a period of 12 months, 513 high-risk fetuses were then scanned, and out of these, 27 had congenital malformations. The most common were hypoplastic left heart syndrome (HHLS) and atrio-ventricular septal defect. Tele-echocardiography and counselling were successful in all the cases. Satisfaction with the service was 3.8/4, with the main limitation being the need for further referral to a tertiary centre for delivery |
| Arbeille 2014 (26) | To design and test a new telesonography technique using remote volume acquisition by untrained operators in locations without access to trained sonographers, postprocessing, and interpretation done at expert centres. | **Sample size:** 8  **Gestation:** NR  **Distance between sites:** NR  **Recruitment location:** Romania, French Guyana and Spain  **High/Low risk patients:** NR | **Environment:** Hospital  **Operator**: Nonexpert operator  **Training:** 1 hour  **Technology:** GE Voluson 730 transabdominal ultrasound, transmitted to an expert center using VirtualDub version 1.9.9 free software | **Environment:** Hospital  **Receiver:** Sonographer  **Training:** NR  **Technology:** Files converted to jpeg images. CNES software used the two-dimensional sonograms to create a virtual three-dimensional image of the volume scanned during the acquisition. | **Communication:** Internet  **Transmission:** Asynchronous  **Frame rate:** <12 fps  **Resolution:** NR  **Bandwidth:** 1Mb/s  **Transmission delay:** Ranged from 4 to 8 minutes if only one acquisition was required and from 11 to 19 minutes if three acquisitions were necessary. | Organs scanned in the obstetrical cases were adequately visualized by the expert in seven of eight (88%) examinations of the fetal head, femur, and umbilical cord and eight of eight (100%) examinations of the fetal abdomen and placenta |
| Arbeille 2005 (27) | To design a method for conducting fetal ultrasound examinations in isolated hospital sites using a dedicated remotely controlled robotic arm (teleechography). | **Sample size:** 29  **Gestation:** 3^rd^  **Distance between sites:** 1700km  **Recruitment location:** Spain  **High/Low risk patients:** Both | **Environment:** Hospital  **Operator**: Non-sonographer (doctor, paramedic)  **Training:** NR  **Technology:** Sonosite 180 and a GE 400 ultrasound machines. The audio, video and robot control information were transferred between the patient and expert sites using the Eutelsat W1 satellite link. The positioning of the probe holder (robot) was controlled in real time by the expert using video images transmitted from the isolated site to the expert site by videoconferencing. | **Environment:** Hospital  **Operator**: Radiologist, obstetrician, midwife  **Training: ≤** 1 hour  **Technology:** A sonographer moves a fictive probe (expert probe) which is connected to a computer (Computer 1). This expert probe simulates the ultrasound probe. Computer 1 sends the coordinate changes induced by the expert probe movements to the computer located at the patient site (Computer 2). Computer 2 controls the robotic arm, which reproduces the movements of the expert probe in the ultrasound probe positioned on the patient’s abdomen. | **Communication:** Telephone or satellite  **Transmission:** Synchronous  **Frame rate:** 15 fps  **Resolution:** NR  **Bandwidth:** 200 Kb/s  **Transmission delay:** 1 second | In 93.1% of the cases, all biometric parameters, placental location and amniotic fluid volume, were correctly assessed using the teleoperated robotic arm. In two cases, femur length could not be correctly measured. The mean duration of fetal ultrasound examination was 14 min (range, 10–18) and 18 min (range, 13–23) by conventional and tele-echography methods, respectively. The mean number of times the robotic arm was repositioned on the patient’s abdomen was seven (range, 5–9). |
| Arbeille 2016 (28) | A commercially available portable echograph was modified to allow functions (Doppler, two-dimensional, three-dimensional, elastography, etc.) and settings (gain, depth, freeze, record, etc.) to be teleoperated through an Internet connection. | **Sample size:** 15  **Gestation:** NR  **Distance between sites:** 60 km and 7000km  **Recruitment location:** France  **High/Low risk patients:** NR | **Environment:** Hospital  **Operator**: Non-sonographer operator (physician or paramedic)  **Training:** NR  **Technology:** required to hold and maintain the motorized ultrasound probe motionless on the patient in the location indicated by the expert sonographer through the videoconference connection | **Environment:** Hospital  **Receiver:** Expert but experience not noted  **Training:** 1 hour of training with the system  **Technology:** A basic portable computer with dummy probe was used to teleoperate the ultrasound remotely. Additional videoconferencing between sites. | **Communication:** Internet  **Transmission:** Synchronous  **Frame rate:** <10 fps  **Resolution:** NR  **Bandwidth:** 1Mb/s  **Transmission delay:** 2-3 seconds | Similar quality of ultrasound images allowing for medical diagnoses. Bandwidth and the frame rate of the transmitted video fluctuated. Due to the lag, the expert was required to make only single movements of the dummy probe at slow speeds. In some cases, it was difficult to obtain the appropriate view for diagnoses with this restricted movement, resulting in longer examination time. |
| Axelrod 2025  (87) | Prospective study alternating between remote and in-clinic weekly visits. A physician concluded the meeting with remote sonographic assessment of amniotic fluid maximal vertical pocket that together with a modified biophysical assessment. Study assessed the feasibility of remote visits. | **Sample size:** 20  **Gestation:** 3^rd^  **Distance between sites:** NR  **Recruitment location:** Israel  **High/Low risk patients:** High | **Environment:** Home  **Operator**: Patient  **Training:** The patients were required to complete a self- examination to the midwife's satisfaction as part of the training  **Technology:** Measurement of the maximum vertical pool by ultrasound using the Pulsenmore system to generate an modified biophysical profile. | **Environment:** Hospital  **Receiver:** Physician  **Training:** NR  **Technology:**, Live virtual communication with the healthcare provider, partly by video through the Datos Ltd. Platform. | **Communication:** Internet  **Transmission:** Synchronous  **Frame rate:** NR  **Resolution:** NR  **Bandwidth:** NR  **Transmission delay:** NR | Remote visits had a success rate of 97.4% (38 of 39), with significantly shorter durations compared with in-clinic visits (median 59.0 min vs. 159.0 min, P < 0.001).Women expressed high satisfaction (6.6 of 7), and adherence with recording fasting glucose values during the study period was significantly higher than the following period until birth (92.2% vs. 61.8%, P = 0.001) |
| Chan 2000 (34) | A live think was established between to hospitals, 1500km apart. Objective was to evaluate the clinical value of a tertiary teleultrasound consultation service. | **Sample size:** 24  **Gestation:** NR  **Distance between sites:** 1500km  **Recruitment location:** Australia  **High/Low risk patients:** High | **Environment:** Hospital  **Operator**: Sonographer  **Training:** NR  **Technology:** ATL 5000 ultrasound machine with transabdominal probe. Videoconferencing equipment (Picture Tel Venue 2000 and VTel LC 5000). | **Environment:** Hospital  **Receiver:** Materno-fetal expert  **Training:** NR  **Technology:** High resolution medical monitor with videoconferencing equipment. | **Communication:** Internet (ISDN)  **Transmission:** Synchronous  **Frame rate:** 30 fps  **Resolution:** NR  **Bandwidth:** 384 Kb/s for 96.2% of consultations, increased to 1.15Mb/s for the remaining 3.8%.  **Transmission delay:** NR | Overall, the consultations resulted in some modifications to the clinical diagnosis in 45.8% of the cases, and modifications to the management plan in 33.3% of the cases. Clinicians rated the teleconsultations highly and were confident in making telemedicine diagnoses. Patients rated the consultations highly. Feasibility demonstrated with high acceptability. |
| Chan 2001 (35) | Established a real-time fetal tele-ultrasound consultation service in Queensland, which had been integrated into routine clinical practice. Evaluation of services. | **Sample size:** 71  **Gestation:** NR  **Distance between sites:** 1500km  **Recruitment location:** Australia  **High/Low risk patients:** High | **Environment:** Hospital  **Operator**: Sonographer  **Training:** NR  **Technology:** Modem ultrasound equipment with videoconferencing equipment. | **Environment:** Hospital  **Receiver:** Clinician  **Training:** NR  **Technology:** Videoconferencing equipment. | **Communication:** Internet (ISDN)  **Transmission:** Synchronous  **Frame rate:** NR  **Resolution:** NR  **Bandwidth:** 384 Kb/s and 1Mb/s  **Transmission delay:** NR | Overall, the consultations resulted in some modifications to the clinical diagnosis in 41% of the cases, and modifications to the management plan in 40% of the cases. All significant anomalies diagnosed by teleconsultation were confirmed postnatally. Clinicians rated the teleconsultations highly and were confident in making telemedicine diagnoses. Patients rated the consultations highly. The teleultrasound service resulted in a net saving of A$6340. |
| El Guindi 2013 (39) | To highlight the value of 3D ultrasound in the prenatal assessment of fetal cardiovascular anomalies through offline diagnosis and/or second opinion (e.g. via internet link) | **Sample size:** 33  **Gestation:** NR  **Distance between sites:** 7096km  **Recruitment location:** French Guyana  **High/Low risk patients:** High | **Environment:** Hospital  **Operator**: NR  **Training:** NR  **Technology:** Volusen 730 Pro. Images sent via internet to diagnosis reference centre. | **Environment:** Hospital  **Receiver:** Independent examiner  **Training:** NR  **Technology:** NR | **Communication:** Internet  **Transmission:** Asynchronous  **Frame rate:** NR  **Resolution:** NR  **Bandwidth:** NR  **Transmission delay:** NR | 33 fetuses with 38 cardiac malformations were evaluated. Isolated cardiovascular malformations were detected in 23 fetuses. Extracardiac abnormalities were identified in 8 fetuses. Offline analysis of cardiovascular anomalies conferred significant diagnostic advantages over 2D ultrasound |
| Ferlin 2012 (40) | First-trimester ultrasound images transmitted in real-time using low-cost telecommunications. A prospective sample of fetal ultrasound images from 11 to 13+6 weeks of pregnancy was obtained. The examinations were transmitted in real-time to three independent examiners who carried out a qualitative assessment. | **Sample size:** 20  **Gestation:** 1^st^ trimester  **Distance between sites:** NR  **Recruitment location:** Brazil  **High/Low risk patients:** NR | **Environment:** Hospital  **Operator**: NR  **Training:** NR  **Technology:** Medison ultrasound device with multi-frequency convex probe. Images transferred form machine using external capture card and sent to PC. Transmitted in real-time using commercial Adobe Connect Pro. | **Environment:** Hospital  **Receiver:** Independent observer  **Training:** NR  **Technology:** Observed examinations in real-time and used ‘print screen’ to capture image to make measurements. Images saved for later evaluation of quality | **Communication:** Internet  **Transmission:** Asynchronous & synchronous  **Frame rate:** 30 fps  **Resolution:** 640 x 480 pixels  **Bandwidth:** 10 Mb/s  **Transmission delay:** 1-3 seconds for synchronous transmission | All fetal structures could be viewed and the quality of images received by the examiners was considered normal. There were significant differences for crown-rump length and nuchal translucency in the transmitted images but the loss in definition was acceptable. Thus, the quality of images transmitted via the Internet through the use of low-cost software appeared suitable for screening for chromosomal abnormalities in the first trimester of pregnancy. |
| Ferreira 2014 (41) | Fetal CNS structures were obtained by images and video clips. The exams were transmitted in real-time using a broadband internet and an inexpensive video streaming device. Four blinded examiners evaluated the quality of the exams using the Likert scale. | **Sample size:** 15  **Gestation:** 2^nd^ trimester  **Distance between sites:** 13320km  **Recruitment location:** Brazil  **High/Low risk patients:** Low | **Environment:** Hospital  **Operator**: Examine with minimum of 10 years ultrasound experience.  **Training:** NR  **Technology:** Acuson X300 ultrasound machine with convex multi-frequential transducer. WVS-01Ap commercial video encoder which receives video signal from ultrasound machine and converts to a format for transmission. | **Environment:** Hospital  **Receiver:** Independent observer  **Training:** NR  **Technology:** WVS-01Ap software decrypts the video clips and images and then sorted on an iMac for viewing. | **Communication:** Internet  **Transmission:** Synchronous  **Frame rate:** NR  **Resolution:** NR  **Bandwidth:** 384 Kb/s upload; 1.6 Mb/s download  **Transmission delay:** NR | The quality of the original video clips was slightly better than that observed by the transmitted video clips. In 47/60 comparisons (78.3%; 95% CI ¼ 66.4–86.9%) the quality of the video clips were judged to be the same. In 182/240 still images (75.8%; 95% CI ¼ 70.0–80.8%) the scores of transmitted images were considered the same as the original. Using an inexpensive video streaming device provided images of subjective good quality. |
| Fisk 1995 (43) | Live ultrasound images were transmitted in real-time, and two-way video and voice contact was used to communicate between the two centres. | **Sample size:** 6  **Gestation:** NR  **Distance between sites:** 120km  **Recruitment location:** UK  **High/Low risk patients:** NR | **Environment:** Hospital  **Operator**: Sonographer  **Training:** NR  **Technology:** Sonolayer ultrasound machine. Data compressed using videocodec and transmitted in real-time. Camera in the scanning room for viewing with audio from sonographer provided through light-weight headset. | **Environment:** Hospital  **Receiver:** Consultant  **Training:** NR  **Technology:** Identical videocodec with 59cm TV screen for viewing. Small camera mounted on TV for teleconsultation. | **Communication:** Telephone  **Transmission:** Synchronous  **Frame rate:** 30 fps  **Resolution:** 352 x 288 pixels  **Bandwidth:** 2 Mb/s  **Transmission delay:** NR | The consultants who used the link found themselves confidently making diagnoses and carrying out counselling over it. This was felt to be largely due to the skill of the sonographers in detecting potential problems, and their ability to provide the appropriate views on request. |
| Hishitani 2014 (45) | Telediagnosis system using ultrasound image transmission. The effect of telediagnosis, using a medical link between local maternity hospitals and the children’s medical center was verified. | **Sample size:** 117  **Gestation:** NR  **Distance between sites:** NR  **Recruitment location:** Japan  **High/Low risk patients:** High | **Environment:** Hospital  **Operator**: Sonographer  **Training:** NR  **Technology:** Transmission of recorded ultrasound images stored on DVD or real-time images transmitted directly from the ultrasound machine. Telecommunication consisted of headphones, microphone, a Web camera and personal computer. | **Environment:** Hospital  **Receiver:** Paediatric cardiologist  **Training:** NR  **Technology:** Telecommunication consisted of headphones, microphone, a Web camera and personal computer. | **Communication:** Telephone/internet  **Transmission:** Asynchronous & synchronous  **Frame rate:** 30 fps  **Resolution:** NR  **Bandwidth:** 6 Mb/s  **Transmission delay:** NR | Many severe cases were transferred to tertiary centres with the correct diagnosis; consequently, the number of emergent transportations of neonates with severe cardiac anomalies continued to drop. Telediagnosis was also useful as an educational tool for maternity hospital staff, who improved their skills during conversations with a specialist. Unlike in the outpatient clinic, consultation by telediagnosis was requested even for cases of mild abnormalities, and the number of false-positives increased, while many cardiac anomalies were found in the early stage. |
| Inamura 2021 (48) | Remote diagnoses of congenital heart disease using the spatio-temporal image correlation (STIC) method in collaboration with a local obstetric clinic. | **Sample size:** 2  **Gestation:** 3^rd^ trimester  **Distance between sites:** NR  **Recruitment location:** Japan  **High/Low risk patients:** High | **Environment:** Hospital  **Operator**: NR  **Training:** NR  **Technology:** STIC data transmitted via a virtual private network line. | **Environment:** Hospital  **Receiver:** NR  **Training:** NR  **Technology:** Transferred data rebuilt as an image on a personal computer using a View PAL system. | **Communication:** Internet  **Transmission:** Asynchronous  **Frame rate:** NR  **Resolution:** NR  **Bandwidth:** NR  **Transmission delay:** NR | Since the STIC method can make multiple cross sections, making a diagnosis by the STIC method was useful for the remote diagnosis. High definition flow render mode clearly showed left aortic arch, so it was possible to diagnose double aortic arch. |
| Ishikawa 2023 (49) | General surgeons conducted medical interviews and performed fetal sonography using an obstetrician videoconference system at the main central hospital | **Sample size:** 16  **Gestation:** 2^nd^ & 3^rd^ trimester  **Distance between sites:** 60km  **Recruitment location:** Japan  **High/Low risk patients:** High & Low | **Environment:** Hospital  **Operator**: General surgeon  **Training:** NR  **Technology:** Cloud-based video communication system used for real-time telecommunication. | **Environment:** Hospital  **Receiver:** Obstetrican-gynaeoclogist  **Training:** NR  **Technology:** NR | **Communication:** Internet  **Transmission:** Synchronous  **Frame rate:** NR  **Resolution:** NR  **Bandwidth:** NR  **Transmission delay:** NR | The participants underwent a median of two remote antenatal checkups. According to a questionnaire survey, 90.0%, 80.0%, and 70.0% of the pregnant women perceived improvements in their physical, mental, and economic burdens, respectively. |
| Jemal 2024 (50) | Thirteen healthcare providers in the North Shoa Zone in Ethiopia completed training to enable them to perform antenatal ultrasound with the remote supervision of an obstetrician via a tele-ultrasound platform. Image interpretations between obstetricians and healthcare providers were compared. | **Sample size:** NR (100 scans)  **Gestation:** 2^nd^ & 3^rd^ trimester  **Distance between sites:** NR  **Recruitment location:** Ethiopia  **High/Low risk patients:** NR | **Environment:** Community  **Operator**: Healthcare professionals  **Training:** 3-week training programme  **Technology:** Lumify ultrasound probe connected to a Samsung Galaxy tablet computer. Videoconferencing to transfer ultrasound data and communicate with receiver. | **Environment:** Hospital  **Receiver:** Obstetrician  **Training:** NR  **Technology:** Image recordings were uploaded wirelessly for the research team to review on the online dashboard. | **Communication:** Internet  **Transmission:** Synchronous  **Frame rate:** NR  **Resolution:** NR  **Bandwidth:** 4 Mb/s  **Transmission delay:** NR | Of 100 exams randomly selected to assess concordance between healthcare providers’ and obstetricians’ image interpretations, concordance ranged from 79% to 100% for each parameter assessed. 99.4% of participants surveyed indicated that they would recommend antenatal ultrasound using tele-ultrasound to friends and family. Themes relating to participants’ experiences of having a tele-ultrasound exam were reduced travel and cost, equivalence in quality of virtual care to in-person care and empowerment through diagnostic information. |
| Leighton 2019 (54) | Observational electronic health record data are used to compare maternal and childbirth outcomes between patients receiving care via telemedicine or in-person visits through regression analysis. Average patient time and resources saved are calculated, and patient satisfaction scores are reported. | **Sample size:** 6757 (6302 in-person consult, 455 telemedicine consult)  **Gestation:** NR  **Distance between sites:** 71 and 160 miles  **Recruitment location:** USA  **High/Low risk patients:** Both | **Environment:** Hospital  **Operator**: NR  **Training:** NR  **Technology:** Face-to-face consultations using secure video technology.  Images from the study are electronically transmitted in real time for interpretation. | **Environment:** Hospital  **Operator**: Materno-fetal specialist  **Training:** NR  **Technology:** NR | **Communication:** Internet  **Transmission:** Synchronous  **Frame rate:** NR  **Resolution:** NR  **Bandwidth:** NR  **Transmission delay:** NR | Telemedicine patients experienced similar outcomes to the in-person group, indicating that MFM telemedicine can serve as an effective substitute for in-person care. MFM telemedicine patients saved $90.28 per consult in travel and work related expenses. An overwhelming majority of MFM telemedicine patients were satisfied with their visit and indicated that they would be interested in receiving care via telemedicine in the future. |
| Le Vance 2025 (89) | Feasibility study using home ultrasound and cardiotocography in 15 high risk women. Primary outcome of the home ultrasound was completion of an interpretable ultrasound for fetal movements, fetal heartbeat and objective assessment of the liquor volume. | **Sample size:** 15  **Gestation:** 3^rd^ trimester  **Distance between sites:** NR  **Recruitment location:** UK  **High/Low risk patients:** High | **Environment:** Home  **Operator**: Patient  **Training:** Face-to-face education session prior to device usage  **Technology:** Pulsenmore device, validated for assessing the liquor volume objectively, fetal heartrate and fetal movements. | **Environment:** Hospital  **Receiver:** Researcher/obstetrician  **Training:** NR  **Technology:** Obstetrician supervised the HCPs in performing the ultrasound exams and was available to provide real-time support and ongoing feedback on image acquisition and interpretation through the tele-ultrasound platform | **Communication:** Internet  **Transmission:** Asynchronous  **Frame rate:** NR  **Resolution:** NR  **Bandwidth:** NR  **Transmission delay:** NR | Fifteen participants completed 24 remote ultrasounds. Overall, the fetal heartbeat, movements and an assessment of the liquor volume were identified in 92%, 83% and 100% of all ultrasound scans respectively. 79% of all scans had all three criteria unanimously assessed. Neither ethnicity, parity, BMI nor fetal presentation were significant factors for achievement of the primary outcome for both devices. There was non-significant reduction in anxiety scores before and after device usage (p=0.19). |
| Malone 1997 (57) | Patients had a complete fetal anatomic survey recorded onto videotape by a trained ultrasonographer. A live interactive video telemedicine link was then established, and a perinatologist directed the ultrasonographer through the anatomy survey. Subsequently a different perinatologist blinded to the telemedicine interpretation, reviewed the videotaped examination. | **Sample size:** 200  **Gestation:** 2^nd^ & 3^rd^ trimester  **Distance between sites:** NR  **Recruitment location:** USA  **High/Low risk patients:** NR | **Environment:** Hospital  **Operator**: Sonographers  **Training:** NR  **Technology:** ATL Ultramark 9 HDI or ATL Apogee 800 plus ultrasound machines. Each office contained a personal computer attached to a 38cm monitor was installed, including a Canon video camera, speakerphone with headset. Images were digitalised and data compressed to appropriate resolution capacity. | **Environment:** Hospital  **Receiver:** Perinatologist  **Training:** NR  **Technology:** Identical telemedicine platform installed at central facility, but with a 42cm monitor for live viewing. Videotaped scans were viewed on at least a 40cm monitor. | **Communication:** Telephone  **Transmission:** Asynchronous & synchronous  **Frame rate:** 15 fps  **Resolution:** 352 x 288 pixels  **Bandwidth:** 384 kb/s  **Transmission delay:** NR | Telemedicine and videotape interpretations provided similar scores in 84% of scans. In 17 of the 33 anatomic categories telemedicine provided significantly better scores than videotape, whereas in the remaining 16 anatomic categories the scores were equivalent. More videotape than telemedicine examinations required repeat ultrasonography because of suboptimal imaging (10% vs 3%, p = 0.003). |
| Michailidis 2001 (63) | Thirty sequential normal singleton pregnancies were included in the study. Four cardiac volumes were acquired using a three-dimensional ultrasound system. The volumes were sent via the Internet to a tertiary fetal cardiology center, where a detailed fetal cardiac examination was attempted using the three-dimensional volumetric dataset. | **Sample size:** 30  **Gestation:** 2^nd^ & 3^rd^ trimester  **Distance between sites:** NR  **Recruitment location:** UK  **High/Low risk patients:** Low | **Environment:** Hospital  **Operator**: Sonographers  **Training:** NR  **Technology:** Volusen 530D ultrasound machine. Images then sent via internet via email. | **Environment:** Hospital  **Operator**: Fetal cardiologist  **Training:** NR  **Technology:** The transmitted volume datasets appeared as an incoming e-mail on a standard personal computer using conventional software at the fetal cardiology center. The datasets were decompressed using proprietary software (3D View). | **Communication:** Internet  **Transmission:** Asynchronous  **Frame rate:** NR  **Resolution:** NR  **Bandwidth:** NR  **Transmission delay:** NR | A complete heart examination was accomplished in 23 of 30 cases. The four-chamber view and the cardiac situs were seen in all cases. The right ventricular outflow tract was seen in 29 (96.7%) cases and the left ventricular outflow tract in 25 (83.3%) cases. The long-axis view of the aortic arch, superior vena cava, inferior vena cava and pulmonary veins were visualized in more than 80% of cases. |
| Nelson 2001 (66) | Patients were studied at 2 institutions using high-end two-dimensional clinical ultrasonographic scanners and commercially available three-dimensional ultrasonography for a variety of organ systems (first- and second trimester fetus, abdomen, and female pelvis). | **Sample size:** 56  **Gestation:** 1^st^ & 2^nd^ trimester  **Distance between sites:** 2857 miles  **Recruitment location:** USA  **High/Low risk patients:** NR | **Environment:** Hospital  **Operator**: Sonographers  **Training:** NR  **Technology:** Two and three-dimensional ultrasonographic data were acquired using conventional clinical ultrasonographic equipment. Transfer of volume data between machines and systems was accomplished using magneto-optical disks via overnight courier service and Internet transfer. | **Environment:** Hospital  **Operator**: Radiologist  **Training:** NR  **Technology:** The physician reviewed the hard copy images and also volume data directly as deemed necessary. | **Communication:** Internet and courier  **Transmission:** Asynchronous  **Frame rate:** NR  **Resolution:** NR  **Bandwidth:** NR  **Transmission delay:** NR | Overall, three-dimensional ultrasonography could produce diagnostic-quality results comparable with those of two-dimensional ultrasonography. Three-dimensional ultrasonographic image quality was lower than that of two-dimensional ultrasonography. Two- and three-dimensional ultrasonographic measurements were comparable (<5% difference) as was the extent of organ visualization, although some structures were challenging for both two- and three-dimensional ultrasonography. |
| Nir 2024 (67) | Patients used a self-operated ultrasound device/s to complete a modified biophysical profile. Total visit length was measured for both the in-person first visit and the subsequent telemedicine encounter. A patient satisfaction survey form was obtained. | **Sample size:** 10  **Gestation:** 3^rd^ trimester  **Distance between sites:** NR  **Recruitment location:** Israel  **High/Low risk patients:** Low | **Environment:** Hospital  **Operator**: Patient  **Training:** Instructed in person by an obstetrician how to operate the remote devices.  **Technology:** Patients viewed the US image and fetal heart rate on the smartphone screen. Audio and visual communication was established for the evaluation. | **Environment:** Hospital  **Operator**: Obstetrician  **Training:** NR  **Technology:** Audio and visual communication was established for the evaluation. | **Communication:** Internet  **Transmission:** Synchronous  **Frame rate:** NR  **Resolution:** NR  **Bandwidth:** NR  **Transmission delay:** NR | Nine women (90%) were able to complete remote modified biophysical profile assessment. For one participant, fetal assessment was not completed due to technically inconclusive fetal monitoring. Another participant was referred for additional assessment in the delivery room. Satisfactory amniotic fluid volume measurements were achieved in 100% of participants. The telemedicine encounter was significantly shorter (93.1 ± 33.1 min) than the in-person visit (247.2 ± 104.7 min) |
| Nores 1997 (68) | All patients had a sonographic evaluation of the uterus, adnexa, and gestational sac recorded onto videotape by a trained sonographer. A live, interactive video telemedicine link was established, and a perinatologist directed the sonographer through the scan. Subsequently, a different perinatologist, blinded to the telemedicine interpretation, reviewed the original videotaped examination. | **Sample size:** 100  **Gestation:** 1^st^ trimester  **Distance between sites:** NR  **Recruitment location:** USA  **High/Low risk patients:** Low | **Environment:** Hospital  **Operator**: Sonographers  **Training:** NR  **Technology:** ATL Apogee 800 plus and ATL Ultramark 9 HDI ultrasound machines used. The data is transmitted via a data card interface to the inverse multiplexer, which is configured with three integrated services digital network lines. A live, interactive video telemedicine link was established, and a perinatologist observed and directed the sonographer through the study.  Studies are sent by courier to our unit for review and interpretation by experienced perinatologists | **Environment:** Hospital  **Operator**: Perinatologist  **Training:** NR  **Technology:** A different perinatologist, blinded to the telemedicine interpretation, reviewed the still images and videotaped examination. | **Communication:** Telephone and courier  **Transmission:** Asynchronous & synchronous  **Frame rate:** 15 fps  **Resolution:** 352 x 288 pixels  **Bandwidth:** 384 Kb/s  **Transmission delay:** NR | Telemedicine and videotape review scores were the same in 95 cases, and the final diagnosis was identical in 98 cases. In three cases, telemedicine had higher scores than videotape review, whereas in two cases, videotape review scores were higher. |
| Oelmeier 2023 (69) | Objective aimed at establishing a telemedicine network of specialists in MFM for interprofessional exchange regarding high-risk pregnancies. The aims were to evaluate the providers’ attitude toward the telemedicine solutions and to quantify the number of inpatient appointments that were avoided through interprofessional video consultations. | **Sample size:** 59  **Gestation:** NR  **Distance between sites:** NR  **Recruitment location:** Germany  **High/Low risk patients:** High | **Environment:** Hospital  **Operator**: NR  **Training:** NR  **Technology:** For remote counseling, the ELVI software was used. Video consultations with the specialist team could be solicited via phone or email and were carried out upon request. Depending on the provider’s preference and technical possibilities, ultrasound images were shared using the store-and-forward method, screen sharing, or in real time. | **Environment:** Hospital  **Operator**: Materno-fetal specialist  **Training:** NR  **Technology:** NR | **Communication:** NR  **Transmission:** Asynchronous & synchronous  **Frame rate:** NR  **Resolution:** NR  **Bandwidth:** NR  **Transmission delay:** NR | Overall, 47% (33/70) of the scheduled visits were avoided after video consultation. The providers’ tendency to refrain from sending their patients to the university Hospital was statistically noticeable (p = 0.048). |
| Pardo 2025 (92) | Evaluating the utilization of a self-operated home ultrasound service. This service provides a handheld, app-connected ultrasound device for remote basic fetal monitoring, with its use determined at the discretion of the patient as a supplement - rather than a replacement - to standard prenatal care. | **Sample size:** 107,167  **Gestation:** 1^st^,2^nd^ & 3^rd^  **Distance between sites:** NR  **Recruitment location:** Israel  **High/Low risk patients:** Both | **Environment:** Home  **Operator**: Patient  **Training:** Five-segment video tutorial within the application  **Technology**: Pulsenmore home ultrasound device | **Environment:** Hospital  **Operator**: Trained obstetrical sonographer - either an ultrasound technician or OBGYN physician  **Training:** NR  **Technology:** Uploaded to a secure cloud, readily available for professional review via the clinician’s dashboard | **Communication:** Internet  **Transmission:** Asynchronous  **Frame rate:** NR  **Resolution:** NR  **Bandwidth:** NR  **Transmission delay:** NR | Users had higher socioeconomic scores, were more primiparous and had a higher incidence of chronic disease and pregnancy complications. Preterm birth rates and adverse neonatal outcomes did not differ between groups. Device utilization, both overall and stratified by actual utilization degree, was safe and not associated with any maternal, obstetrical or neonatal adverse pregnancy outcomes. |
| Pontones 2023 (70) | Two cohorts using two different mobile ultrasound systems. The participants examined the fetal heartbeat, fetal profile and amniotic fluid. Aspects of feasibility and acceptance were evaluated using a questionnaire. Success rates in relation to image and video quality were evaluated by healthcare professionals. | **Sample size:** 46  **Gestation:** 2^nd^ & 3^rd^ trimester  **Distance between sites:** NR  **Recruitment location:** Germany  **High/Low risk patients:** Both | **Environment:** Hospital  **Operator**: Patient  **Training:** NR  **Technology:** Pulsenmore mobile ultrasound system and Butterfly iQ ultrasound system. The images and videos obtained were saved on the study phone and then transferred to the study server. The videos were saved as MP4 files and the images were saved as PNG or JPG files. | **Environment:** Hospital  **Operator**: Ultrasound examiner  **Training:** NR  **Technology:** NR | **Communication:** Internet  **Transmission:** Asynchronous  **Frame rate:** NR  **Resolution:** NR  **Bandwidth:** NR  **Transmission delay:** NR | Two thirds of the women were able to imagine performing the self-guided examination at home, but 87.0% would prefer live support by a professional. Concerns about their own safety and that of the child were expressed by 23.9% of the women. Success rates for locating the target structure were 52.2% for videos of the fetal heartbeat, 52.2% for videos of the amniotic fluid in all four quadrants and 17.9% for videos of the fetal profile. |
| Reddy 2000 (72) | Remote community underwent two obstetric ultrasound examinations. The first used teleultrasound. The second examination, women travelled to a regional hospital and had an ultrasound examination performed under the direct supervision of a radiologist. | **Sample size:** 49  **Gestation:** NR  **Distance between sites:** NR  **Recruitment location:** Canada  **High/Low risk patients:** NR | **Environment:** Hospital  **Operator**: Technologist  **Training:** Supervised by a radiologist  **Technology:** Using an AVP Pacspro Teleradiology System comprising of 2 personal computers linked via 19.2-kbit/s modem over a single analog telephone line. | **Environment:** Hospital  **Operator**: Radiologist  **Training:** NR  **Technology:** NR | **Communication:** Telephone  **Transmission:** Synchronous & Asynchronous  **Frame rate:** NR  **Resolution:** NR  **Bandwidth:** 19.2 Kb/s  **Transmission delay:** NR | The technical quality of the transmitted images was reported as excellent. The teleradiology system shortened the time it took for patients to be informed of their examination results (1 week on average for the control group versus the same day for the teleradiology group). |
| Schwartz 2021 (73) | A retrospective review of fetal telecardiology visits between March 15 and July 15, 2020, was performed. The chart was reviewed for confirmation of diagnosis postnatally. | **Sample size:** 122  **Gestation:** 2^nd^ & 3^rd^ trimester  **Distance between sites:** <4 – 23 miles  **Recruitment location:** USA  **High/Low risk patients:** High | **Environment:** Hospital  **Operator**: Sonographer  **Training:** NR  **Technology:** Four rooms were set up with cameras and the study could be viewed live. The cameras also allow for direct conversations with the pediatric cardiologist reading the study. At all sites, after the study was completed, picture archiving and communications system (PACS) access through a secure remote desktop was used for image review and reporting through the imaging system. Studies were transmitted to be reviewed in real time or later in the day. | **Environment:** Hospital  **Operator**: Pediatric cardiologist  **Training:** NR  **Technology:** After the fetal echocardiogram was complete, the pediatric cardiologist reviewing the study would counsel the family on the results through videoconferencing or set up a follow-up appointment to discuss in person with the family. | **Communication:** Internet  **Transmission:** Asynchronous & synchronous  **Frame rate:** NR  **Resolution:** NR  **Bandwidth:** NR  **Transmission delay:** NR | With pandemic onset, there was a large increase in the number of telemedicine visits with a total of 122 mothers seen between five MFM clinics. Fourteen mothers (11.5%) had abnormal fetal echocardiograms requiring additional follow-up, and seven mothers (5.8%) had a fetal echocardiogram suspicious for a critical congenital heart disease (CCHD). All the fetal echocardiograms suspicious for CCHD were confirmed on postnatal echocardiograms. To our knowledge, none of the normal fetal echocardiograms were found to have congenital heart disease postnatally. |
| Sharma 2003 (74) | Live fetal echocardiograms were transmitted at 384 kbits/s from the remote primary care center. | **Sample size:** 34  **Gestation:** 2^nd^ & 3^rd^ trimester  **Distance between sites:** <4 – 23 miles  **Recruitment location:** USA  **High/Low risk patients:** Both | **Environment:** Hospital  **Operator**: Sonographer  **Training:** NR  **Technology:** HP 5500 ultrasound system with broadband/fusion technology were used. Guided by telecommunication by pediatric specialists. | **Environment:** Hospital  **Operator**: Pediatric cardiology fellow or a pediatric echocardiography technician **Training:** NR  **Technology:** The receiving unit consisted of a commercially available system equipped with a 4000ZX codec using a proprietary SG3 algorithm. | **Communication:** Internet  **Transmission:** Synchronous  **Frame rate:** NR  **Resolution:** NR  **Bandwidth:** NR  **Transmission delay:** NR | During live screening transmitted at 384 kbits/s from the primary care center, 3 of 34 fetuses were diagnosed with heart disease. Surveys from patients with direct physician contact and by telemedicine showed high satisfaction with telemedicine-assisted screening and counseling. |
| Shields 2022 (75) | Converting the materno-fetal medicine clinic from a conventional medical treatment model to a telemedicine platform. We compared clinical productivity between the two models. | **Sample size:** NR  **Gestation:** NR  **Distance between sites:** NR  **Recruitment location:** USA  **High/Low risk patients:** Both | **Environment:** Hospital  **Operator**: Sonographer  **Training:** Supervised by a radiologist  **Technology:** After the ultrasound was completed, the sonographer would communicate with the physician and review the images; if no further imaging was recommended, the sonographer initiated a ZoomTM telehealth visit with the physician. | **Environment:** Hospital  **Operator**: Physician  **Training:** NR  **Technology:** NR | **Communication:** Internet  **Transmission:** Asynchronous  **Frame rate:** NR  **Resolution:** NR  **Bandwidth:** NR  **Transmission delay:** NR | We had a significant decrease in patient visits following introduction. However, our average daily patient visits per fulltime equivalent (FTE) were only marginally reduced (11.1 visit per FTE versus 7.6 visits per FTE, p < 0.0001), resulting in a relative decrease in adjusted work relative value units (6987 versus 5440). There was an increase in more basic follow-up ultrasound procedures, complexity over comprehensive follow-up ultrasound procedures, after conversion. Despite similar proportions of new consults, there was an increase in the proportion of follow-up visits and medical decision-making complexity evaluation and management. |
| Soong 2002 (77) | Establishing a tertiary level tele-ultrasound service. Performing real-time ultrasound examination under the direction of the subspecialist. | **Sample size:** 160  **Gestation:** NR  **Distance between sites:** 1500km  **Recruitment location:** Australia  **High/Low risk patients:** NR | **Environment:** Hospital  **Operator**: Sonographer  **Training:** NR  **Technology:** The sonographer at the remote end then performs the real-time ultrasound examination under the direction of the subspecialist. Videoconferencing consisted of either a room-based and PC-based system. The room-based system contains a more powerful video codec and uses a television style monitor in a large room, whereas a PC-based system uses a computer monitor. Interactive two-way microphones allow direct communication between the two centres. | **Environment:** Hospital  **Operator**: Materno-fetal specialist  **Training:** NR  **Technology:** Videoconference machine with high resolution screen. Interactive two-way microphones allowed for direct communication between the two centres. | **Communication:** Internet  **Transmission:** Synchronous  **Frame rate:** NR  **Resolution:** NR  **Bandwidth:** 384 Kb/s  **Transmission delay:** A review of our first 160 consultations showed that re-transmission during a consultation occurred in 32 cases | In over 93% of the consultations, the set up time of all the equipment for a consultation at the remote end is less than 30 minutes. 94% of the women strongly agreed or agreed when asked if their privacy and confidentiality were maintained during the videoconference. All women who responded indicated that they would recommend this type of videoconferencing to others. |
| Vinals 2005 (80) | To assess whether the spatio-temporal image correlation (STIC) acquisition technique can be taught to a general obstetrician by e-mail; whether STIC volume datasets can be transmitted over the Internet; and whether STIC volume datasets analyzed offline at a remote setting can be used to confirm or exclude major cardiac defects. | **Sample size:** 50  **Gestation:** 2^nd^ and 3^rd^  **Distance between sites:** NR  **Recruitment location:** Chile  **High/Low risk patients:** NR | **Environment:** Hospital  **Operator**: Obstetrician  **Training:** NR  **Technology:** Voluson 730 Expert series ultrasound scanner used to acquire images. Stored on private hard disk and transmitted via the internet. | **Environment:** Hospital  **Operator**: NR  **Training:** NR  **Technology:** Offline analysis was performed using the four-dimensional (4D) View software version 1 and 2.1. | **Communication:** Internet  **Transmission:** Asynchronous  **Frame rate:** NR  **Resolution:** NR  **Bandwidth:** 300 Kb/s and 600 Kb/s  **Transmission delay:** Upload took 20-40 seconds | A telemedicine link via the Internet was possible in all cases. Seventy-seven volume datasets were sent to the web server. A complete cardiac examination according to set criteria was achieved by the administrator in 86% of the cases scanned by one operator and 95% of the cases scanned by the other operator. Three patients had cardiac defects confirmed postnatally, two fetuses had extracardiac anomalies and one fetus had a suspected cardiac defect unconfirmed by second-opinion TELESTIC. There were two isolated major congenital heart defects. |
| Vinayak 2018 (81) | Evaluation of point of care ultrasound (POCUS) training through teleultrasound for midwives to deploy antenatal ultrasound services within the community. | **Sample size:** 271 scans  **Gestation:** NR  **Distance between sites:** 20 -400km  **Recruitment location:** Kenya  **High/Low risk patients:** NR | **Environment:** Community  **Operator**: Midwives  **Training:** A detailed curriculum was developed to train sonography-naïve midwives to deliver POCUS and accurately identify high risk pregnancies.  **Technology:** Tablet platform, mobile phone transmission and teleradiology to connect remotely with hospital-based specialists in a process of image and diagnostic verification. | **Environment:** Hospital  **Operator**: Specialist  **Training:** NR  **Technology:** NR | **Communication:** Internet  **Transmission:** Asynchronous  **Frame rate:** NR  **Resolution:** NR  **Bandwidth:** NR  **Transmission delay:** 4 minutes | 271 ultrasound scans were performed and 220 patients were able to be located for post-delivery follow-up. Post-delivery accuracy of scans performed by the midwives was 99.63%. The average turn-around time for post scan validation was 12 minutes of which 4 minutes was required for transmission of scans to the base hospital. No problems were reported in respect to the mobile phone, modem or CCC system. |
| **Diagnostic accuracy studies** | | | | | | |
| Adams 2018 (23) | To determine the feasibility of a telerobotic approach to remotely perform prenatal sonographic examinations | **Sample size:** 30  **Gestation:** 2^nd^ and 3rd  **Distance between sites:** NR  **Recruitment location:** Canada  **High/Low risk patients:** NR | **Environment:** Hospital  **Operator**: Midwives  **Training:** NR  **Technology:** Conventional ultrasound used the EPIQ 5; Philips Healthcare system. Robotic ultrasound used the  MELODY patient system, SonixTablet ultrasound system, and 5-MHz transducer and MELODY Expert system, consisting of a mock transducer and an electronic control box. Videoconference between sonographer and patient. | **Environment:** Hospital  **Operator**: Radiologist  **Training:** NR  **Technology:** NR | **Communication:** Internet  **Transmission:** Asynchronous  **Frame rate:** NR  **Resolution:** NR  **Bandwidth:** 50 Mb/s download and 20 Mb/s upload speed  **Transmission delay:** NR | No statistically significant difference between conventional and telerobotic measurements of fetal head circumference, biparietal diameter, or single deepest vertical pocket of amniotic fluid; however, a small but statistically significant difference was observed in measurements of abdominal circumference and femur length (P< .05). Intraclass correlations showed excellent agreement (>0.90) between telerobotic and conventional measurements of all 4 biometric parameters. Of 21 fetal structures included in the anatomic survey, 80% of the structures attempted across all patients were sufficiently visualized by the telerobotic system (range, 57%– 100% per patient). |
| Adriaanse 2012 (25) | To evaluate the clinical accuracy of four-dimensional (4D) echocardiography in the detailed prenatal diagnosis of congenital heart disease (CHD) in a telemedicine setting | **Sample size:** 10  **Gestation:** 2^nd^  **Distance between sites:** 391km, 190km, 36km  **Recruitment location:** Netherlands  **High/Low risk patients:** High | **Environment:** Hospital  **Operator**: Sonographer  **Training:** NR  **Technology:** Voluson E8 (GE Medical Systems, Zipf, Austria) with a realtime 3D abdominal probe (RAB 4–8-D-MHz). 4D View version 9.1 (GE Medical Systems) was used for offline analysis. | **Environment:** Hospital  **Operator**: One observer was a fetal and pediatric cardiologist and two were maternal–fetal medicine specialists, specializing in fetal echocardiography.  **Training:** NR  **Technology:** NR | **Communication:** NR  **Transmission:** Asynchronous  **Frame rate:** NR  **Resolution:** NR  **Bandwidth:** NR  **Transmission delay:** NR | In two cases all observers correctly diagnosed all details of the volume datasets. The observer with the best performance reached perfect agreement in six cases and nearly perfect agreement in three. The volumes were most frequently studied by sectional planes and were analyzed in a median time of 11.0 (range, 2.5–30.0) min. The median confidence score was 4.0 (range, 1.0–5.0). |
| Beldjerd 2022 (29) | To assess the potential of the use of asynchronous teleexpertise (ASTE) to provide prenatal diagnosis from a medical and economic point of view. | **Sample size:** 260  **Gestation:** 1^s^**^t,^** 2^nd^ and 3^rd^  **Distance between sites:** 82km  **Recruitment location:** France  **High/Low risk patients:** High | **Environment:** Hospital  **Operator**: Midwife sonographer  **Training:** NR  **Technology:** Cloud-based communication, image management, and documentation platform Tricefy® | **Environment:** Hospital  **Operator**: Expert  **Training:** NR  **Technology:** TriceFy1 platform certified as a health data host with CE marking, via the sending of a question associated with images and/or video loops from the various ultrasound examinations. | **Communication:** Internet  **Transmission:** Asynchronous  **Frame rate:** NR  **Resolution:** NR  **Bandwidth:** NR  **Transmission delay:** NR | The results revealed a 90.68% feasibility of transmitting in a satisfactory and interpretable way ultrasound images and videos via the tele-expertise platform (292/322 files). In our series, asynchronous analysis allowed the required physician to make an accurate diagnosis and identify 74 (28.5%, 95% CI [23% –33.9%]) pregnancies associated with malformations and rule out abnormalities in 186 (71.5%, 95% CI [66.1% –77%]) of the cases. The ASTE was not associated with face-to-face consultations for 72.7% (189/260) of the patients, who without moving, were able to have access to a precise diagnosis by ruling out the presence of anomalies in 163/189 of these patients and confirming them in 26/189 patients. The practice of ASTE would result from a societal point of view, an average saving of 61.8% (€ 120.57) per patient compared to a face-to-face consultation. |
| Bolin 2020 (32) | To describe the implementation and effectiveness of a statewide fetal tele-echocardiography program serving a resource-limited population. Retrospectively reviewed all fetal tele-echocardiograms performed through 2018. Yearly statewide prenatal detection rates of operable congenital heart disease were queried from the Society of Thoracic Surgeons database. | **Sample size:** 1164  **Gestation:** 2^nd^ and 3^rd^  **Distance between sites:** NR  **Recruitment location:** USA  **High/Low risk patients:** High | **Environment:** Hospital  **Operator**: Sonographer  **Training:** NR  **Technology:** General Electric Voluson I for almost all studies; in the last 2 years, a General Electric E95 and a Phillips IE33 ultrasound machine was used. Cardiology consultation with families was performed in real-time over a Tandberg Video Conference System. | **Environment:** Hospital  **Operator**: Fetal cardiologist  **Training:** NR  **Technology:** Interpreted in real time by a trained fetal cardiologist over teleconferencing. The images were stored as digital clips on our local server or burned to a DVD and stored for subsequent review. | **Communication:** Internet  **Transmission:** Synchronous  **Frame rate:** NR  **Resolution:** NR  **Bandwidth:** 100 Mb/s  **Transmission delay:** NR | Fetal tele-echocardiography identified all types of congenital heart disease, with a sensitivity of 74% and specificity of 97%. For the detection of ductal-dependent congenital heart disease, fetal tele-echocardiography was 100% sensitive and specific. Between 2009 and 2018, annual statewide prenatal detection rates of congenital heart disease requiring heart surgery in the first 6 months of life rose by 159% (17–44%; R2 = 0.88, p < 0.01). |
| Brown 2017 (33) | A clinical fetal tele-echo service was 3 h from the nearest congenital heart surgeon. The aim of this study was to determine if fetal tele-echo utilizing local sonographers at a small regional hospital can accurately and efficiently identify fetuses with complex CHD. | **Sample size:** 75  **Gestation:** 2^nd^ and 3^rd^  **Distance between sites:** 190 miles  **Recruitment location:** USA  **High/Low risk patients:** High | **Environment:** Hospital  **Operator**: Sonographer  **Training:** Fetal echocardiography training was provided by a pediatric cardiologist over a 4-month period. Training consisted of formal lectures with handouts and video examples and 6 days of life scanning with the pediatric cardiologist.  **Technology:** Philips iE33 ultrasound machine. Images were stored at and also sent via the Internet for interpretation  Using an encrypted virtual private network with network firewalls on both ends. | **Environment:** Hospital  **Operator**: Paediatric cardiologist  **Training:** NR  **Technology:** Reviewed and interpreted the images within 24 hours, and a typewritten report was faxed to the requesting provider | **Communication:** Internet  **Transmission:** Asynchronous  **Frame rate:** NR  **Resolution:** NR  **Bandwidth:** NR  **Transmission delay:** NR | Fetal tele-echoes were Correct in 21%, Likely Correct in 56%, showed Major Differences in 0%, and showed Minor Differences in 23%. For identifying complex CHD, fetal tele-echo had a sensitivity and specificity of 100%. The average number of fetal echocardiograms per mother– infant pair was 1.1. |
| Cuneo 2019 (36) | To evaluate a fetal telecardiology program in a medically underserved area. Obstetric ultrasonographers performed fetal echocardiograms (local site) that were read in real time. | **Sample size:** 368  **Gestation:** 2^nd^ and 3^rd^  **Distance between sites:** NR  **Recruitment location:** USA  **High/Low risk patients:** High | **Environment:** Hospital  **Operator**: Obstetric ultrasonographers  **Training:** Training period lasted approximately 3 months.  **Technology:** Bundled optical carrier with 1–192 fibers and 51 Mbps 29.6 Gbps and a DS3 line which connected the local and the distant sites. | **Environment:** Hospital  **Operator**: Fetal cardiologist  **Training:** NR  **Technology:** Fetal cardiologist reviewed the patient’s intake form and the fetal echocardiographic images captured by the ultrasonographer. If echocardiographic images were unclear or nondiagnostic, the ultrasonographer re-scanned the patient while the fetal cardiologist watched. | **Communication:** Internet  **Transmission:** Asynchronous  **Frame rate:** NR  **Resolution:** NR  **Bandwidth:** 51 Mbps - 9.6 Gbps  **Transmission delay:** NR | All mothers preferred having their fetal cardiac evaluations performed locally as opposed to traveling to the distant center. The estimated cost to parents for fetal cardiac evaluation at the distant center was nine times greater than that of telecardiology ($581 vs $61). Congenital heart disease or arrhythmia was diagnosed in 28 and 15 fetuses, respectively; there was one false-negative result. All fetuses were correctly risk-stratified with respect to delivery location. |
| Day 2025 (88) | Study used artificial intelligence (AI) to automatically extract video clips of the fetal heart from a stream of ultrasound video, and to assess the performance of these when used for remote second review | **Sample size:** 48  **Gestation:** 2^nd^  **Distance between sites:** NR  **Recruitment location:** UK  **High/Low risk patients:** Both | **Environment:** Hospital  **Operator**: Sonographers  **Training:** Training period lasted approximately 3 months.  Technology: GE Voluson Expert 22 machine. AI tool then took every frame of the ultrasound scan video obtained by the sonographer, and automatically classified each frame into one of 13 standard image plane label | **Environment:** Hospital  **Operator**: Five experts in fetal cardiology  **Training:** None provided  **Technology** | **Communication:** Internet  **Transmission:** Asynchronous  **Frame rate:** NR  **Resolution:** NR  **Bandwidth:** NR  **Transmission delay:** NR | The initial manual scan had a sensitivity of 0.792 and specificity of 0.917 for detecting congenital heart disease in this cohort. The addition of second review improved the sensitivity to 0.975 using video clips, which was significantly higher than using still images(0.892, p = 0.002. The median review time was 1.0 min (IQR 0.71) for the still images, and 3.75 min (IQR 3.12) for the AI‐generated video clips. |
| Dougherty 2021 (37) | Compressed movie clip ultrasound images (obstetric sweep protocol) obtained by minimally trained personnel were read and interpreted by physicians with training in obstetric ultrasound. Observed findings were compared among readers and between each reader and the gold standard ultrasound scan report. | **Sample size:** 91  **Gestation:** 2^nd^  **Distance between sites:** NR  **Recruitment location:** USA  **High/Low risk patients:** NR | **Environment:** Hospital  **Operator**: Two fourth-year medical students  **Training:** Trained by a radiologist assistant  **Technology:** GE LOGIQ i system  ultrasound transducer is passed over the abdomen in 6 sweeps, and a series of volumetric images are recorded as a cine or movie clip. Images were deidentified and saved to a McKesson picture archiving and communication system. | **Environment:** Hospital  **Operator**: A radiologist, a maternal-fetal medicine specialist and an obstetrics and gynecology generalist  **Training:** NR  **Technology:** McKesson picture archiving and communication system. | **Communication:** NR  **Transmission:** Asynchronous  **Frame rate:** NR  **Resolution:** NR  **Bandwidth:** NR  **Transmission delay:** NR | The agreements among readers and between readers and the gold standard, for the anterior and posterior variables of the placental location were excellent, with Cohen κ values of 0.81 to 0.88 and 0.77 to 0.9, respectively. Cohen κ values were slight or slight/fair for other placental locations (left, right, fundal, and low), and the sensitivity and specificity ranged widely. The agreement among readers and between readers and the gold standard for fetal number comparisons was also excellent, with Cohen κ values ranging from 0.82 to 1, sensitivity from 0.83 to 1, and specificity from 0.99 to 1. The agreement among readers for fetal presentation comparisons, according to the Cohen κ, ranged from 0.79 to 0.85 and between readers and the gold standard had values of 0.43 to 0.49. For biometric parameters and estimated gestational age calculations based on these parameters, inter-reader reliability ranged from 0.79 to 0.85 for all parameters except femur length. Greater than 94% of obstetric sweep protocol ultrasound ages were within 7 days of the corresponding gold standard age. |
| Ferrer-Roca 2006 (42) | Acquisitions were carried out through an existing 2-dimesional device adding a magnetic tracking system on the ultrasound probe. Probe positioning and video output was introduced into a running software that allows the generation of 2-dimensional orthogonal and 3-dimensional volume images, as well as tele-consultation. | **Sample size:** 32  **Gestation:** 1^st^, 2^nd^ and 3^rd^  **Distance between sites:** NR  **Recruitment location:** Spain  **High/Low risk patients:** NR | **Environment:** Hospital  **Operator**: NR  **Training:** NR  **Technology:** Aloka-SSD 680 device connected to a dual Pentium II computer at 450 MHz. The B-mode images taken from the video output of the ultrasound device were digitalized and processed by the TeleInVivoTM volume visualization software. | **Environment:** Hospital  **Operator**: NR  **Training:** NR  **Technology:** NR | **Communication:** Internet and ISDN  **Transmission:** Asynchronous  **Frame rate:** 18 fps  **Resolution:** NR  **Bandwidth:** 64 Kb/s  **Transmission delay:** Average transmission time was 7.8+/-6.3 minutes on Internet and 3.0+/-1.4 minutes through ISDN. | Final volumes were small (1.5 Mb) and required about 4±2 min to be transmitted over one ISDN channel (64 Kbs). Good correlation (k=0.7) was found between local and distant diagnoses. In 30%, images were considered of low quality and in 29% of good quality; diagnosis could be done with confidence in all except 7 cases. |
| Hadar 2022 (44) | Participants were instructed to perform up to three ultrasound scans per day, with a minimum of at least one daily. The maximum number of scans was limited within the device’s application to a maximum of three in a single 24-h period with at least 30 min required between each scan, to avoid excessive use of the device. Every scan was segmented into six separate recordings, each lasting 15–45 s, totaling 3 min per scan. | **Sample size:** 100  **Gestation:** 1^st^, 2^nd^ and 3^rd^  **Distance between sites:** NR  **Recruitment location:** Israel  **High/Low risk patients:** NR | **Environment:** Home  **Operator**: Patient  **Training:** NR  **Technology:** INSTINCT ultrasound device, a portable wireless ultrasound transducer, physically attached through a type-c USB connection to a smartphone. Each participant received the device for a self-use period of 7– 14 days, along with a Galaxy S8 Samsung smartphone with an already installed application by Pulsenmore used to operate it. The scans were stored on the internal memory of the smart phone, and were downloaded upon completion of the study period, when each participant returned her device after use. | **Environment:** Hospital  **Operator**: Obstetrician–gynecologist or an experienced ultrasound technician.  **Training:** NR  **Technology:** NR | **Communication:** NR  **Transmission:** Asynchronous  **Frame rate:** NR  **Resolution:** NR  **Bandwidth:** NR  **Transmission delay:** NR | Success in detection was 95.3% for fetal heart activity, 88.3% for body movements, 69.4% for tone, 92.2% for normal amniotic fluid volume, and 23.8% for breathing movements. Interobserver agreement was 94.4% for fetal heart rate activity, 85.9% for body movements, 69.5% for fetal tone, 86.9% for amniotic fluid volume, and 94.0% for breathing movements. Self-assessed user experience was rated at 4.4/5, whereas device satisfaction was rated at 3.9/5. |
| Inamura 2020 (47) | Heart screening for all pregnant women at four obstetrics clinics over the three years from 2009 to 2014. The spatio-temporal image correlation (STIC) data from 15,404 examinations in normal pregnancies were analyzed. Obstetricians and sonographer collected STIC data from four-chamber view images. Eight pediatric cardiologists analyzed the images offline. | **Sample size:** NR  **Gestation:** NR  **Distance between sites:** NR  **Recruitment location:** Japan  **High/Low risk patients:** Low | **Environment:** Hospital  **Operator**: Sonographer  **Training:** NR  **Technology:** Transferred the STIC data via a virtual private network. Using the transferred data, images were reconstructed on a personal computer using the View PAL system | **Environment:** Hospital  **Operator**: Pediatric cardiologists  **Training:** NR  **Technology:** Images were reconstructed on a personal computer using the View PAL system. | **Communication:** Internet  **Transmission:** Asynchronous  **Frame rate:** NR  **Resolution:** NR  **Bandwidth:** NR  **Transmission delay:** NR | A normal heart was diagnosed in 14,002 cases (90.9%), an abnormal heart was diagnosed in 457 cases (3.0%), and poor images were obtained in 945 cases (6.1%). 138 cases had congenital heart disease (CHD) after birth, and severe CHD necessitating hospitalization occurred in 36 cases. We were not able to detect CHD by screening in 12 cases. The sensitivity and specifcity of STIC in CHD screening was 50% and 99.5%, respectively. The sensitivity and specifcity of STIC in screening for severe CHD was 82% and 99.9%, respectively. |
| Kern-Goldberger 2021 (51) | All ultrasounds were performed by sonographers remotely trained under a standardized protocol and interpreted by maternal-fetal medicine physicians via telemedicine. The primary outcome was potential missed diagnosis of a fetal anomaly, defined as an ultrasound designated as normal by a sonographer but diagnosed with an anomaly by a maternal-fetal medicine physician via telemedicine. | **Sample size:** NR  **Gestation:** 1st, 2^nd^ and 3^rd^  **Distance between sites:** NR  **Recruitment location:** USA  **High/Low risk patients:** Both | **Environment:** Hospital  **Operator**: Sonographer  **Training:** An initial sonographer competency assessment and an intensive training module for sonographers with focus on standard protocols from the American Institute of Ultrasound in Medicine.  **Technology:** A cloudbased picture archiving and communication system to allow for immediate access to transferred images. | **Environment:** Hospital  **Operator**: Maternal–fetal medicine specialist  **Training:** NR  **Technology:** NR | **Communication:** Internet  **Transmission:** Asynchronous  **Frame rate:** NR  **Resolution:** NR  **Bandwidth:** NR  **Transmission delay:** NR | Overall, 6403 ultrasound examinations were evaluated, 310 of which had a diagnosis of fetal anomaly by a maternal-fetal medicine physician (4.8%). Of the fetal anomalies, 43 were diagnosed on an anatomic survey (13.9%), and 89 were diagnosed as cardiac anomalies (28.7%). The overall rate of the potential missed diagnoses was 34.5% and varied significantly by type of ultrasound (anatomy scans vs other first-, second-, and third-trimester ultrasounds) (P<.01). |
| Kozuki 2016 (85) | To assess the feasibility of ultrasonographic task shifting by estimating the accuracy at which primary level health care workers can perform community-based third-trimester ultrasound diagnosis for selected obstetric risk factors in rural Nepal. | **Sample size:** 804  **Gestation:** 3^rd^  **Distance between sites:** NR  **Recruitment location:** Nepal  **High/Low risk patients:** NR | **Environment:** Home  **Operator**: Three auxiliary nurse–midwives  **Training:** Two 1-week ultrasound trainings together with the trainings set one month apart.  **Technology:** Sonosite Nanomaxx portable ultrasound system was used. Images that represented those diagnoses were saved on the ultrasound machine. Images sent by e-mail to a radiologist in Kathmandu if concerns. Otherwise, they were downloaded at the end of the week on to a sever. | **Environment:** Hospital  **Operator**: Radiologists  **Training:** NR  **Technology:** ach reviewer was instructed to log onto the server with a personalized username and password and fill out an online form next to each set of images to make their diagnostic assessments | **Communication:** Internet  **Transmission:** Asynchronous  **Frame rate:** NR  **Resolution:** NR  **Bandwidth:** NR  **Transmission delay:** NR | A total of 804 women contributed to the analysis. Each auxiliary nurse–midwife’s k statistic for diagnosis of non-cephalic presentation was above 0.90 compared with the ultrasonogram reviewers. Sensitivity, specificity, and positive and negative predictive values were between 90% and 100% for all auxiliary nurse–midwives |
| Landwehr 1997 (53) | Routine ultrasonographic studies from 35 patients were remotely interpreted. Evaluation included a blinded comparison of the sonographer's assessment of 38 fetal structures with that of the physician at the tertiary care center. Technical evaluation included system reliability, and the number of digital telephone lines required for adequate real-time visualization. | **Sample size:** 35  **Gestation:** 1st, 2^nd^ and 3^rd^  **Distance between sites:** NR  **Recruitment location:** USA  **High/Low risk patients:** NR | **Environment:** Hospital  **Operator**: Sonographer  **Training:** NR  **Technology:** Ultrasonography was performed with a Siemens Sonoline SI-450. A commercial telemedicine system was installed on two IBM-compatible personal computer (PC) systems, one at the remote site, a university-based teaching clinic, and one at the tertiary center. | **Environment:** Hospital  **Operator**: Second-year maternal-fetal medicine fellow  **Training:** NR  **Technology:** NR | **Communication:** ISDN lines  **Transmission:** Synchronous  **Frame rate:** NR  **Resolution:** NR  **Bandwidth:** 384 Kb/s  **Transmission delay:** NR | There was complete consistency of interpretation for 25 of 38 (66%) fetal structures. Thirteen structures had discrepancies in visualization, reflecting a difference in the adequacy of visualization, not the normalcy or identity of the structures. Three digital (integrated switching digital network, ISDN) telephone lines were required for real-time visualization. |
| Mabuchi 2020 (55) | This study retrospectively analyzed telediagnosis cases from 2009 to 2018 in six geographically remote hospitals. Three-dimensional ultrasonographic images from these referral hospitals were forwarded through an optical fiber network system for analysis. The primary endpoint was accuracy of prenatal diagnosis. | **Sample size:** 182  **Gestation:** 2^nd^ and 3^rd^  **Distance between sites:** NR  **Recruitment location:** Japan  **High/Low risk patients:** High | **Environment:** Hospital  **Operator**: Sonographer  **Training:** NR  **Technology:** Voluson E7, E8, or E10 images using B-mode and color Doppler to create multiple STIC datasets were transferred by pressing one transmission button. Transferred via an optical fiber-based wide area network | **Environment:** Hospital  **Operator**: Maternal–fetal specialists  **Training:** NR  **Technology:** Transmitted STIC datasets were saved on a hard disk and analyzed using ViewPal software. | **Communication:** Internet  **Transmission:** Asynchronous  **Frame rate:** NR  **Resolution:** NR  **Bandwidth:** 10 Mb/s  **Transmission delay:** NR | Congenital heart disease (CHD) was detected in 14.9% of cases (24/161); the accuracy of prenatal diagnosis was 95.0% (153/161). Seven severe cases with CHD required immediate postnatal surgical or medical treatment. The remaining 17 cases considered suitable for delivery at the referral hospitals were delivered there, and they did not require intervention immediately after birth. |
| Manley 2024 (59) | To evaluate the effectiveness of a tech-enabled telesonography service in an under-resourced obstetrics and gynecology clinic. Quantitative data were collected for each sonographic exam to determine its efficiency and accuracy. A survey was also offered to measure patient satisfaction. Data were collected and analyzed to produce early trends following the implementation of the service. | **Sample size:** 460  **Gestation:** NR  **Distance between sites:** NR  **Recruitment location:** USA  **High/Low risk patients:** NR | **Environment:** Hospital  **Operator**: Healthcare worker  **Training:** Proprietary program that includes learning modules and hands-on practice.  **Technology:** Voluson S8 ultrasound machine. TeleScan, a cloud-based software for telecommunication. | **Environment:** Hospital  **Operator**: Maternal-fetal medicine sonographer  **Training:** The sonographers were all TeleScan-certified through a proprietary program that included learning modules, practice in a demo environment, workflow and software proficiency assessments during clinic hours, and ongoing training for each subsequent software release.  **Technology:** Review the cine clips, provide annotations and measurements, and compile a preliminary diagnostic report for the provider to review. The provider accessed the report through the TeleScan software, and could modify the report, remeasure structures, add their conclusions, and sign the final report before relaying results to the patient. | **Communication:** Satellite  **Transmission:** Synchronous  **Frame rate:** NR  **Resolution:** NR  **Bandwidth:** NR  **Transmission delay:** NR | The tech-enabled telesonography service decreased patient exam and report turnaround times in an underresourced OB/Gyn clinic, which correlated with decreases in the time until the next appointment and the clearing of scheduling backlogs. Importantly, this efficiency was achieved without forfeiting diagnostic accuracy or patient satisfaction. |
| McCrossan 2011 (60) | An initial fetal echocardiogram was performed by a radiographer followed by a second transmitted to the regional centre, in real time, via a telemedicine link with live guidance by a fetal cardiologist. A fetal echocardiogram was performed later at the regional centre (reference standard). Structured questionnaires were employed to evaluate the technical quality of each tele-link and the radiographers’ confidence at performing fetal echocardiograms. | **Sample size:** 67  **Gestation:** 2^nd^  **Distance between sites:** NR  **Recruitment location:** UK  **High/Low risk patients:** High | **Environment:** Hospital  **Operator**: Sonographer  **Training:** NR  **Technology:** Teleconferences were transmitted in real-time via ISDN6. At both sending and receiving sites, a Tandberg 880 videoconferencing unit was employed. | **Environment:** Hospital  **Operator**: Fetal cardiologist  **Training:** NR  **Technology:** At both sending and receiving sites, a Tandberg 880 videoconferencing unit was employed. | **Communication:** ISDN  **Transmission:** Synchronous  **Frame rate:** NR  **Resolution:** NR  **Bandwidth:** NR  **Transmission delay:** NR | 69 remote fetal echocardiograms were performed and showed 58 normal hearts and 11 with congenital heart disease (CHD). Telemedicine was accurate in 97% of cases compared with the reference standard (κ score=0.89) indicating excellent agreement. All tele-links connected at first attempt with a mean study time = 13.9 min. Overall tele-link quality was rated highly (median=4/5). In 94% of tele-links, at least 11/12 components of the FE were confidently assessed. The mean composite radiographer’s questionnaire score increased significantly during the study period (p<0.05). |
| Meiman 2022 (62) | A retrospective review of medical records was performed to identify patients with congenital heart disease who had cardiac surgery or intervention prior to one year of age. The rate of prenatal diagnosis prior to establishing any fetal tele‐echocardiography sites was compared to the rate of prenatal diagnosis after the sites were established. | **Sample size:** 1270 (750 telemedicine, 520 control)  **Gestation:** 2^nd^ & 3^rd^ trimester  **Distance between sites:** 80 -130miles  **Recruitment location:** USA  **High/Low risk patients:** High | **Environment:** Hospital  **Operator**: Sonographer  **Training:** Received training from pediatric cardiologists  **Technology:** Philips iE33, Philips Epiq 7, and General Electric Voluson E10 ultrasound machines used. The images were reviewed and interpreted within 24 hours of completion. | **Environment:** Hospital  **Operator**: Pediatric cardiologist  **Training:** NR  **Technology:** Interpretation using an encrypted Virtual Private Network with network firewalls on both ends | **Communication:** Internet  **Transmission:** Asynchronous  **Frame rate:** NR  **Resolution:** NR  **Bandwidth:** NR  **Transmission delay:** NR | The rate of prenatal diagnosis prior to the implementation of the first fetal tele‐ echocardiography site was 13.8% and after the sites were established, the prenatal diagnosis rate was 39.7% (p < 0.01). |
| Neito-Calvache 2024 (90) | 2 PAS imaging experts (teleconsultants) were selected to asynchronously review deidentified standardized grayscale and color Doppler ultrasound images for five patients who had completed treatment for placenta accreta spectrum (PAS), resulting in 60 individual teleconsultations. | **Sample size:** 60  **Gestation:** 2^nd^  **Distance between sites:** NR  **Recruitment location:** Argentina, Brazil, Colombia, Egypt, England, Ghana, Indonesia, Ireland, Italy, Taiwan, USA  **High/Low risk patients:** High | **Environment:** Hospital  **Operator**: Local Sonologist  **Training:** NR  **Technology:** B- mode and Doppler assessments were obtained by both transabdominal and transvaginal sonography | **Environment:** Hospital  **Operator**: Experts  **Training:** None of the teleconsultants had formal training in this classification system.  **Technology:** Each teleconsultant received the archived antenatal ultrasound images and were blinded to clinical history, surgical approach, and delivery outcomes. All participants were asked to report their ultrasound evaluation using a drawing including the uterus and placenta in three planes | **Communication:** Internet  **Transmission:** Asynchronous  **Frame rate:** NR  **Resolution:** NR  **Bandwidth:** NR  **Transmission delay:** NR | Teleconsultant antenatal evaluation and management plans matched those of the local team in 71.7% of the cases. When reports differed, PAS severity was overestimated in nine reviews (16.9%) and was underestimated in six reviews (11.3%). |
| Olsen 2025 (91) | Two-aimed study evaluates the diagnostic accuracy of robot-assisted ultrasound compared to traditional ultrasound and explores patient experiences. | **Sample size:** 46  **Gestation:** 2^nd^ & 3rd  **Distance between sites:** 140km  **Recruitment location:** Norway  **High/Low risk patients:** Low | **Environment:** Hospital  **Operator**: An assisting midwife  **Training:** NR  **Technology:** Robot arm held by midwife as sonographer operates it | **Environment:** Hospital  **Operator**: Sonographer  **Training:** NR  **Technology:** This communication consists of robot control and synchronization data, when the sonographer moves the probe at the table on the expert site | **Communication:** Internet  **Transmission:** Synchronous  **Frame rate:** NR  **Resolution:** NR  **Bandwidth:** NR  **Transmission delay:** In two examinations (not time specified) | Biometric measurements showed excellent reliability (intraclass correlation coefficient 0.990–0.993) with acceptable limits of agreement. Twenty questions about patient experiences were asked and 94% of the women scored for highest level of satisfaction. |
| Pardo 2024 (93) | A prospective, non-randomized, non-blinded clinical study design was used. Pulsenmore ES scans were obtained by non-professional laypersons in app guided (AG) mode (user follows video tutorials in the application) or clinician-guided (CG) mode (user is guided by a health care professional in a real-time telemedicine visit). The scans were stored on a cloud for later interpretation by a health care professional. | **Sample size:** 28  **Gestation:** 1^st^, 2^nd^ & 3rd  **Distance between sites:** NR  **Recruitment location:** Israel  **High/Low risk patients:** Both | **Environment:** Home  **Operator**: Patient  **Training:** NR  **Technology:** Pulsenmore home ultrasound device. US transducer coupled with a user smartphone application. | **Environment:** Hospital  **Operator**: Clinician  **Training:** NR  **Technology:** Images/videos reviewed on a clinician web-viewer dashboard | **Communication:** Internet  **Transmission:** Synchronous & asynchronous  **Frame rate:** NR  **Resolution:** NR  **Bandwidth:** NR  **Transmission delay:** NR | Pulsenmore tool for measurement of THE fetal heartrate was 84.7 +/- 11.24% of scans made in app-guided mode and 96.3 +/- 6.35% of scans made in clinician guided mode. Corresponding values for maximum vertical pool were 91.7 +/- 2.31% and 95.0 +/- 1.73%. Sensitivity (87.5% and 100% in app-guided and clinician-guided modes, respectively) and specificity (95% and 95.5% in app-guided and clinician-guided modes, respectively) were established for MVP. |
| Rabie 2017 (56) | Retrospective cohort study determining the sensitivity and accuracy of teleultrasound. In addition, we evaluated the number of ultrasound examinations required to complete an anatomic survey. | **Sample size:** 2368 ultrasounds  **Gestation:** 2^nd^ and 3^rd^  **Distance between sites:** NR  **Recruitment location:** USA  **High/Low risk patients:** High | **Environment:** Hospital  **Operator**: Sonographer  **Training:** Continuous quality control with additional supervision and training when indicated.  **Technology:** Teleultrasound is conducted in either a “store-and-forward” method with still images and cine clips or with real-time transmission and viewing of high-definition video. | **Environment:** Hospital  **Operator**: Maternal fetal specialist  **Training:** NR  **Technology:** Ultrasound examinations were viewed and interpreted on Viewpoint 6 software. | **Communication:** Internet  **Transmission:** Synchronous and Asynchronous  **Frame rate:** NR  **Resolution:** NR  **Bandwidth:** NR  **Transmission delay:** NR | The teleultrasound cohort had a congenital anomaly prevalence of 5.66%. The sensitivity of teleultrasound was 57.46%; the specificity was 98.21%; and the accuracy was 95.9%. Anatomic surveys were completed after 1 visit in 82% of patients, whereas 63% and 61% of the remaining patients required 2 and 3 visits, respectively. |
| Rabie 2019 (71) | To compare the sensitivity and accuracy of teleultrasound and demonstrate that teleultrasound is not inferior to on-site ultrasound in the pre-natal diagnosis of fetal anomalies. | **Sample size:** 5903 (2499 telemedicine, 3404 control)  **Gestation:** 2^nd^ & 3^rd^ trimester  **Distance between sites:** NR  **Recruitment location:** USA  **High/Low risk patients:** NR | **Environment:** Hospital  **Operator**: Sonographer  **Training:** Continuous quality control with additional training and supervision as needed.  **Technology:** NR | **Environment:** Hospital  **Operator**: Materno-fetal specialist  **Training:** NR  **Technology:** The interpreting MFM was provided with still images, cine clips and if requested, real-time viewing of the teleultrasound. The ultrasounds were viewed and interpreted using Viewpoint 5. | **Communication:** Internet  **Transmission:** Synchronous & Asynchronous  **Frame rate:** NR  **Resolution:** NR  **Bandwidth:** NR  **Transmission delay:** NR | The sensitivity of teleultrasound and on-site ultrasound was 57.46% and 76.57%, and the accuracy was 95.9% and 90.97%, respectively. The observed sensitivity difference was 0.1911. The accuracy, specificity, positive and negative predictive values of teleultrasound are similar to on-site ultrasound. |
| Toscano 2021 (84) | Aimed to test a novel, innovative Obstetric tele-diagnostic ultrasound system in which the imaging acquisitions were obtained by an operator without prior ultrasound experience using simple scan protocols based only on external body landmarks and uploaded using low-bandwidth internet for asynchronous remote interpretation by an off-site specialist. | **Sample size:** 125  **Gestation:** 2^nd^ and 3^rd^  **Distance between sites:** NR  **Recruitment location:** Peru  **High/Low risk patients:** NR | **Environment:** Hospital  **Operator**: One nurse and one care technician  **Training:** 4 hours of didactic sessions followed by 4 hours of hands-on training  **Technology:** Examinations performed using the portable Mindray DP-10 (Mindray, China). This was attached to a Windows 10 tablet containing the telemedicine software. A low- to moderate-speed, 3G internet connection in the health clinic allowed upload times within 5 min. | **Environment:** Hospital  **Operator**: Radiologist assessed the images and maternal-fetal specialist assessed the reference standard  **Training:** NR  **Technology:** Imaging data situated on the cloud. Clips are not viewed as video but rather as scrollable image stacks. | **Communication:** Internet  **Transmission:** Asynchronous  **Frame rate:** 10-15 fps  **Resolution:** NR  **Bandwidth:** NR  **Transmission delay:** 5 minutes | Obstetric ultrasound telediagnosis showed excellent agreement with standard of care ultrasound allowing the identification of number of fetuses (100% agreement), fetal presentation (95.8% agreement, κ =0.78 (p < 0.0001)), placental location (85.6% agreement, κ =0.74 (p < 0.0001)), and assessment of normal/abnormal amniotic fluid volume (99.2% agreement) with sensitivity and specificity > 95% for all variables. Intraclass correlation was good or excellent for all fetal biometric measurements (0.81–0.95). |
| TroyanoLuque 2013 (78) | Validate a new clinical obstetrics and gynecology application for a hand-held transvaginal ultrasound device. | **Sample size:** 25  **Gestation:** 1^st^  **Distance between sites:** NR  **Recruitment location:** Spain  **High/Low risk patients:** NR | **Environment:** Hospital  **Operator**: Specialist  **Training:** NR  **Technology:** The patients were initially examined with Vscan, followed by the Voluson730-E (V730-E). Images are stored locally on a micro-SD card and can be viewed using Vscan Gateway, which can be installed on any computer. For teleconsultation purposes, the images along with measurements and voice annotation were sent via e-mail. | **Environment:** Hospital  **Operator**: NR  **Training:** NR  **Technology:** NR | **Communication:** Internet  **Transmission:** Asynchronous  **Frame rate:** NR  **Resolution:** 420 by 320 pixels  **Bandwidth:** NR  **Transmission delay:** NR | Regarding lesion visibility with Vscan, optimal distance was 8–16 cm depending on the examination type, and the total detection rate was 98.7%. Assessment of reproducibility in 180 measurements showed that the measurements obtained with Vscan were 0.3–0.4 cm lower than those obtained with the high resolution US device (Voluson 730 Expert). Nevertheless, Pearson’s correlation coefficient was high for biparietal diameter (0.72). Image transport on USB and SD-flash cards proved convenient for telemedicine. |
| Vinals 2008 (79) | To assess whether spatiotemporal image correlation (STIC) volumes from fetuses at 11 + 0 to 13 + 6 weeks’ gestation can be obtained by a non-expert and whether fetal echocardiography can be performed via a telemedicine link, providing a remote and reproducible diagnosis of the fetal heart condition. | **Sample size:** 49  **Gestation:** 1^st^  **Distance between sites:** NR  **Recruitment location:** Peru  **High/Low risk patients:** NR | **Environment:** Hospital  **Operator**: Obstetrician  **Training:** Training in the interpretation of cardiac images from STIC volume datasets.  **Technology:** Voluson 730 ultrasound scanner. Volumes were uploaded from Lima, Peru, to a web disk on a private server and then downloaded to be analyzed by the two reviewers independently. | **Environment:** Hospital  **Operator**: NR  **Training:** NR  **Technology:** Offline analysis was performed following downloading from private server. | **Communication:** Internet  **Transmission:** Asynchronous  **Frame rate:** NR  **Resolution:** NR  **Bandwidth:** NR  **Transmission delay:** NR | The four chamber view obtained was apical in 22/35 (63%) cases and lateral in 13 (37%). Volume datasets were obtained after 12 weeks’ gestation in 30/35 fetuses. Three cases had nuchal translucency thickness above the 99th percentile, and two of these had an abnormal heart. Five cases had abnormal outcomes. A mean of 3 (range, 1–6) STIC datasets per patient were acquired. The kappa index obtained confirmed interobserver reliability, with good or very good concordance (kappa > 0.6) in 14/18 structures and views related to the heart. |
| Wootton 1997 (83) | Six subspecialists with considerable experience in fetal ultrasound viewed a selection of pre-recorded ultrasound scans. The scans were viewed in random order, at randomly selected bandwidths. Observers, who were blinded to both recording and bandwidth, assessed the technical quality on a five-point Likert scale. They also recorded their diagnosis. | **Sample size:** 49  **Gestation:** 1^st^, 2^nd^ and 3rd  **Distance between sites:** NR  **Recruitment location:** UK and Ireland  **High/Low risk patients:** Both | **Environment:** Hospital  **Operator**: Sonographer  **Training:** NR  **Technology:** Recorded on VHS videotape and made on a high-resolution ultrasound machine. | **Environment:** Hospital  **Operator**: NR  **Training:** NR  **Technology:** Viewed the scans on a large display monitor (29inches, 74cm) in an individual viewing booth 384or 1920kbit/s. | **Communication:** Internet  **Transmission:** Asynchronous  **Frame rate:** NR  **Resolution:** NR  **Bandwidth:** 384 Kb/s or 1920 Kb/s.  **Transmission delay:** NR | There was no significant difference in the perceived technical quality of the scans between the two bandwidths used (P=0.09). Of the 84 recordings transmitted at 1920kbit/s, 71(85%) were diagnosed correctly or ‘half correctly’ and13(15%) were misdiagnosed. Of the 95recordings transmitted at 384kbit/s, 66(69%) were diagnosed correctly or ‘half correctly’ and29(31%) were misdiagnosed. This difference was significant (P=0.03). The results indicate that although there were no perceived differences in technical quality between recordings transmitted at 384or 1920kbit/s, diagnostic accuracy was marginally worse at the lower bandwidth. |
| **Randomised controlled trials** | | | | | | |
| Mor 2024 (65) | Patients with a history of 2 or more prior abortions were randomized early in their subsequent pregnancy in a 1:1 ratio into either the control group, which received standard high-risk prenatal care, or the study group, which received additional twice-weekly home-ultrasound sessions. The home ultrasound scans assessed fetal pulse, movements, and amniotic fluid volume, aiming to provide maternal reassurance. Patients performed the scans themselves using the Pulsenmore device, with real-time guidance from a physician. Maternal anxiety was assessed using the validated questionnaires. | **Sample size:** 50 (25 in teleultrasound, 25 in control)  **Gestation:** 1^st^ and 2^nd^ trimester  **Distance between sites:** NR  **Recruitment location:** USA  **High/Low risk patients:** High | **Environment:** Home  **Operator**: Patient  **Training:** NR  **Technology:** Ultrasound scan assessing for a fetal heartbeat, amniotic fluid volume, fetal tone, and fetal movements was performed with live guidance and instructions from the physician. | **Environment:** Hospital  **Operator**: Physician  **Training:** NR  **Technology:** NR | **Communication:** Internet  **Transmission:** Synchronous  **Frame rate:** NR  **Resolution:** NR  **Bandwidth:** NR  **Transmission delay:** NR | There were no significant differences in demographics between the groups. The primary outcome (STAI score at the last visit) was significantly lower in the device group compared to the control group (P=.037). In addition, the study group exhibited a greater reduction in STAI scores between the first and last visits (P=.045), and a significantly higher MAAS score at the end of the follow-up period (P=.046). |
| Whittington 2022 (82) | This was a single center, randomized (1:1) noninferiority study. Individuals referred to the maternal–fetal medicine (MFM) ultrasound clinic were randomized to standard in-person ultrasound and counseling or teleultrasound and telemedicine counseling. The primary outcome was major fetal anomaly detection rate (sensitivity). | **Sample size:** 485 (294 in teleultrasound, 291 in control)  **Gestation:** 1^st^, 2^nd^ and 3^rd^  **Distance between sites:** NR  **Recruitment location:** USA  **High/Low risk patients:** High | **Environment:** Hospital  **Operator**: Sonographer  **Training:** NR  **Technology:** NR | **Environment:** Hospital  **Operator**: Materno-fetal specialist  **Training:** NR  **Technology:** Review the images from a telemedicine site within the building. | **Communication:** Internet  **Transmission:** Synchronous  **Frame rate:** NR  **Resolution:** NR  **Bandwidth:** NR  **Transmission delay:** NR | The sensitivity of sonographic detection of 28 anomalies was 82.14% in the control group and of 20 anomalies in the telemedicine group, it was 85.0%. The observed difference in sensitivity was 0.0286, much smaller than the proposed noninferiority limit of 0.05. Specificity, negative predictive value, positive predictive value, and accuracy were more than 94% for both groups. Patient satisfaction was more than 95% on all measures, and there were no significant differences in patient satisfaction based on maternal characteristics. |
| **Economic studies** | | | | | | |
| Beldjerd 2023 (30) | To evaluate the economic impact of asynchronous tele-expertise in obstetric ultrasound care in private medical practice through a comparison with face-to-face consultations. | **Sample size:** 260  **Gestation:** 1^st^, 2^nd^ and 3^rd^  **Distance between sites:** 82km  **Recruitment location:** France  **High/Low risk patients:** High | **Environment:** Hospital  **Operator**: Sonographer  **Training:** NR  **Technology:** All the requests for advice were realized and recorded on the TriceFy1 platform certified as a health data host with CE marking, via the sending of a question associated with images and/or video loops from the various ultrasound examinations. | **Environment:** Hospital  **Operator**: Expert  **Training:** NR  **Technology:** Cloud-based communication, image management, and documentation platform Tricefy®. | **Communication:** Internet  **Transmission:** Asynchronous  **Frame rate:** NR  **Resolution:** NR  **Bandwidth:** NR  **Transmission delay:** NR | The expected average total cost for tele-expertise for a patient was €74.45 (95% CI: €66.36–€82.54) compared to €195.02 (95% CI: €183.90–€206.14) for the conventional face-to-face strategy. Accordingly, using tele-expertise led to a statistically significant reduction of €120.57 in the average total cost per patient. A sensitivity analysis confirmed the robustness of the model produced. |
| Dowie 2008 (38) | Use of a telecardiology service for fetal cardiac diagnosis alongside an existing arrangement for referring pregnant women directly to perinatal cardiologists for detailed fetal echocardiography. | **Sample size:** 76  **Gestation:** NR  **Distance between sites:** 55km  **Recruitment location:** UK  **High/Low risk patients:** High | **Environment:** Hospital  **Operator**: Sonographer  **Training:** Training in using the telemedicine equipment was provided, as well as advanced training in fetal heart scanning.  **Technology:** Store and forward method – videos recorded on ultrasound then sent across in prearranged videoconferences. | **Environment:** Hospital  **Operator**: NR  **Training:** NR  **Technology:** Videoconferencing system (model 2500, Tandberg). | **Communication:** Internet  **Transmission:** Asynchronous  **Frame rate:** NR  **Resolution:** NR  **Bandwidth:** 384 Kb/s  **Transmission delay:** NR | A telemedicine assessment of 5 min duration was more costly than an examination in London (mean cost per referral of £206 v £74, P=0.001). However, the telecardiology service was cost neutral after 14 days and for the extended period until delivery. Travel costs for London women averaged £37 compared with £5.50 for the telemedicine referrals. |
| Malone 1998 (58) | Retrospective review of fixed and non-fixed costs associated with interpreting obstetric ultrasound examinations using both videotape and telemedicine transmission. | **Sample size:** NR  **Gestation:** 1^st^, 2^nd^ & 3^rd^  **Distance between sites:** NR  **Recruitment location:** USA  **High/Low risk patients:** NR | **Environment:** Hospital  **Operator**: Sonographer  **Training:** NR  **Technology:** Telemedicine system includes a standard desktop 486 DX133 personal computer with a personal computer-based modified Picture Tel VM 100 videocodec device, a V.35 data card, an RS366 dialing card, an Integrated Services Digital Network (ISDN) inverse multiplexer and modem, a remote control pan/tilt/zoom video camera and a speaker-phone with headsets. The personal computers are also loaded with an obstetric ultrasonography reporting system and the video monitors have touch-screen capabilities. Additionally, a standard set of still images is also obtained, and are printed on either Sony Type II UPP-110HD highdensity printing paper or Kodak Ektascan EB-1 diagnostic medical film. | **Environment:** Hospital  **Operator**: NR  **Training:** NR  **Technology:** Live telemedicine connection is made to the interpreting physician at the central facility, and both still images and the initial computer-generated report are transmitted. After reviewing these still images and the initial report, the interpreting physician directs the sonographer through relevant parts of the fetal ultrasound examination, allowing for real-time image interpretation. | **Communication:** Internet & Courier  **Transmission:** Synchronous & Asynchronous  **Frame rate:** NR  **Resolution:** NR  **Bandwidth:** 64Kb/s  **Transmission delay:** NR | For this network, the fixed costs for establishing telemedicine are $101 750. Monthly non-fixed cost savings by eliminating videotape review include $1620 to $2700 for printing still images, $1200 for courier charges and $7000 for fewer repeat ultrasound examinations. Monthly non-fixed costs for the telemedicine network are $2415. Net monthly savings in non-fixed costs for a telemedicine network are therefore $7405 to $8585, which may pay for the initial fixed costs in 12 to 14 months. |
| Mistry 2013 (64) | Telemedicine service was connected to a fetal cardiology unit in London. Aimed to estimate the longer-term cost-effectiveness of using telemedicine screening for prenatal detection of congenital heart disease (CHD). | **Sample size:** NR  **Gestation:** NR  **Distance between sites:** NR  **Recruitment location:** UK  **High/Low risk patients:** Both | **Environment:** Hospital  **Operator**: NR  **Training:** Staff were trained to use the equipment.  **Technology:** The telemedicine equipment installed in the district hospital included a videoconferencing system. | **Environment:** Hospital  **Operator**: NR  **Training:** NR  **Technology:** NR | **Communication:** Internet  **Transmission:** Synchronous  **Frame rate:** NR  **Resolution:** NR  **Bandwidth:** 384Kb/s  **Transmission delay:** NR | The probabilistic results from the model show that offering telemedicine to all standard-risk women is the dominant strategy, i.e. that the costs are lower and the QALYs are higher (that is, telemedicine is more effective). |
| **Qualitative studies** | | | | | | |
| Bidmead 2020 (31) | Qualitative findings from interviews with frontline clinicians and service users of a fetal telemedicine service. Semi-structured interviews with clinical stakeholders and service users were conducted, undertaken as part of a service evaluation. | **Sample size:** 23  **Gestation:** 1^st^ and 2^nd^  **Distance between sites:** NR  **Recruitment location:** England  **High/Low risk patients:** NR | **Environment:** Hospital  **Operator**: Sonographer  **Training:** NR  **Technology:** Videoconferencing equipment. | **Environment:** Hospital  **Operator**: Materno-fetal specialist  **Training:** NR  **Technology:** Videoconferencing equipment. | **Communication:** Internet  **Transmission:** Synchronous  **Frame rate:** NR  **Resolution:** NR  **Bandwidth:** NR  **Transmission delay:** NR | Sonographers reported four main challenges: delivering a shared consultation; the requirement to resist scanning intuitively; communications during the scan; and restricted room space. Notwithstanding, all clinicians reported that participating women were accepting of the technology. Service users reported few concerns. The main benefits of fetal telemedicine were identified as upskilled staff, increased access to specialist support and improved management of complex pregnancies. Convenience was identified as the main benefit by service users, including savings in time and money from not having to travel, take time off work, and arrange childcare. |
| Hishitani 2014 (46) | Questionnaires were sent to 14 maternity hospital staff members for qualitative assessment at the start of fetal telediagnosis and at the end of the study. | **Sample size:** 14  **Gestation:** NR  **Distance between sites:** NR  **Recruitment location:** Japan  **High/Low risk patients:** NR | **Environment:** Hospital  **Operator**: NR  **Training:** NR  **Technology:** Optical fiber transmission of recorded ultrasound images stored on a DVD or spatiotemporal image correlation (STIC) images, or real-time images between hospitals. | **Environment:** Hospital  **Operator**: NR  **Training:** NR  **Technology:** Teleconference system. | **Communication:** Internet  **Transmission:** Asynchronous & Synchronous  **Frame rate:** 30 fps  **Resolution:** NR  **Bandwidth:** 6 Mb/s  **Transmission delay:** NR | The results showed that the staff reported a significant increase in confidence in performing fetal cardiac screening (score 2.3 at start, 3.4 at study completion; P = 0.034), the rate of score increase rose with the number of telediagnoses (r = 0.72, P < 0.05), feedback from a specialist was very useful (4.4 and 4.9, respectively), and real-time image transmission was preferred over recorded images (score 3.7 vs 2.4, respectively; P = 0.042). |
| Kumar 2023 (52) | The relative affordability of SonoMobile was described as a critical enabler for a business model targeting low- and middle-income segments of the population, and for increasing quality and equity of antenatal care coverage. | **Sample size:** 61  **Gestation:** NR  **Distance between sites:** NR  **Recruitment location:** Kenya  **High/Low risk patients:** NR | **Environment:** Community  **Operator**: Nurse midwives  **Training:** Training of nurse midwives via a new curriculum developed in collaboration with the Kenya Medical Training College (KMTC) on nurse-led obstetric ultrasonography  **Technology:** The SonoMobile intervention involved training nurse-midwives to conduct point-of-care obstetric ultrasound scans in antenatal care clinics in urban informal settlements. Scan data and images were shared, using telemedicine technology, with remote sonographers. | **Environment:** Hospital  **Operator**: Sonographer  **Training:** NR  **Technology:** Telemedicine technology who reviewed scan images and data and provided reports. | **Communication:** Internet  **Transmission:** Asynchronous  **Frame rate:** NR  **Resolution:** NR  **Bandwidth:** NR  **Transmission delay:** NR | Perceived value of nurse-led obstetric ultrasonography include improving access and affordability of obstetric ultrasonography services, timely identification and referral of high-risk pregnancies, and improving awareness of appropriate antenatal care among underserved populations. The relative affordability of SonoMobile was described as a critical enabler for a business model targeting low- and middle-income segments of the population, and for increasing quality and equity of antenatal care coverage. Areas highlighted for improvement include strengthening supervision of nurse trainees, broadening the scope of nurse training, and development of clear regulatory guidelines for nurse-led obstetric ultrasonography. |
| McCrossan 2012 (61) | Study evaluated patients’ opinions on a fetal cardiology telemedicine service compared with usual outpatient care, the effect of the telemedicine consultation on maternal anxiety and its impact on travel times and time absent from work. | **Sample size:** 66  **Gestation:** 2^nd^  **Distance between sites:** NR  **Recruitment location:** Northern Ireland  **High/Low risk patients:** High | **Environment:** Hospital  **Operator**: Radiographer  **Training:** NR  **Technology:** Fetal echocardiogram transmitted to the regional centre with live guidance by a fetal cardiologist. | **Environment:** Hospital  **Operator**: Materno-fetal specialist  **Training:** NR  **Technology:** Telecommunication equipment. | **Communication:** Internet  **Transmission:** Synchronous  **Frame rate:** NR  **Resolution:** NR  **Bandwidth:** NR  **Transmission delay:** NR | Participants expressed very high satisfaction rates with fetal telecardiology, equivalent to face-to-face consultation. Telecardiology appointments were associated with significantly reduced travel times and days off work (p < 0.01). Expectant mothers expressed a clear inclination for a fetal cardiology appointment at the local hospital facilitated by telemedicine (p < 0.01). |
| Smith 2021 (76) | Reporting the introduction of a fetal ultrasound telemedicine service linking a specialist fetal medicine centre and a remote obstetric unit. | **Sample size:** 297  **Gestation:** 2^nd^ & 3^rd^  **Distance between sites:** NR  **Recruitment location:** UK  **High/Low risk patients:** High | **Environment:** Hospital  **Operator**: Sonographer  **Training:** The sonographers were provided with a pre-training manual and remote guidance via the telemedicine link from an experienced midwife sonographer based at the receiving unit.  **Technology:** Toshiba Aplio 400 ultrasound machine with a bespoke unit including a monitor, microphone, camera and codec was assembled for use in the clinical ultrasound room. A codec was installed at both sites together with a Cisco Video Conferencing (VC) unit at the recieving centre and a Polycom Group 500 VC unit at the operating unit to allow the handling of high quality images using the least bandwidth. | **Environment:** Hospital  **Operator**: Fetal cardiologist  **Training:** NR  **Technology:** The image was transmitted from the ultrasound machine at the obstetric unit through a High Definition Multimedia Interface (HDMI) cable to the HDMI port in the codec and viewed on a monitor at the receiving centre. The view at the FM centre could be alternated (using a remote control) between the ultrasound machine display during scans, to the woman and her family during pre and post-scan counselling. | **Communication:** Internet  **Transmission:** Synchronous  **Frame rate:** NR  **Resolution:** NR  **Bandwidth:** 100 Mb/s  **Transmission delay:** NR | Overall, women expressed high levels of satisfaction with the telemedicine consultation. Only two women returned responses of ‘Strongly Disagree’, one in relation to the quality of the ultrasound image and another to the question of whether she would be willing to use telemedicine to monitor her baby’s health in the future. Travel to the telemedicine consultation took a median (range) time of 20 min (4150), in comparison to an estimated journey of 230 min (120,450) to the receiving centre. |

Cm, centimetre; DVD, digital versatile disc; Fps, frames per second; km, kilometres; mb/s, megabits per second; NR, not reported; PC, personal computer; TV, television; UK, United Kingdom; USA, United States of America.
