# Supplementary material for: Teleultrasound in obstetrics: A systematic review and meta-analysis
Source: PLoS Med. 2026 Feb 6;23(2):e1004922. doi: 10.1371/journal.pmed.1004922 (PMC12900445; doi:10.1371/journal.pmed.1004922)
Supplement: S7 Table — (DOCX) [file pmed.1004922.s007.docx]

**S7 Table**: Diagnostic accuracy of teleultrasound within reporting studies, stratified by indication.

| **Diagnostic indication** | **Accuracy** | **Sensitivity** | **Specificity** | **PPV** | **NPV** |
| --- | --- | --- | --- | --- | --- |
| Congenital heart disease | 95.6% [32] | 73.7% | 96.7% | 53.9% | 98.6% |
|  | NR [33] | 100% | 79% | 21% | 100% |
|  | 96.8% [47] | 50% | 97.2% | 15.1% | 99.5% |
|  | 95.0% [55] |  |  |  |  |
|  | 97.0% [60] | 91.0% | 98.0% | 91.0% | 98.0% |
| Congenital anomaly | 95.9% [56] | 57.5% | 98.2% | 65.8% | 97.5% |
|  | 98.6% [82] | 85.0% | 99.6% | 94.4% | 98.9% |
| Cardiac, non-cardiac and unspecified | 68.7% [83] | 85.9% | 57.4% | 57.0% | 86.1% |
| Objective Liquor volume assessment | 94.6% [93] | 92.9% | 97.6% | 86.7% | 97.6% |

NPV, negative predictive value; NR, not reported; PPV, positive predictive value.
