# Supplementary material for: Teleultrasound in obstetrics: A systematic review and meta-analysis
Source: PLoS Med. 2026 Feb 6;23(2):e1004922. doi: 10.1371/journal.pmed.1004922 (PMC12900445; doi:10.1371/journal.pmed.1004922)

General

1) Amniotic fluid volume

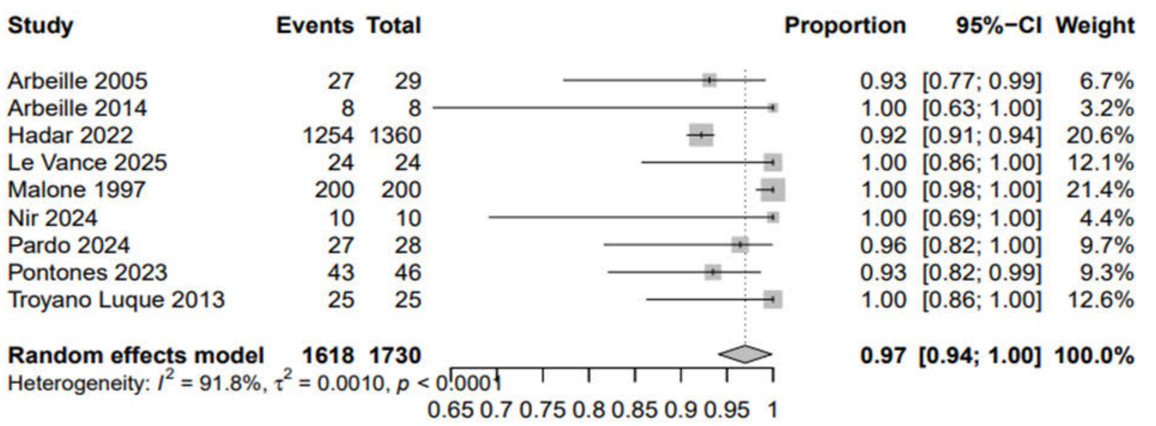

2) Crown-rump length

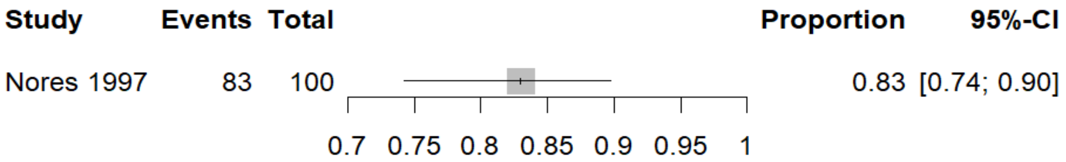

3) Fetal breathing

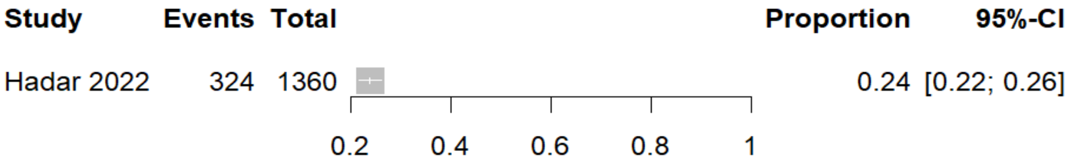

4) Fetal movements

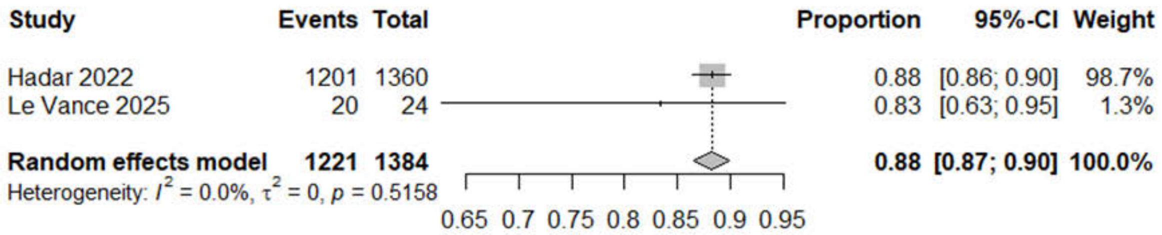

5) Fetal number

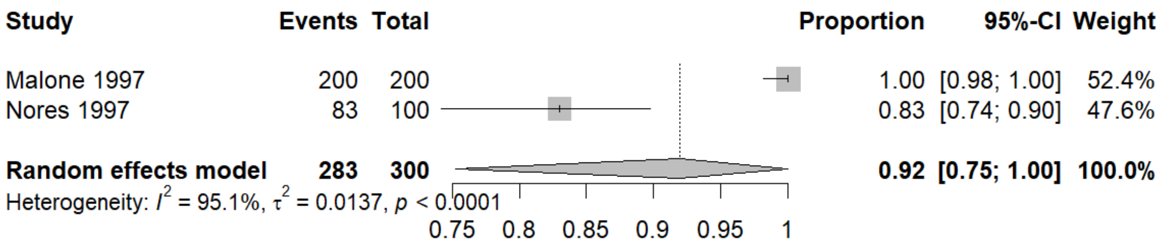

6) Fetal presentation

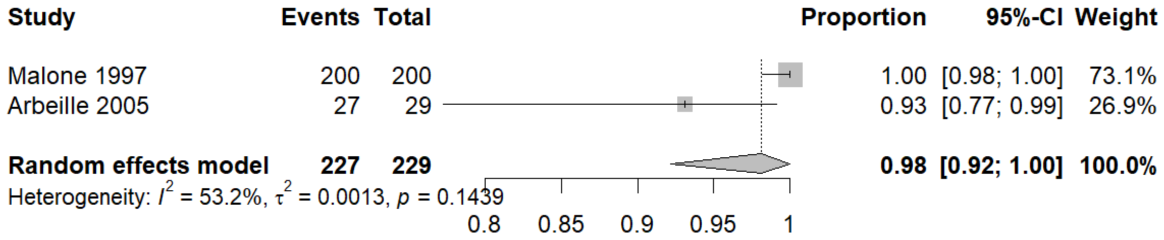

7) Overall biometric assessment

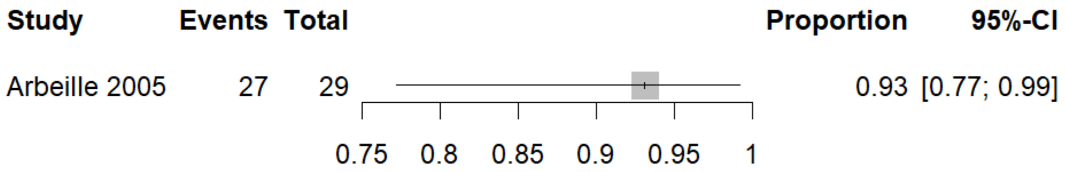

Placenta

1) Cord vessel number

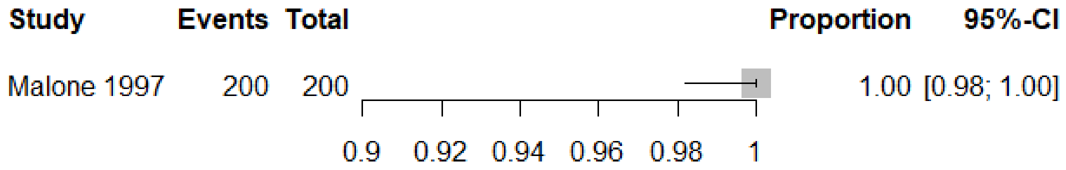

2) Cord vessel number

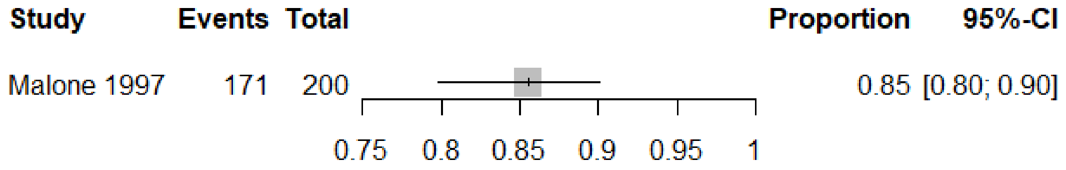

3) Placental location

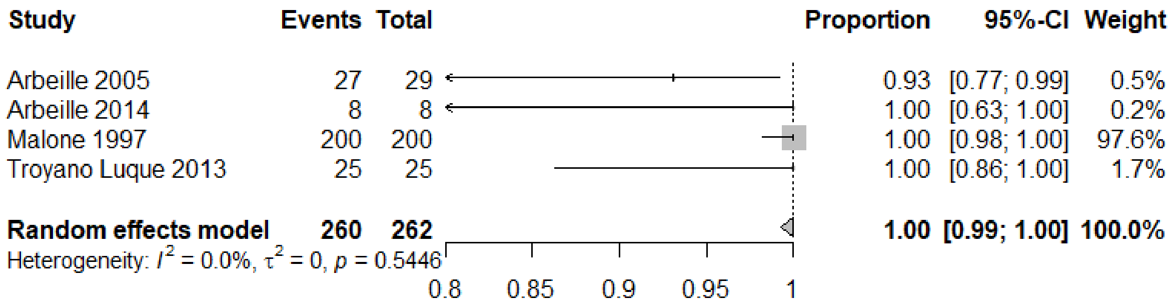

Neurological

1) Cavum septum pellucidum

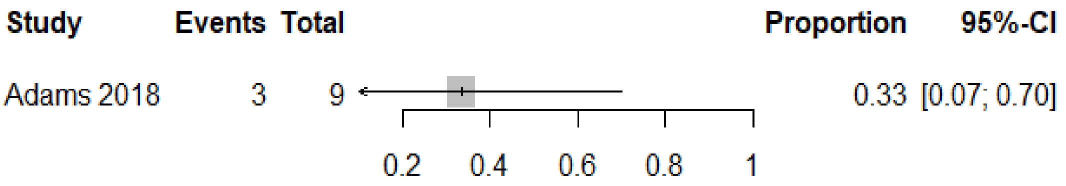

2) Cerebellum

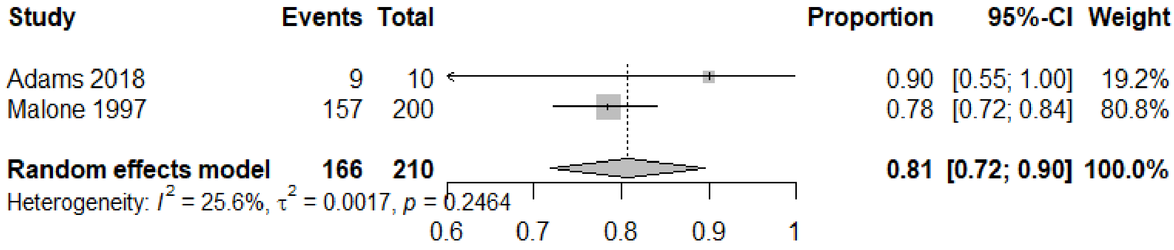

3) Choroid plexus

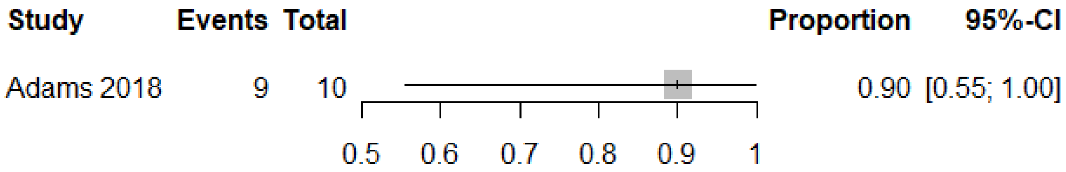

4) Cisterna magna

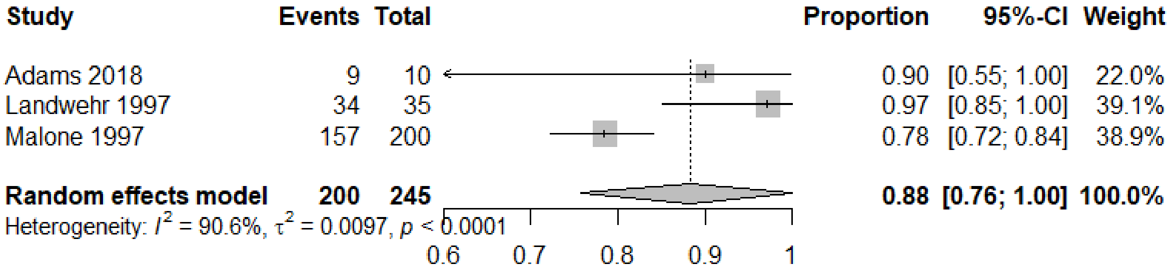

5) Cranium

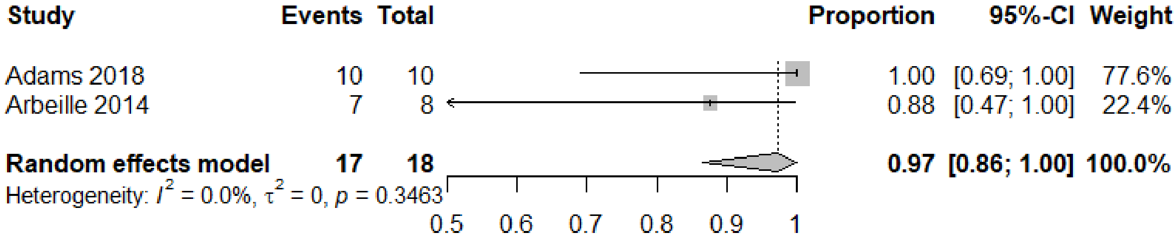

6) Midline falx

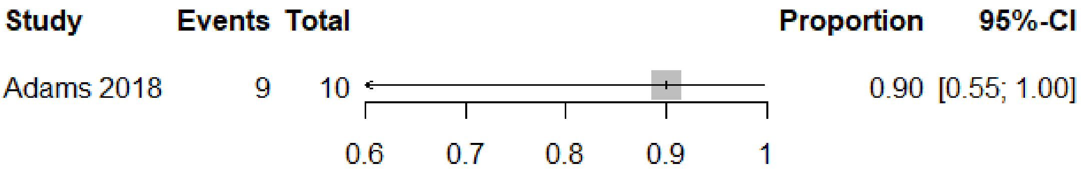

7) Nuchal thickness

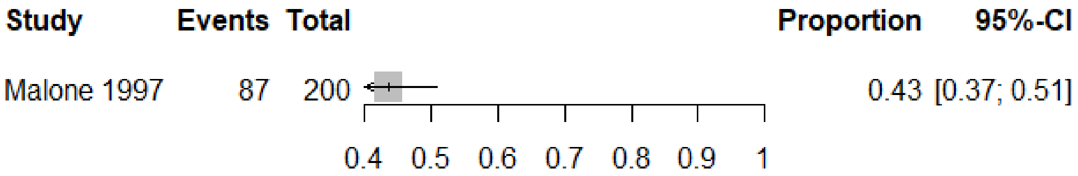

8) Ventricles

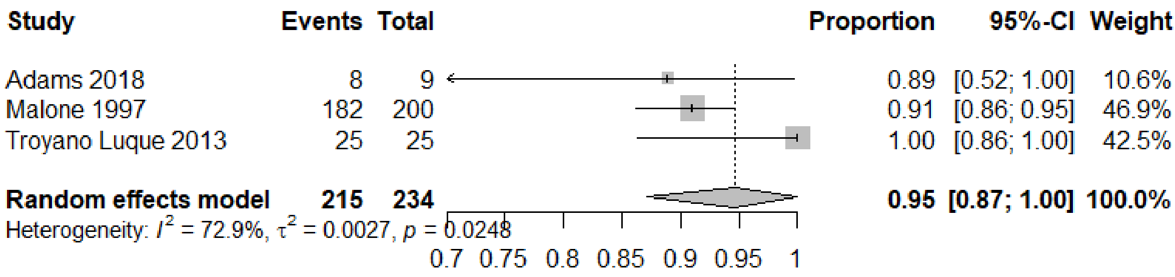

Facial

1) Face/lips

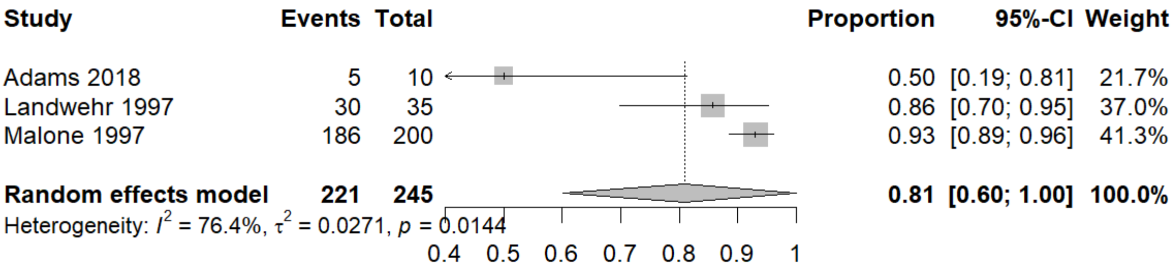

2) Nasal bone

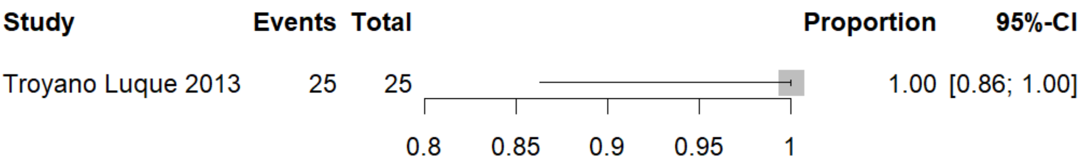

3) Orbit

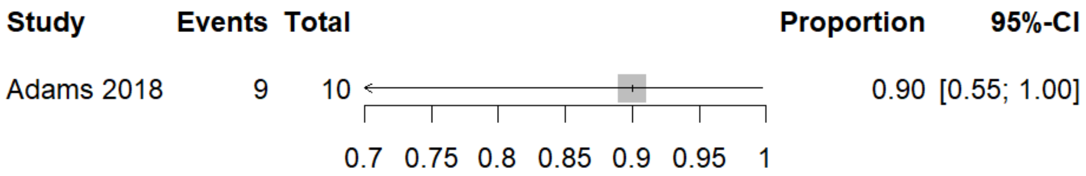

4) Profile

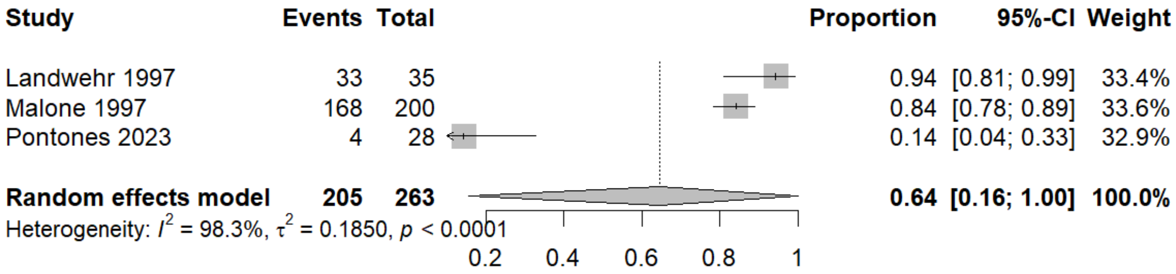

Skeletal

1) Chest

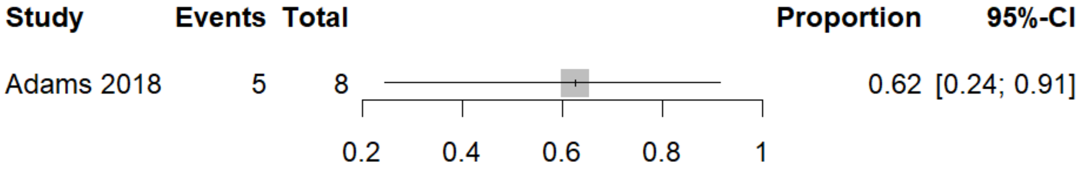

2) Femur

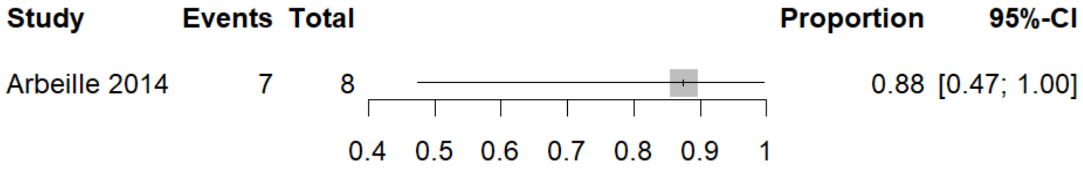

3) Spine

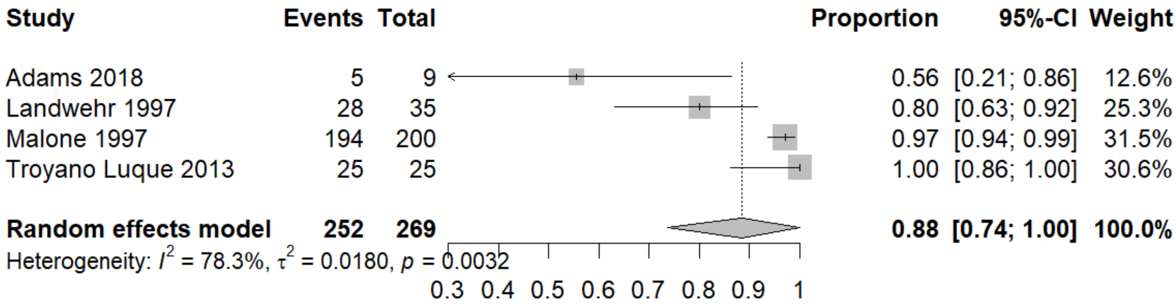

Cardiac

1) Aortic and pulmonary valves

Study	Events	Total	Proportion	95%-CI	Weight
McCrossan 2011	69	69	1.00	[0.95; 1.00]	54.0%
Vinals 2005	90	100	0.90	[0.82; 0.95]	46.0%
**Random effects model**	**159**	**169**	**0.95**	**[0.86; 1.00]**	**100.0%**

Heterogeneity: $I^2 = 90.0\%$, $\tau^2 = 0.0045$, $p = 0.0016$

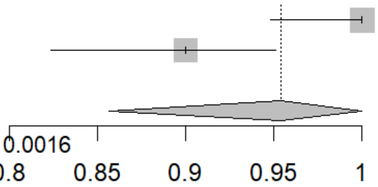

2) Aortic arch

Study	Events	Total	Proportion	95%-CI	Weight
Malone 1997	163	200	0.81	[0.75; 0.87]	35.9%
McCrossan 2011	69	69	1.00	[0.95; 1.00]	37.5%
Michailidis 2001	23	30	0.77	[0.58; 0.90]	26.6%
**Random effects model**	**255**	**299**	**0.87**	**[0.73; 1.00]**	**100.0%**

Heterogeneity: $I^2 = 95.8\%$, $\tau^2 = 0.0141$, $p < 0.0001$

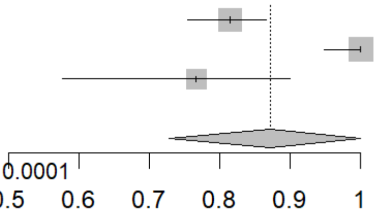

3) Atrioventricular valves

Study	Events	Total	Proportion	95%-CI	Weight
McCrossan 2011	68	69	0.99	[0.92; 1.00]	48.6%
Vinals 2005	98	100	0.98	[0.93; 1.00]	51.4%
**Random effects model**	**166**	**169**	**0.98**	**[0.96; 1.00]**	**100.0%**

Heterogeneity: $I^2 = 0.0\%$, $\tau^2 = 0$, $p = 0.7838$

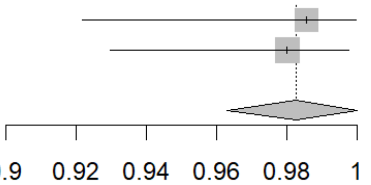

4) Axis

Study	Events	Total	Proportion	95%-CI	Weight
Adams 2018	8	10	0.80	[0.44; 0.97]	20.2%
Vinals 2005	48	50	0.96	[0.86; 1.00]	79.8%
**Random effects model**	**56**	**60**	**0.93**	**[0.80; 1.00]**	**100.0%**

Heterogeneity: $I^2 = 34.5\%$, $\tau^2 = 0.0044$, $p = 0.2166$

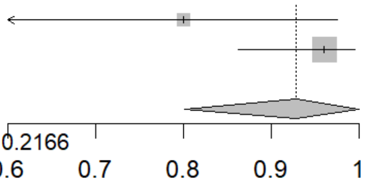

5) Cardiac chambers

Study	Events	Total	Proportion	95%-CI	Weight
Adams 2018	8	10	0.80	[0.44; 0.97]	0.0%
Day 2025	48	48	1.00	[0.93; 1.00]	0.2%
Inamura 2020	15327	15404	1.00	[0.99; 1.00]	98.8%
Landwehr 1997	32	35	0.91	[0.77; 0.98]	0.0%
Malone 1997	197	200	0.98	[0.96; 1.00]	0.4%
McCrossan 2011	69	69	1.00	[0.95; 1.00]	0.3%
Michailidis 2001	30	30	1.00	[0.88; 1.00]	0.1%
Vinals 2008	35	49	0.71	[0.57; 0.83]	0.0%
Vinals 2005	50	50	1.00	[0.93; 1.00]	0.2%
**Random effects model**	**15796**	**15895**	**0.99**	**[0.99; 1.00]**	**100.0%**

Heterogeneity: $I^2 = 69.4\%$, $\tau^2 = 0$, $p = 0.0010$

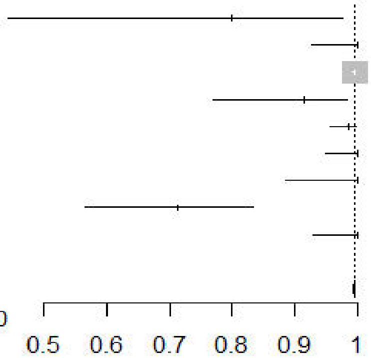

6) Cardiac Dopplers

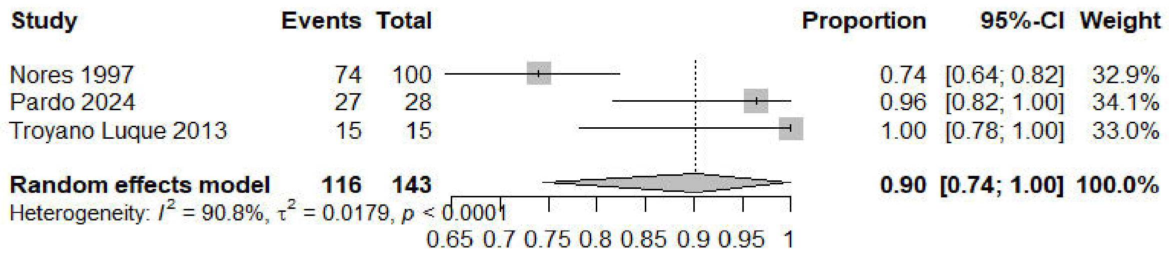

7) Crossing of the great arteries

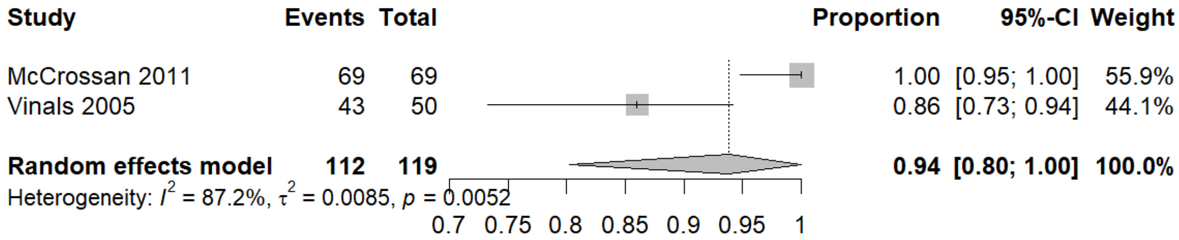

8) Crux

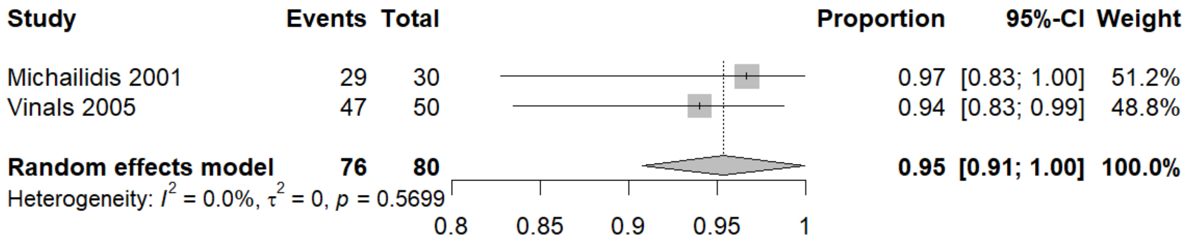

9) Ductal arch

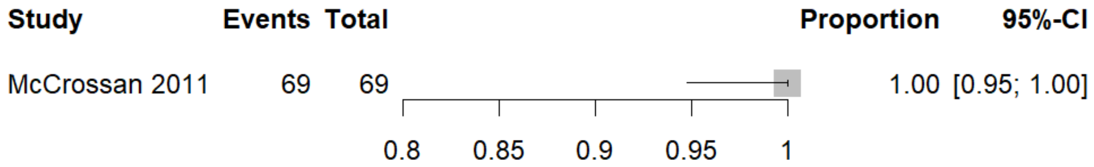

10) Fetal heart activity

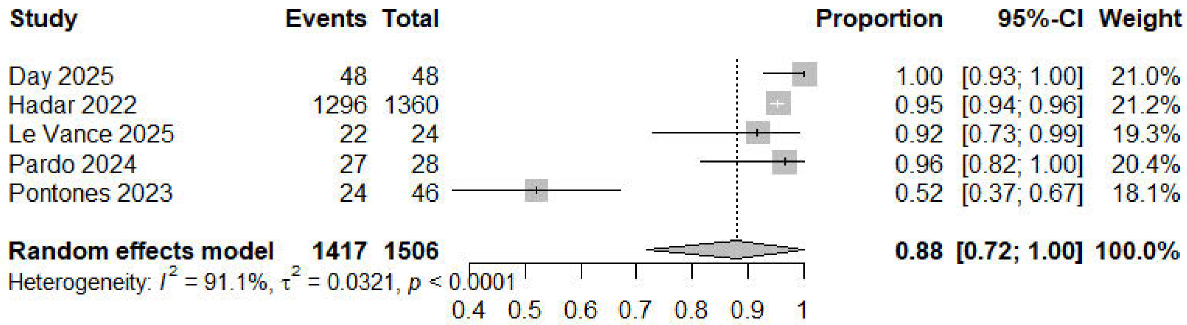

11) Foramen ovale

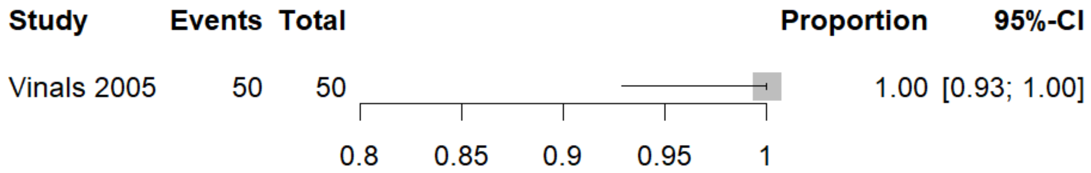

12) Inferior/superior vena cava

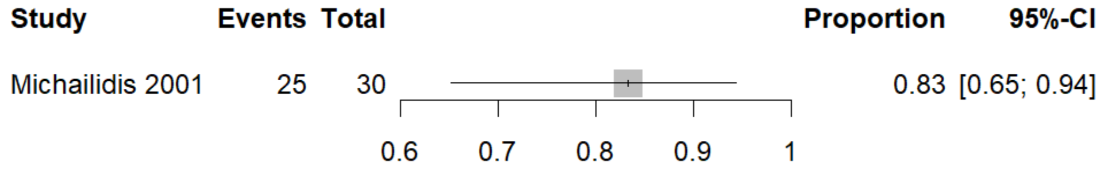

13) Left ventricular outflow tract

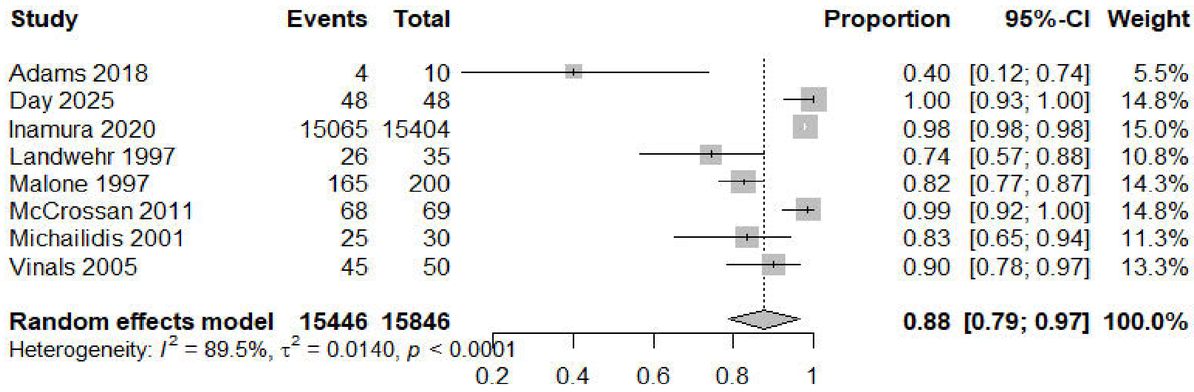

14) Primary atrial septum

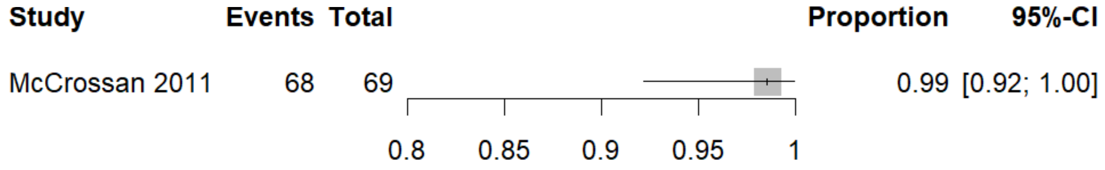

15) Pulmonary venous connection

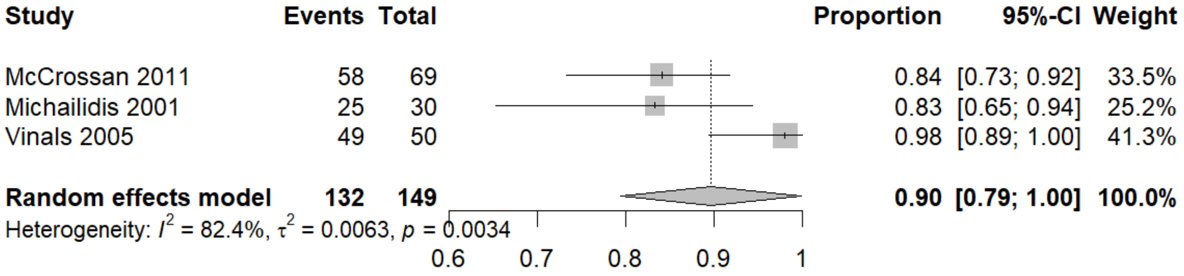

16) Right ventricular outflow tract

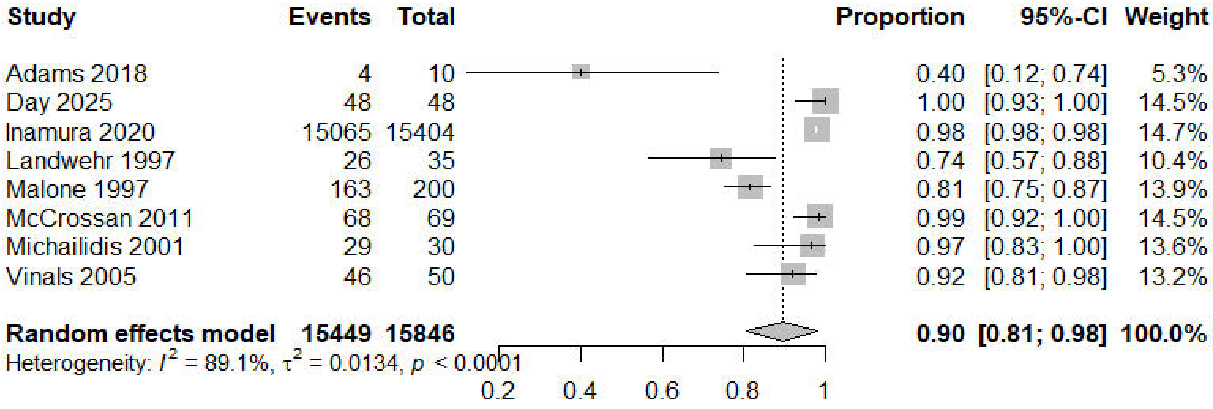

17) Situs

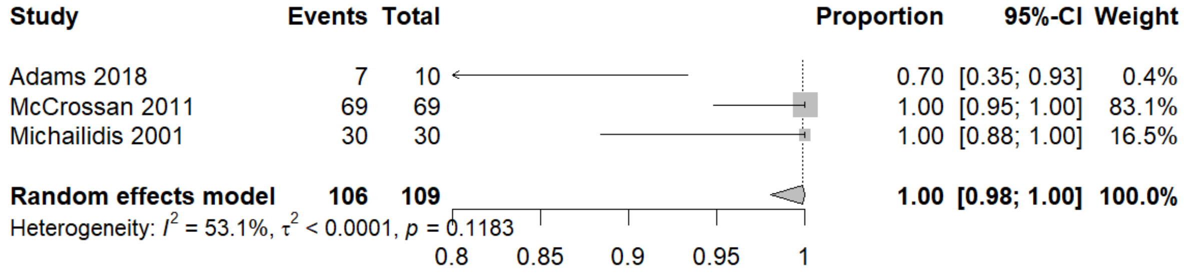

18) Three vessel view

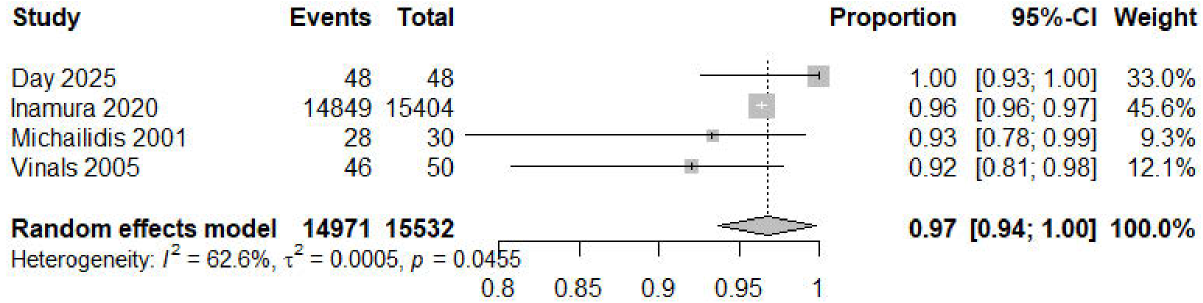

19) Three vessel trachea view

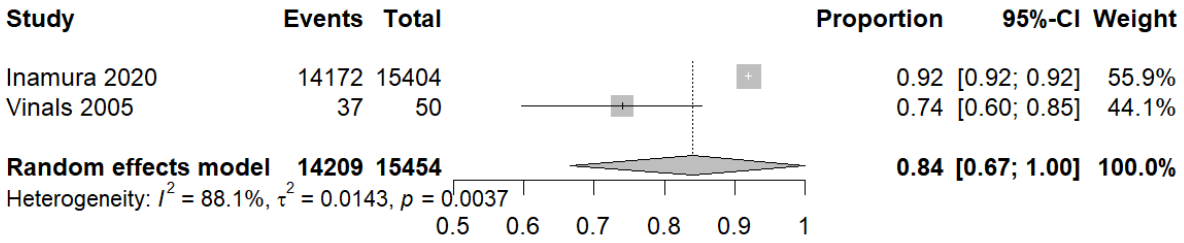

20) Ventricular septum

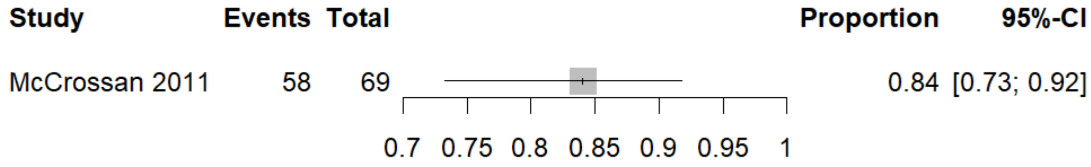

Gastric

1) Abdomen

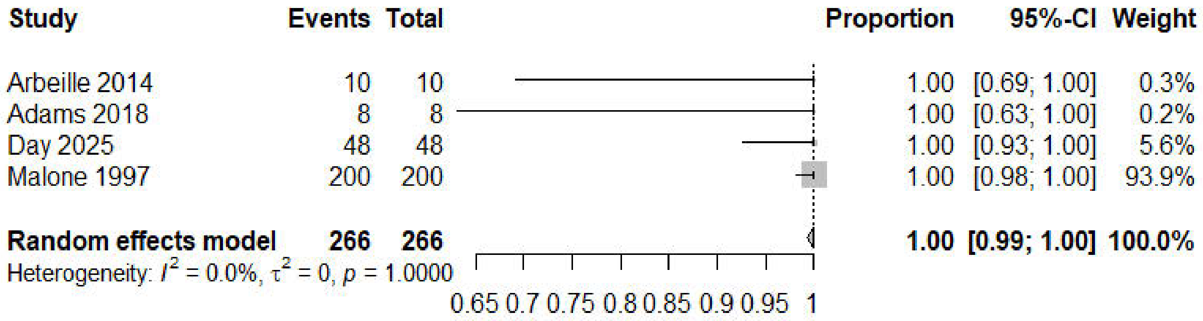

2) Cord insertion

Study	Events	Total	Proportion	95%-CI	Weight
Adams 2018	10	10	1.00	[0.69; 1.00]	1.2%
Malone 1997	198	200	0.99	[0.96; 1.00]	98.8%
**Random effects model**	**208**	**210**	**0.99**	**[0.98; 1.00]**	**100.0%**

Heterogeneity: $I^2 = 0.0\%$, $\tau^2 = 0$, $p = 0.8743$

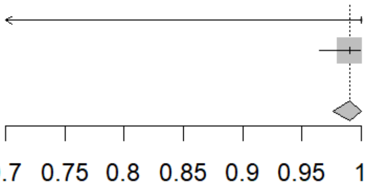

Renal

1) Bladder

Study	Events	Total	Proportion	95%-CI	Weight
Adams 2018	9	9	1.00	[0.66; 1.00]	0.3%
Malone 1997	200	200	1.00	[0.98; 1.00]	99.7%
**Random effects model**	**209**	**209**	**1.00**	**[0.99; 1.00]**	**100.0%**

Heterogeneity: $I^2 = 0.0\%$, $\tau^2 = 0$, $p = 1.0000$

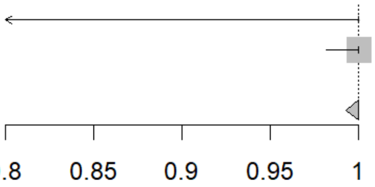

2) Kidneys

Study	Events	Total	Proportion	95%-CI	Weight
Adams 2018	5	9	0.56	[0.21; 0.86]	22.6%
Landwehr 1997	28	35	0.80	[0.63; 0.92]	36.2%
Malone 1997	200	200	1.00	[0.98; 1.00]	41.2%
**Random effects model**	**233**	**244**	**0.83**	**[0.60; 1.00]**	**100.0%**

Heterogeneity: $I^2 = 87.4\%$, $\tau^2 = 0.0335$, $p = 0.0004$

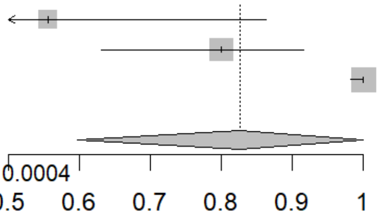

Reproductive

1) Genitalia

Study	Events	Total	Proportion	95%-CI	Weight
Landwehr 1997	24	35	0.69	[0.51; 0.83]	42.9%
Malone 1997	177	200	0.88	[0.83; 0.93]	57.1%
**Random effects model**	**201**	**235**	**0.80**	**[0.61; 0.99]**	**100.0%**

Heterogeneity: $I^2 = 83.2\%$, $\tau^2 = 0.0165$, $p = 0.0147$

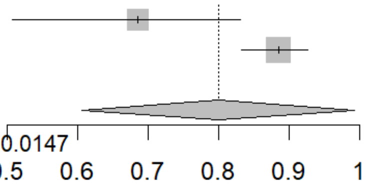

Extremities

1) Arm

Study	Events	Total	Proportion	95%-CI	Weight
Adams 2018	10	10	1.00	[0.69; 1.00]	5.7%
Malone 1997	190	200	0.95	[0.91; 0.98]	94.3%
**Random effects model**	**200**	**210**	**0.95**	**[0.92; 0.98]**	**100.0%**

Heterogeneity: $I^2 = 0.0\%$, $\tau^2 = 0$, $p = 0.4394$

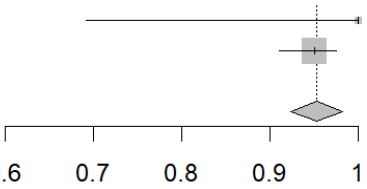

2) Foot

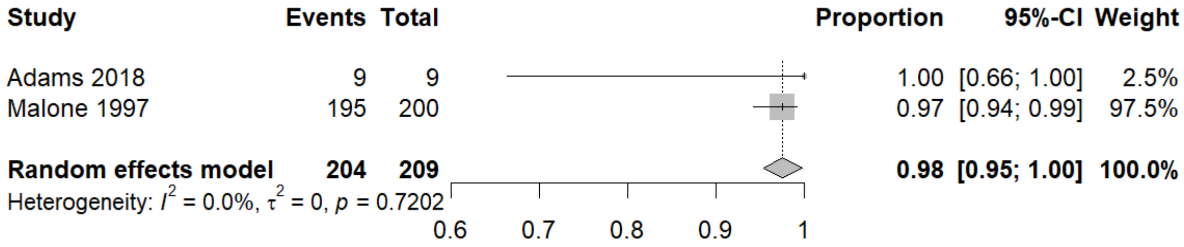

3) Hand

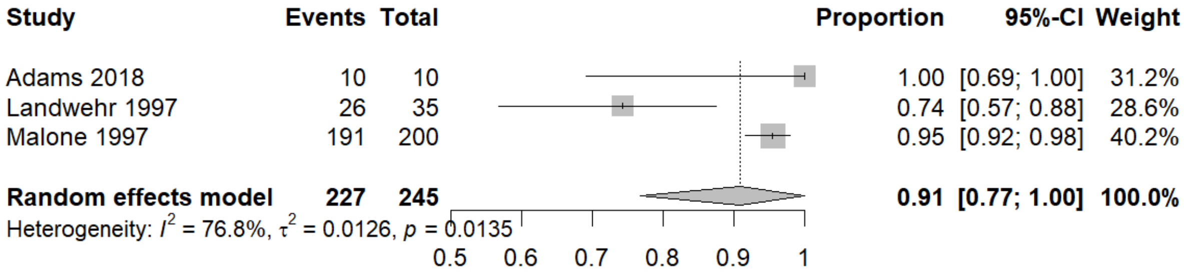

4) Leg

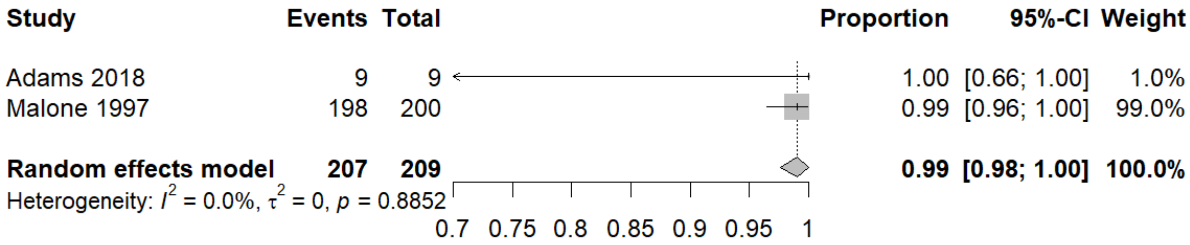

Supplement: S1 Fig — (PDF) [file pmed.1004922.s008.pdf]
