# Supplementary figures and images for: Teleultrasound in obstetrics: A systematic review and meta-analysis
Source: PLoS Med. 2026 Feb 6;23(2):e1004922. doi: 10.1371/journal.pmed.1004922 (PMC12900445; doi:10.1371/journal.pmed.1004922)

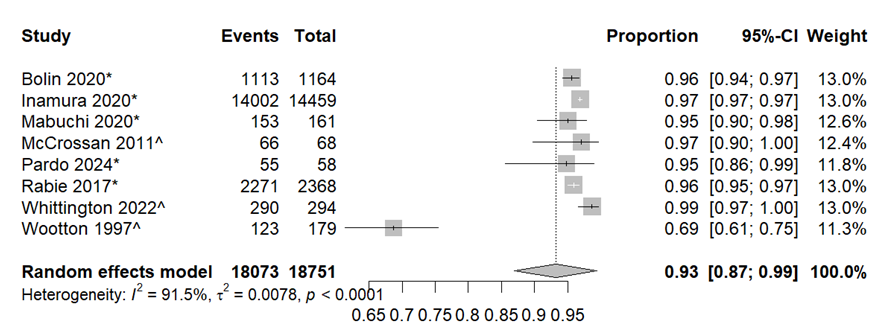

Supplement: S2 Fig — (*) represents the reference standard was conventional in-hospital ultrasound. (^) represented the reference standard was postnatal diagnosis. (TIF) [file pmed.1004922.s009.tif]
