# Supplementary material for: Teleultrasound in obstetrics: A systematic review and meta-analysis
Source: PLoS Med. 2026 Feb 6;23(2):e1004922. doi: 10.1371/journal.pmed.1004922 (PMC12900445; doi:10.1371/journal.pmed.1004922)
Supplement: Attachment — Submitted filename: levance.pdf [file pmed.1004922.s011.pdf]

This paper reports a systematic review with meta–analysis of studies of teleultrasound in obstetrics.

I was asked for a methodological report and I interpret that to include all aspects of the design and conduct of the study. I understand that a previous version of this was officially rejected but with the possibility of submitting a new updated version. I did see the previous version but I have not looked at my comments on it and review this one from scratch.

Points of detail

Page 8 Proper citation for any R packages gives authors at least some credit for their work. (Disclaimer: I am a CRAN author but almost certainly of different packages.)

Page 8 Accuracy is used in different fields in different ways. A global proportion does not tell us about the different ways an error can occur: missing a true case or over–diagnosing the well.

Page 8 The word configured does not seem quite right here. Would displayed or presented not be better?

Page 8 The authors of the original paper on I^2 Higgins and Thompson (2002) themselves stated

> No universal rule could cover definitions for 'mild', 'moderate' or 'severe heterogeneity [. . .]

Also worth noting the concerns of Rücker et al. (2008). There is a further issue that with the small numbers of studies included here estimates of I^2 and τ^2 will be very imprecise.

Page 9 A list of excluded studies is very helpful for later researchers and meta–analysts.

Page 10 Compared with many systematic reviews that I see this is an impressive spread of countries. Incidentally it is Israel not Isreal.

Page 10 I think the caption use of representation is better than dispersion here.

Page 11 Without reading the original article, and maybe not even then, it is hard to know whether changing the intervention and outcomes was a justified response to circumstances. If it is known perhaps report more detail here?

Page 12 Curious that studies reported distance, which I would not have expected to matter, but not many other things like bandwidth and compression technology which I would have expected to matter. Nothing the present authors can do about this obviously.

Page 14 I found Figure S1 rather easier to read than Figure 7. Perhaps split Figure 7?

Page 14 and Table 2 This is probably my ignorance of obstetrics but what does it mean to identify a structure? Surely if they saw a fetal kidney they would know what it was? If this is obvious to practitioners then my comment can be ignored.

Page 16 Stating that overall teleultrasound is non–inferior is rather restrained since Figure 7 if I read it correctly suggests it was superior. Having said that it seems to me that the overall value is less important here than the values per structure the vast majority of which do show non–inferiority.

Page 31 No doubt rigorous guidelines and appropriate supervision are important in LaMIC but is that not also true world–wide? At least part of the recent controversy in the UK over physician assistant was couched in terms of people being asked to work outside the limits of their skill set. I do not think the article would suffer if this was removed or toned down.

Page 40 Saying the largest is dangerous. In fact page 43 is more circumspect here.

Points of more substance

Table S7 seems to bear out my point about different meanings of accuracy. Note for instance that ref 44 has very similar accuracy to ref 57, 96.8 versus 97, but very different sensitivities, 50 versus 91.. If the raw frequencies are available the usual method here is a bivariate meta–analysis of sensitivity and specificities although there are other options which may be preferred if there are few studies. Note you cannot just meta–analyse the sensitivity and specificity separately since they are correlated between studies although independent within studies. The vignette for the R package mada `https://cran.r-project.org/package=mada` outlines the issues and presents various options.

Summary

Mostly points for clarification and expansion. I do think we need to know more about what authors of the primary studies meant by accuracy

Michael Dewey

References

J P T Higgins and S G Thompson. Quantifying heterogeneity in a meta–analysis. *Statistics in Medicine*, 21:1539–1558, 2002.

G Rücker, G Schwarzer, J R Carpenter, and M Schumacher. Undue reliance on I^2 in assessing heterogeneity may mislead. *BMC Medical Research Methodology*, 8(79), 2008.
