# Supplementary material for: Teleultrasound in obstetrics: A systematic review and meta-analysis
Source: PLoS Med. 2026 Feb 6;23(2):e1004922. doi: 10.1371/journal.pmed.1004922 (PMC12900445; doi:10.1371/journal.pmed.1004922)
Supplement: Attachment — Submitted filename: Response to reviewers.docx [file pmed.1004922.s013.docx]

Department of Applied Health Sciences

University of Birmingham

4^TH^ November 2025

RE: Manuscript PMEDICINE-D-25-03073R1

**Dear Dr Alexandra Tosun,**

Thank you very much for your invaluable input and for considering our article for publication in PLOS Medicine. Please find the reviewer feedback below, with our responses following.

**Reviewer 1**

*Points of detail*

1. Page 8: Proper citation for any R packages gives authors at least some credit for their work. (Disclaimer: I am a CRAN author but almost certainly of different packages.)
   - Thank you very much for your comment. I have included the appropriate citation for the R software, which has replaced the old citation number 17 (line 192 of the manuscript with tracked changes and line 191 of the clean manuscript). The reference list has been updated.
2. Page 8: Accuracy is used in different fields in different ways. A global proportion does not tell us about the different ways an error can occur: missing a true case or over diagnosing the well.
   - Thank you for your comment. Additional information has been written in section 3.4.2 (diagnostic accuracy) regarding what the included studies meant by accuracy. This is situated on lines 409-417 on the manuscript with tracked changes and lines 409-414 of the clean manuscript.
3. Page 8 The word configured does not seem quite right here. Would displayed or presented not be better?
   - Thank you for your comment. I have changed the word to presented, which is on line 217 of the manuscript with tracked changes.
4. Page 8 The authors of the original paper on I 2 Higgins and Thompson (2002) themselves stated
   - No universal rule could cover definitions for ‘mild’, ‘moderate’ or ‘severe heterogeneity [. . . ]
   - Also worth noting the concerns of Rucker et al. (2008). There is a further issue that with the small numbers of studies included here estimates of I^2^ and τ^2^ will be very imprecise.
   - Thank you for your comment. We have moved the definitions of ‘mild’, ‘moderate’ and ‘severe’ form the manuscript on lines 196-198 of the manuscript with tracked changes
5. Page 9 A list of excluded studies is very helpful for later researchers and meta–analysts.
   - Thank you very much for your kind comment.
6. Page 10 Compared with many systematic reviews that I see this is an impressive spread of countries. Incidentally it is Israel not Isreal.
   - Thank you very much for your kind comment.
   - Israel has now been spelt correctly throughout the manuscript.
   - The supplementary documents have been checked and updated accordingly.
7. Page 10 I think the caption use of representation is better than dispersion here.
   - Thank you for your comment. We have changed the word to representation on line 247 of the tracked changes manuscript.
8. Page 11 Without reading the original article, and maybe not even then, it is hard to know whether changing the intervention and outcomes was a justified response to circumstances. If it is known perhaps report more detail here?
   - Thank you for your comment. Regarding the risk of bias assessment for the paper in question, some concerns arose regarding deviation from the intended intervention. This referred to non-adherence to the assigned intervention regime, which may have affected the outcomes. Furthermore, regarding the measurement of the outcome, the assessors concluded that knowledge of the intervention received could potentially influence the assessment of the specific outcomes used in this study. These two domains were subsequently graded with some concern.
9. Page 12 Curious that studies reported distance, which I would not have expected to matter, but not many other things like bandwidth and compression technology which I would have expected to matter. Nothing the present authors can do about this obviously.
   - Thank you for your comment. The authors are in agreement with you regarding this finding.
10. Page 14 I found Figure S1 rather easier to read than Figure 7. Perhaps split Figure 7?
    - Thank you for your comment. We have split Figure 7 into 2 images to improve readability with each labelled A or B.
11. Page 14 and Table 2 This is probably my ignorance of obstetrics but what does it mean to identify a structure? Surely if they saw a fetal kidney they would know what it was? If this is obvious to practitioners then my comment can be ignored.
    - Thank you for your comment. In the majority of papers, identification of a structure was achieved if the acquired teleultrasound images were comparative to what would normally be achieved in routine clinical practice. We have added this information to improve clarity within the manuscript on lines 353-355 with tracked changes and lines 350-352 of the clean manuscript.
12. Page 16 Stating that overall teleultrasound is non–inferior is rather restrained since Figure 7 if I read it correctly suggests it was superior. Having said that it seems to me that the overall value is less important here than the values per structure the vast majority of which do show non–inferiority.
    - Thank you for your comment. Additionally, wording has been added to this section on lines 372-373 of the manuscript with tracked changes (line 368-370 of the clean manuscript) to highlight that 18 out of the 20 structures assessed demonstrated non-inferiority with the overall pooled effect then demonstrated.
13. Page 31 No doubt rigorous guidelines and appropriate supervision are important in LaMIC but is that not also true world–wide? At least part of the recent controversy in the UK over physician assistant was couched in terms of people being asked to work outside the limits of their skill set. I do not think the article would suffer if this was removed or toned down.
    - Thank you for your comment. We have removed this from the manuscript.
14. Page 40 Saying the largest is dangerous. In fact, page 43 is more circumspect here
    - Thank you for your comment. We have removed these statements from the manuscript.

*Points of more substance*

15. Table S7 seems to bear out my point about different meanings of accuracy. Note for instance that ref 44 has very similar accuracy to ref 57, 96.8 versus 97, but very different sensitivities, 50 versus 91. If the raw frequencies are available the usual method here is a bivariate meta–analysis of sensitivity and specificities although there are other options which may be preferred if there are few studies. Note you cannot just meta–analyse the sensitivity and specificity separately since they are correlated between studies although independent within studies. The vignette for the R package mada https:// cran.r-project.org/package=mada outlines the issues and presents various options
    - Thank you for your comment. We have now undertaken a meta-analysis, using the available raw frequencies and R mada package, employing a Hierarchical Summary Receiver Operating Characteristic (HSROC) model. This new Figure has now replaced the old Figure 8 (proportional meta-analysis of diagnostic accuracy), which has been filed into the supplementary material alongside Table S7. Additional wording in the methodology and results sections can be found on lines 200-210 and 429-438 respectively of the manuscript with tracked changes (lines 197-207 and 423 – 432 of the clean manuscript). The reference list has been updated accordingly.

*Summary*

Mostly points for clarification and expansion. I do think we need to know more about what authors of the primary studies meant by accuracy

- Thank you for your comment. Additional information has been written in section 3.4.2 (diagnostic accuracy) regarding what the included studies meant by accuracy. This is situated on lines 409-417 of the manuscript with tracked changes and lines 409-414 of the clean manuscript.

**Reviewer 2**

The authors have performed the requested systematic review and meta-analysis, which has strengthened the main conclusions of the revised version of the manuscript. Therefore, I confirm my initial recommendation to accept it.

- Thank you very much for your kind comment.

**Reviewer 3**

Having examined this revised version, it is clear that the authors have addressed the main issues raised previously. The results, which were formerly lengthy and somewhat repetitive, are now better structured, supported by summary tables and figures, and enriched by the addition of a meta-analysis. This is the most important improvement, moving the work beyond narrative synthesis to provide quantitative pooled evidence on feasibility, diagnostic accuracy, and non-inferiority compared with conventional ultrasound. The clinical indications are more clearly delineated, with diagnostic accuracy now stratified by anatomical structure and indication. Risk of bias assessments, previously relegated to supplementary materials, are integrated into the main text, which strengthens the critical appraisal. The discussion has also been broadened to include ethical, regulatory, and equity considerations, with greater attention to barriers such as infrastructure, digital literacy, and social determinants of uptake. The roadmap for future research is more specific and operational, identifying methodological standards and clinically relevant outcomes.

- Thank you very much for your kind comment.

Some limitations remain, especially the reliance on studies of low methodological quality and a still somewhat superficial treatment of ethical and regulatory issues, but overall, the manuscript now represents a rigorous and substantially improved contribution. It is the most comprehensive and quantitatively informed review to date on obstetric teleultrasound and is in my view suitable for publication once minor refinements are made.

- Thank you for your comment. We have expanded on the ethical and regulatory issues on lines 672-701 and 722-729 of the manuscript with tracked changes (lines 662 -685 and lines 707-714 of the clean manuscript). We hope this provides a more comprehensive assessment.

**Reviewer 4**

The authors seek to perform a review to evaluate if teleultrasound is feasible, acceptable, diagnostically accurate and cost effective for antenatal care.

Main concern: Of the 5781 papers screened, 126 full texts were reviewed, and 61 studies were selected for inclusion. I am impressed with the number of articles that have been left out and that this process has not been performed adequately. For example, looking at Table S4, I found the work by Toscano et al "69 Testing a telediagnostic obstetric ultrasound system without a trained sonographer in a low-resource Peruvian clinic". This work was left out since it was a conference abstract. However, there is a full text article appears that can be found in:
Toscano, Marika, et al. "Testing telediagnostic obstetric ultrasound in Peru: a new horizon in expanding access to prenatal ultrasound." BMC Pregnancy and Childbirth 21.1 (2021): 328.
Furthermore, this article appears in other reviews about the same topic, including Kariman et al, a work which is cited as [3] in references of this submission. I strongly suggest the authors validate thoroughly that their inclusion/exclusion criteria have been followed correctly.

- Thank you for your comment. The authors have gone through the excluded study list, assessing the papers and searching for any available full text, if the paper was a conference abstract. This has resulted in the addition of two further full texts to be added to this review. The papers have been integrated into the manuscript and relevant supplementary information. A total of 63 studies are included in this review, and the reference lists have been updated accordingly.

The article presents an adequate organization, presenting a table with the articles considered for the results and discussion of each section. Something that I found odd is that at the beginning of each section, the authors referenced all the considered articles. However, this list of references does not match exactly the articles presented in the table of that section. For instance, in Section 3.4.5 Three- and four-dimensional ultrasound, they mentioned they investigated 5 studies [23,39,52,60,63]. However, in Table 6, they show studies [22,23,39,52,60,63]. Same thing happens in Section 3.4.7 Low- and middle-income countries. At the beginning of the Section, they introduced studies [35,38,47,49,76,78]. However, the Table 8 shows [37,38,47,49,76,78]. Please, verify.

- Thank you for your comment. Regarding section 3.4.5, which is split into 3-dimensional and 4-dimensional ultrasound, each paragraph contains the references relating to that technology (5 references for 3-dimensional and 3 references for 4-dimensional respectively). As such, there are 8 total references in the summary table (Table 6). Regarding section 3.4.7 (low- and middle-income countries), the first reference has been updated to ensure it is the right number, and this is in accordance with Table 8. All other tables have been checked to ensure clarity.

In addition, I believe it is also important to differentiate the different clinical applications of tele-ultrasound in antenatal care. Some of the works presented aim to give a final diagnosis, whereas others target an initial screening and adequate referral to the hospital. This will pose different requirements in equipment, connectivity, operator training, etc. Could this explain your wide range of requirements showed in equipment cost or bandwidth needed? Could this also affect the discussion of image quality?

- Thank you for your comment. Delineation of included studies by clinical application has been written on lines 315-320 of the tracked changes manuscript (lines 312-317 of the clean manuscript).
- Clinical application on cost has been written on lines 598-600 of the tracked changes manuscript (lines 589-591 of the clean manuscript).
- The clinical application on bandwidth requirements has been written on lines 396-399 of the tracked changes manuscript (line 393-396 of the clean manuscript).
- Wording in the discussion has been written on lines 620-622 of the tracked changes manuscript (lines 611-613 of the clean manuscript).

Regarding equipment and besides the previous point, it would be important to weigh the year of publication and accessibility (including cost) of the equipment in the analysis. Technology and equipment have evolved considerably from 1995 to 2024. Same consideration should be considered for bandwidth.

- Thank you for your comment. Implementation cost by year of publication has been written on lines 587-593 of the tracked changes manuscript (line 578-583 of the clean manuscript), also taking into consideration the volume of scans performed.
- Chosen bandwidth and year of publication has been discussed on lines 393-396 of the tracked changes manuscript (lines 390-393 of the clean manuscript) and has been commented in the discussion on lines 620-622 of the tracked changes manuscript (611-613 of the clean manuscript).

Regarding operator training and required skills in LMICs, besides the previous point of the target medical application, it might also be interesting to add a comment about the use of AI complementing blind sweeps for widespread use. Especially, since there is a good part of the discussion focused on this point.

- Thankyou for your comment. Information regarding the use of AI in the setting of LMICs has been written on lines 722-729 of the tracked changes manuscript (lines 707-714 of the clean manuscript).
