# Supplementary material for: Teleultrasound in obstetrics: A systematic review and meta-analysis
Source: PLoS Med. 2026 Feb 6;23(2):e1004922. doi: 10.1371/journal.pmed.1004922 (PMC12900445; doi:10.1371/journal.pmed.1004922)
Supplement: Attachment — Submitted filename: Response to reviewers v2.docx [file pmed.1004922.s014.docx]

Department of Applied Health Sciences

University of Birmingham

20^TH^ January 2026

RE: Manuscript PMEDICINE-D-25-03073R1

**Dear Dr Alexandra Tosun,**

Thank you very much for your invaluable input and for considering our article for publication in PLOS Medicine. Please find the reviewer feedback below, with our responses following.

Comments from Reviewers:

Reviewer #1 (Michael Dewey): The authors have addressed all my points.


Reviewer #4: Authors have addressed all my previous comments.

Thank you very much for your kind comments.

We note the remaining comments are from the editors. Please see our responses to each comment in an additional document attached to this submission.

Kind regards,

Dr Jack Le Vance
